# Diazirines Beyond Photoaffinity Labeling: A Comprehensive Overview of Applications in Biological Sciences, Materials Chemistry, and NMR‐Spectroscopy

**DOI:** 10.1002/anie.202514105

**Published:** 2025-12-19

**Authors:** Dominik Schnalzer, Viktor Savic, Clemens Cziegler, Michael Schnürch, Marko D. Mihovilovic

**Affiliations:** ^1^ Institute of Applied Synthetic Chemistry, TU Wien Getreidemarkt 9/163 Vienna 1060 Austria

**Keywords:** Bioadhesives, Carbene sources, Diazirines, Materials science, Polymer crosslinking

## Abstract

Diazirines are well‐known for their role in photoaffinity labeling (PAL) probes, acting as reactive groups to fish out proteins for drug target identification or tag installation. Their key asset lies in the precise activation through UV light, heat, or voltage, and in the ability to covalently bind to organic materials. However, the versatility of diazirines extends beyond this application. It was demonstrated that diazirines act as diverse entities, functioning as bridging units to connect different materials or reinforce polymers for applications such as bulletproof armor. Some facilitate blending of non‐mixable polymers, allowing the disassembly for recycling. Others serve as glues, aiding wound healing, or immobilize molecules on surfaces, creating new material and surface properties. Some contribute to the fabrication of elastic electronic materials, and others function as magnetic resonance imaging (MRI) biolabels without the need for a “conventional hook function.” This review intends to showcase diazirines beyond PAL and provide an overview of these applications, often combined across different fields. In this regard, the chemistry of diazirines demonstrates how different disciplines draw inspiration from one another, disclosing exciting new methodologies. This review intends to inspire the development of further innovative methods and applications.

## Graphical abstract

Diazirines are three‐membered, nitrogen‐containing heterocycles that decompose under light or heat to generate carbenes. For the past two decades, they have served mainly as minimally invasive tags in photoaffinity labeling (PAL). More recently, their value as versatile carbene precursors has been recognized. This review highlights their applications beyond PAL in biology, materials chemistry, and NMR spectroscopy.

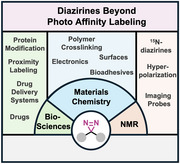

## 1 Introduction

Diazirines are three‐membered, nitrogen‐containing heterocycles that were first synthesized in 1960.^[^ ^1^ ^]^ They readily decompose upon irradiation with light or elevated temperature into molecular nitrogen and a reactive carbene species. The notorious reactivity of this species is responsible for the formation of a covalent bond with X─H (X═C, N, O, or S) or C─C bonds originating from a nearby molecule via insertion,^[^ ^2^ ^]^ making the carbene highly desirable for proximity labeling applications, such as photoaffinity labeling (PAL).^[^ ^3^, ^4^ ^]^

**Figure 1:**
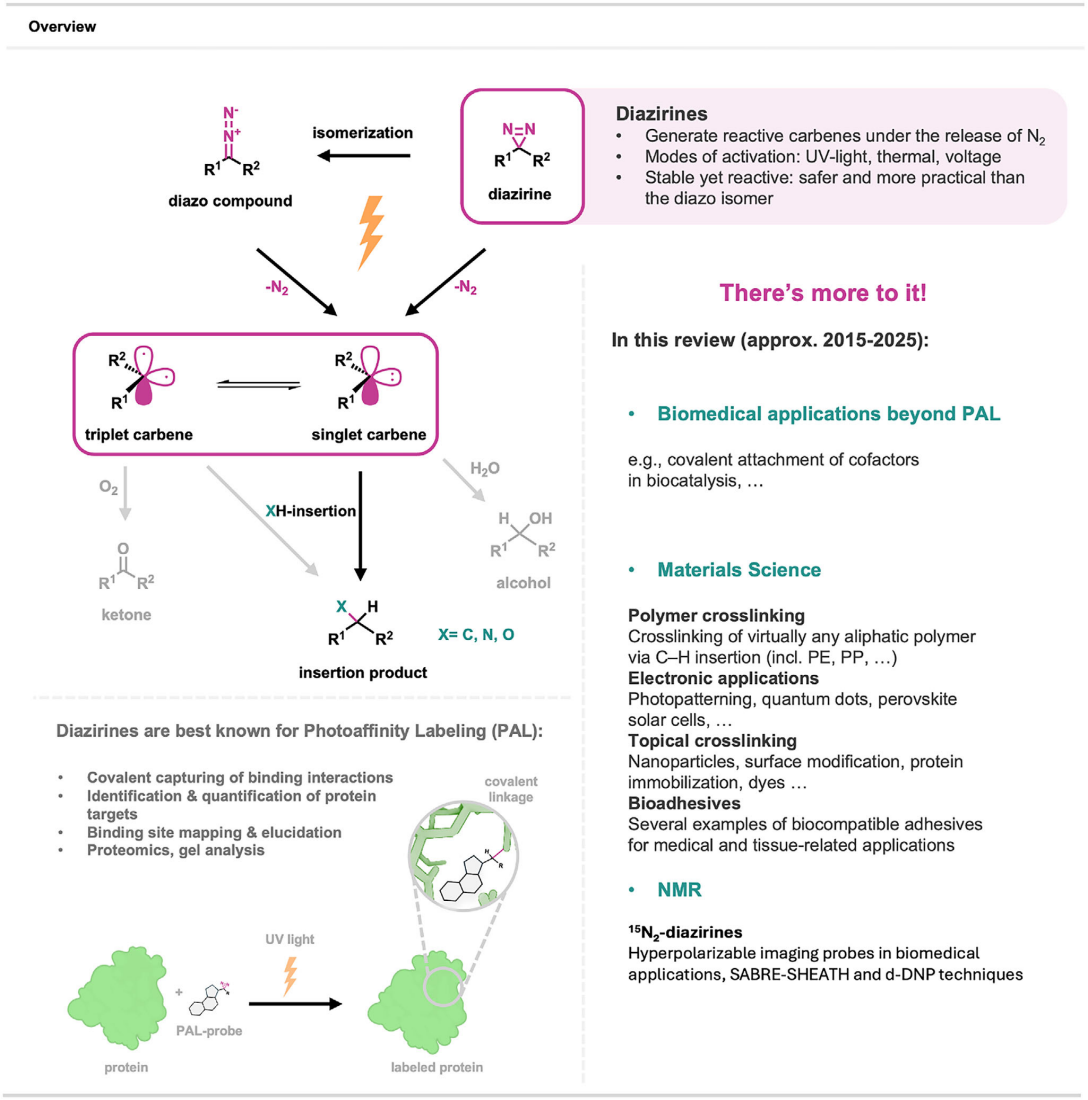
Overview on activation mechanisms of diazirines, their role in PAL, and the contents of this review. Created with BioRender.com.

Rising through the ranks to become one of the cornerstone methods in chemical biology, PAL employs a bioactive small molecule tagged with a functional group capable of carbene generation upon irradiation and subsequent covalent linkage to targeted biomolecules. The formed stable conjugates can then be analyzed with a rich palette of methods commonly used in chemical biology (e.g., mass spectrometry, gel electrophoresis, and others), allowing for studying small molecule interactions in a given biological system. Alongside diazirines, PAL applications make use of diazo compounds, aryl azides, and benzophenones, each offering unique advantages but also significant limitations such as instability, the requirement for short‐wavelength irradiation, or steric bulk that reduces general applicability. More recently, aryl tetrazoles have been explored as alternative photocrosslinkers. Nevertheless, diazirines have emerged as the most practical and broadly adopted PAL tags, combining high stability in the dark, activation at biocompatible wavelengths, and a compact size that minimally perturbs binding interactions. Numerous reviews covering diazirines in PAL have been published in the last two decades^[^ ^4^, ^5^, ^6^, ^7^, ^8^, ^9^ ^]^ seemingly leading to the conclusion that PAL represents the only application of this class of compounds within the realm of chemical sciences (for a schematic depiction of diazirine activation in PAL, see Figure 1).

However, numerous exciting publications on the “non‐conventional” applications of diazirines have surfaced in the last two decades but were rather overlooked by the wider scientific community amidst the vastness of work published in the context of chemical biology. Intriguingly, the same properties that make this class of compounds irreplaceable in PAL can be exploited for many other applications in various chemical subdisciplines. For example, diazirine‐based carbene generation followed by insertion into certain covalent bonds is exploited in crosslinking of inert polymers, surface modifications of proteins, assembly of microelectronics, and many other areas of materials science. Additionally, their susceptibility to multiple activation modes, such as photo‐induced and voltage‐induced activation, makes them highly valuable assembly tools in biomaterials, where their reactivity can be tailored to specific in vitro and in vivo applications. Furthermore, diazirines serve as carbene and nitrogen sources in organic synthesis, simplifying known transformations and giving access to reactions that are hardly achievable otherwise. Last, ^15^N‐labeled diazirines are highly polarizing groups in NMR‐derived applications, where labeled biomolecules hold great potential as real‐time metabolic probes in MRT. While their general properties and synthetic methodologies utilizing diazirines have been extensively covered in recent reviews,^[^ ^10^, ^11^ ^]^ a comprehensive overview of applications in biological and medicinal sciences, materials chemistry, and NMR‐spectroscopy is missing. Therefore, the presented work intends to fill this gap by giving the reader an overview on diazirines in the aforementioned fields without putting a lengthy emphasis on substantially reviewed synthesis^[^ ^6^, ^10^, ^12^ ^]^ toward the diazirine building blocks. Ultimately, we aim at showing that this functionality is not just an appendage in chemical biology but a valuable tool in numerous areas of chemical synthesis and beyond.

## 2 Synthetic Methodologies Toward Diazirines and Their Properties

**Figure 2:**
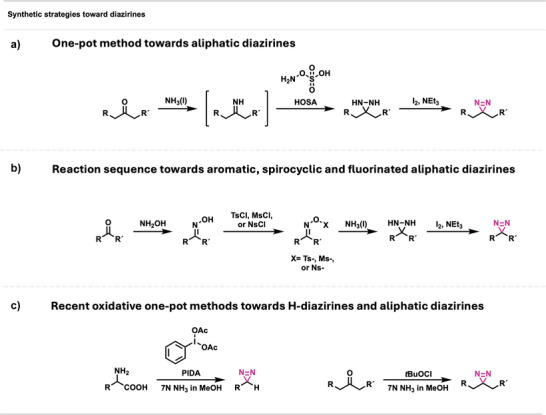
Synthetic methods toward diazirines. a) One‐pot method toward aliphatic diazirines using HOSA. b) Multi‐step method toward aromatic, spirocyclic, and fluorinated aliphatic diazirines. c) Recent, oxidative one‐pot methods toward *H‐*diazirines (left) and diversely functionalized aromatic and aliphatic diazirines (right).

Since synthetic methodologies toward diazirines have been extensively reviewed,^[^ ^5^, ^6^, ^8^, ^10^, ^12^ ^]^ only a brief overview of the most common strategies will be provided. Diazirines are usually synthesized from ketones via their respective imines or oximes. In the case of most aliphatic diazirines, a robust one‐pot method employs ammonia and hydroxylamine‐O‐sulfonic acid (HOSA) as a pre‐activated hydroxylamine source to directly generate the corresponding diaziridine intermediate (Figure 2a). In contrast, aromatic diazirines,^[^ ^13^, ^14^ ^]^ as well as certain spirocyclic^[^ ^15^ ^]^ and fluorinated aliphatic derivatives,^[^ ^16^ ^]^ are typically obtained through a multi‐step sequence in which the ketone is first converted into the oxime, which is then sulfonylated and treated with liquid ammonia to give the diaziridine. Subsequent oxidation of the diaziridine intermediates with silver oxide or iodine under basic conditions furnishes the desired diazirine (Figure 2b).^[^ ^5^ ^]^

Furthermore, some aromatic diazirines can also be accessed from imines,^[^ ^17^ ^]^ by reaction with liquid ammonia and HOSA and subsequent oxidation. Halodiazirines can be synthesized from amidines through oxidation with sodium hypochlorite or hypobromite,^[^ ^18^, ^19^ ^]^ resulting in the formation of chloro‐ or bromodiazirines, respectively. A more recent, one‐pot method involves the direct formation of *H*‐diazirines from unprotected amino acids via an oxidative decarboxylation and subsequent diazirine formation mediated by phenyliodine(III) diacetate (PIDA), conveniently carried out in 7 N methanolic ammonia (Figure 2c, left).^[^ ^20^ ^]^ Therein, PIDA is proposed to facilitate the oxidative decarboxylation of the amino acid, generating the corresponding imine, which subsequently reacts with an in situ generated iodonitrene to form a diaziridine. The diaziridine is then oxidized by a residual hypervalent iodine species to yield the corresponding diazirine. In a follow‐up publication, PIDA was replaced by *tert*‐butyl hypochlorite, which allowed the synthesis of various aliphatic and aromatic diazirines, starting from the respective ketones (Figure 2c, right).^[^ ^21^ ^]^

Diazirines exhibit a range of properties that render them highly attractive carbene precursors for diverse applications beyond PAL. In comparison to the closely related and widely used diazo functionality, diazirines offer greater stability^[^ ^22^ ^]^ and generally improved handling characteristics. Moreover, multiple modes of carbene generation from diazirines are well established and routinely employed, including photochemical,^[^ ^23^ ^]^ thermal,^[^ ^24^ ^]^ electrochemical,^[^ ^25^ ^]^ and metal‐catalyzed activation.^[^ ^26^ ^]^ Additionally, properties such as activation wavelength^[^ ^27^ ^]^ and carbene reactivity^[^ ^28^ ^]^ can be tuned by modifying substituents adjacent to the diazirine moiety, enabling the design of diazirines tailored to the specific requirements of a given application.

Although both aliphatic and aromatic diazirines are frequently employed, particularly in the context of PAL, a notable distinction in the reactivity of their derived carbenes must be emphasized. Carbenes generated from aryl diazirines—especially those bearing a trifluoromethyl substituent—readily insert into X─H bonds (e.g., O─H, S─H, N─H, and C─H) and generally do not undergo rapid intramolecular 1,2‐hydride shifts. In contrast, carbenes derived from aliphatic diazirines tend to undergo fast 1,2‐hydride shifts, yielding the corresponding alkenes, and are notoriously prone to oxidation by molecular oxygen to form ketones. While intermolecular reactions between these carbenes and highly polarized X─H bonds (such as O─H and S─H) can sometimes compete with 1,2‐hydride migration, insertions into the less reactive C─H bonds are typically not observed.^[^ ^13^, ^29^, ^30^ ^]^ A notable exception to this trend is found in carbenes derived from small‐ring spirocyclic diazirines, such as cyclobutyl diazirines.^[^ ^31^ ^]^

## 3 Applications of Diazirines in Medical and Biological Sciences Beyond PAL

In stark contrast to their dominant role in PAL probes, diazirines have seen isolated application in other areas of biological and medical sciences. This can be partly attributed to the short lifetime and rapid quenching of diazirine‐derived carbenes in aqueous media,^[^ ^32^, ^33^ ^]^ which represents the preferred environment for most biomolecules, including proteins. This subchapter will discuss the limited yet compelling applications of diazirines in biological research beyond PAL, including studies that either make sophisticated use of the reduced carbene stability in water or devise innovative strategies to overcome this limitation.

### 3.1 Protein Microenvironment‐Mapping via Dexter‐Energy Transfer

**Figure 3:**
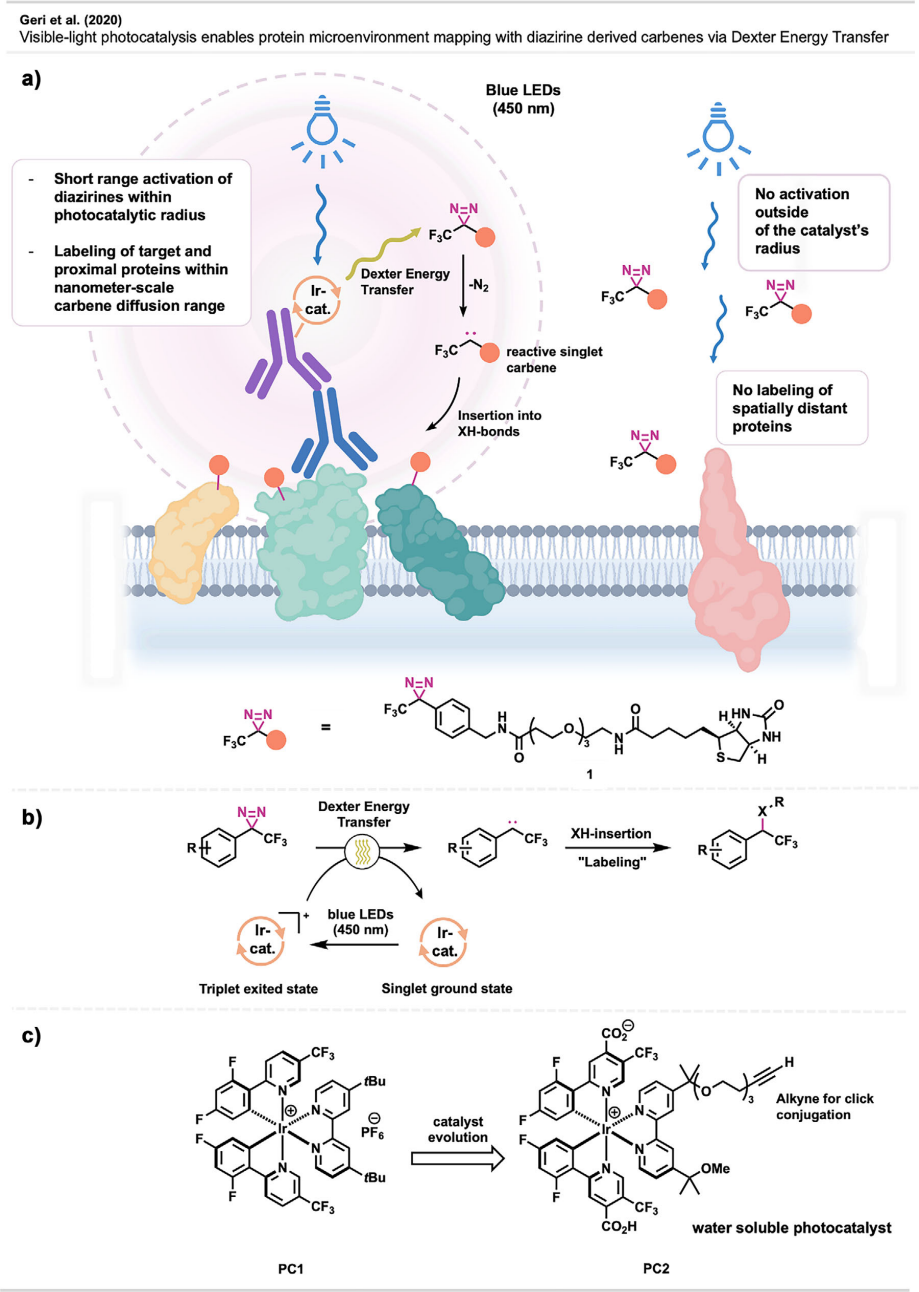
a) Graphical depiction of the basic principles of McMillan's microenvironment mapping. b) Principal depiction of Diazirine activation via Dexter‐energy transfer using an Ir‐photocatalyst. c) Structure of photocatalyst **PC1** and its evolution to a water‐soluble, clickable photocatalyst (**PC2**). Section a is redrawn and adapted from Lepage et al.,^[^ ^37^ ^]^ permission acquired via RightsLink. © The Authors of the original publication. Created with BioRender.com.

In an effort to map the microenvironment of proteins—i.e., the defined assemblies in which they are localized—MacMillan and coworkers^[^ ^34^ ^]^ employed diazirines to label proteins in the proximity of a designated protein of interest (POI) on cell surfaces. While conceptually related to PAL, this technique does not rely on the covalent attachment of a diazirine to a small molecule. Instead, it utilizes free trifluoromethyl aryl diazirines that are activated through short‐range Dexter energy transfer^[^ ^35^, ^36^ ^]^ mediated by an iridium photocatalyst positioned in close proximity to the POI. More specifically, a secondary antibody, targeting a primary antibody‐POI complex, is equipped with an iridium photocatalyst. Upon irradiation with blue light (450 nm), the catalyst is excited to a long‐lived triplet state (after a fast spin crossover from a singlet state), which in turn promotes free, biotinylated aryl diazirine **1** (Figure 3) into an excited triplet state via a short‐range (<1 mm)^[^ ^35^ ^]^ energy transfer. Following excitation, nitrogen extrusion from the diazirine generates a singlet carbene via intersystem crossing from the transient triplet state. The reactive carbene predominantly labels proteins located in close spatial proximity to the activation site (i.e., the POI), owing to the short lifetime of diazirine‐derived carbenes in aqueous media. While fast quenching of the carbene is usually considered a disadvantage in PAL, MacMillan's microenvironment mapping makes elegant use of this fact. (Figure 3a,b)

Methodologically, MacMillan and coworkers first sought to identify a suitable catalyst for diazirine activation, ultimately selecting catalyst **PC1** (Figure 3c). In control experiments, catalyst **PC1** exhibited high efficiency in activating aryl diazirines under mild conditions. Notably, activation occurred under 450 nm irradiation, where the extinction coefficient of the catalyst is five orders of magnitude greater than that of the diazirine. This indicates that non‐catalyzed diazirine activation at this wavelength is highly unlikely—a conclusion further supported by catalyst‐free control experiments. Next, the catalyst was structurally altered to yield water‐soluble compound **PC2** (Figure 3c), through attachment of free carboxylic acids and polyethylene glycols. Additionally, an alkyne moiety was introduced to enable biorthogonal conjugation of the catalyst to antibodies. Luckily, these alterations did not impact the catalyst's ability to activate biotinylated diazirine **1** (Figure 3a), which was shown to efficiently label bovine serum albumin (BSA), as demonstrated by Western blot and mass spectrometry. The photocatalyst was then attached to an azide‐functionalized secondary antibody and used for proximity mapping of several cell surface proteins (CD45, CD29, CD47, and programmed death ligand‐1), showing labeling of both known and unknown proximity‐associated proteins. In addition, several further experiments were performed that demonstrated the general applicability of the method, which will not be covered here due to their expansive nature.

In a follow‐up study,^[^ ^38^ ^]^ the same group aimed to adapt the method for mapping interactions between small molecules and proteins. This was achieved by conjugating a small molecule to an iridium photocatalyst, enabling proximity‐based photoconjugation of the respective small molecule's target proteins to biotinylated diazirine probe **1** (Figure 3a) via Dexter energy transfer. While conceptually similar to PAL, the small‐molecule binder itself is not activated, preventing its loss through side reactions with water and avoiding ineffective blocking of binding sites by spent probes, thereby significantly increasing labeling efficiency.

**Figure 4:**
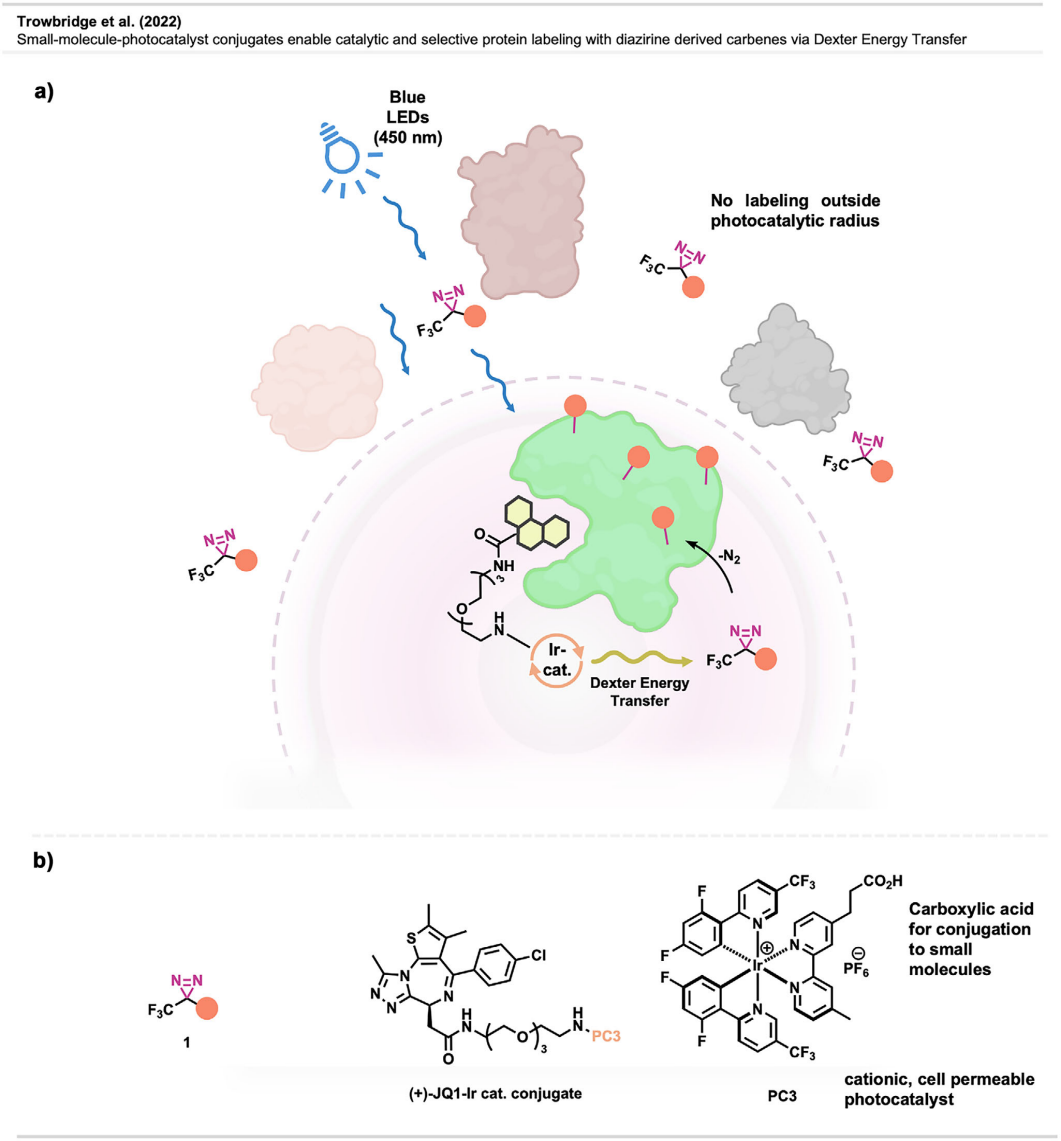
a) Schematic depiction of the principle behind McMillan's small molecule interactome mapping via Dexter energy transfer. b) Structures of the **(+)‐JQ1‐Ir catalyst conjugate** and the novel, cell‐permeable photocatalyst **PC3**. Created with Biorenden.com.

While the previously used catalyst **PC2** could not be employed due to its low cell permeability hailing from its neutral net charge and carboxylic acid residues, removal of the carboxylic acid residues yielded a cell‐permeable, functional cationic photocatalyst **PC3** (Figure 4b), further equipped with a carboxylic acid handle to enable amide‐based conjugation to small molecules. Next, (+)‐JQ1 (Figure 4b), a potent inhibitor of the BET family of bromodomain proteins, i.e., currently in clinical trials for various cancers, was conjugated to the catalyst and enabled the labeling of both known targets and off‐targets in live‐cell experiments. Labeled proteins were again identified by Western blot and proteomics. The method was further applied to other small molecules, such as dasatinib and paclitaxel, demonstrating its effectiveness in target and off‐target identification and its general applicability to a broad range of drug‐like compounds.

### 3.2 Covalent Attachment of Cofactors in Biocatalysis

Inspired by the classical photoaffinity labelling concept, the Mihovilovic and Ludwig groups^[^ ^39^ ^]^ picked up the fundamental principle of this technique to stabilize flavin‐dependent enzymes by covalently attaching the cofactor flavin‐adenine‐dinucleotide (FAD) to the protein's binding site. Although flavoenzymes with covalently attached FAD are known,^[^ ^40^ ^]^ the majority of flavin‐dependent enzymes comprise a non‐covalently bound FAD. The dissociation of the cofactor from the enzyme's binding site results in an immediate loss of activity and destabilizes the apoenzyme.^[^ ^41^ ^]^ Hence, the applicability of flavoenzymes is often limited by their notorious lability.

The synthesis of FAD analogs modified with a diazirine group to attach the cofactor covalently to the protein via a light stimulus was envisioned, thereby enabling the initiation of the covalent anchoring at will, allowing the correct incorporation of the cofactor, and preventing its dissociation.

**Figure 5:**
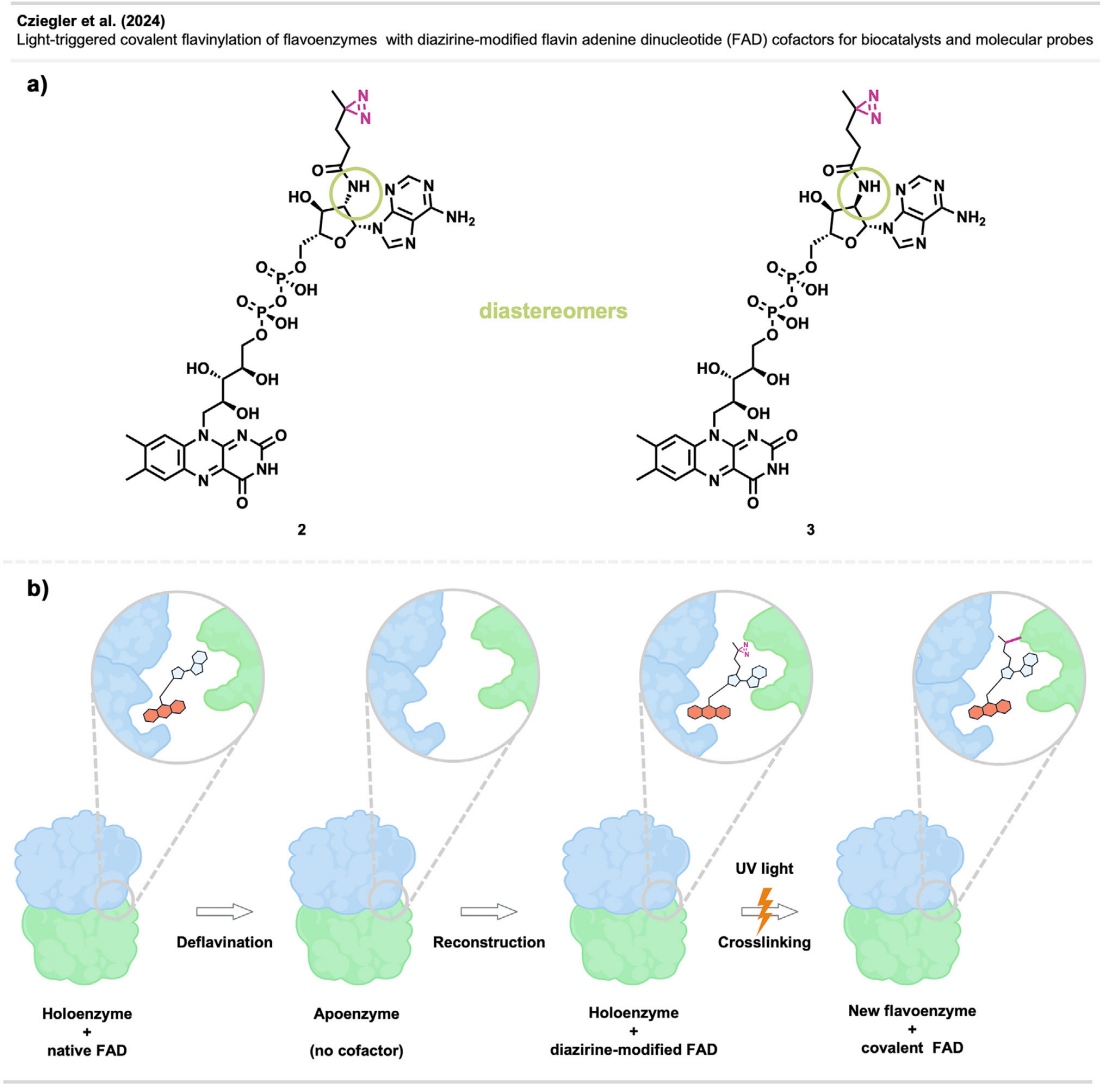
a) Diazirine‐functionalized artificial cofactors **2** and **3**. b) Overview of the covalent anchoring of the cofactor FAD to a flavoenzyme. The image was redrawn and adapted from C. Cziegler´s doctoral thesis.^[^ ^42^ ^]^ Created with BioRender.com.

To assess the feasibility of this approach in stabilizing FAD‐dependent enzymes, a set of six FAD analogs, modified with a diazirine functionality on the adenosine moiety of FAD, was synthesized. The deflavination of three model enzymes (glucose oxidase (GOx), cellobiose dehydrogenase (CDH), and cyclohexanone monooxygenase (CHMO)) and the subsequent reconstitution with the modified FAD analogs were attempted with all six analogs, showing that only reconstitution with compound **2** (Figure 5a) provided functional enzymes in all model enzymes. Furthermore, CHMO reconstituted with compound **3** (Figure 5a), compound **2**´s diastereomer, was also active.

Encouraged by these results, they further assessed the covalent tethering of compound **2** to the proteins’ structures upon UV light irradiation at 365 nm (Figure 5b). While the resulting covalent reconstitutes showed activity, albeit reduced (∼10%), as compared to the non‐covalent versions, they did not show altered thermodynamic and kinetic stability compared to holoenzymes carrying the natural cofactor. Nevertheless, the established methodology presents a new route for the stabilization and functionalization of cofactor‐dependent biocatalysts and could be expanded to cofactors beyond flavine.

### 3.3 Diazirines as Structural Features of Drug‐Like Molecules

**Figure 6:**
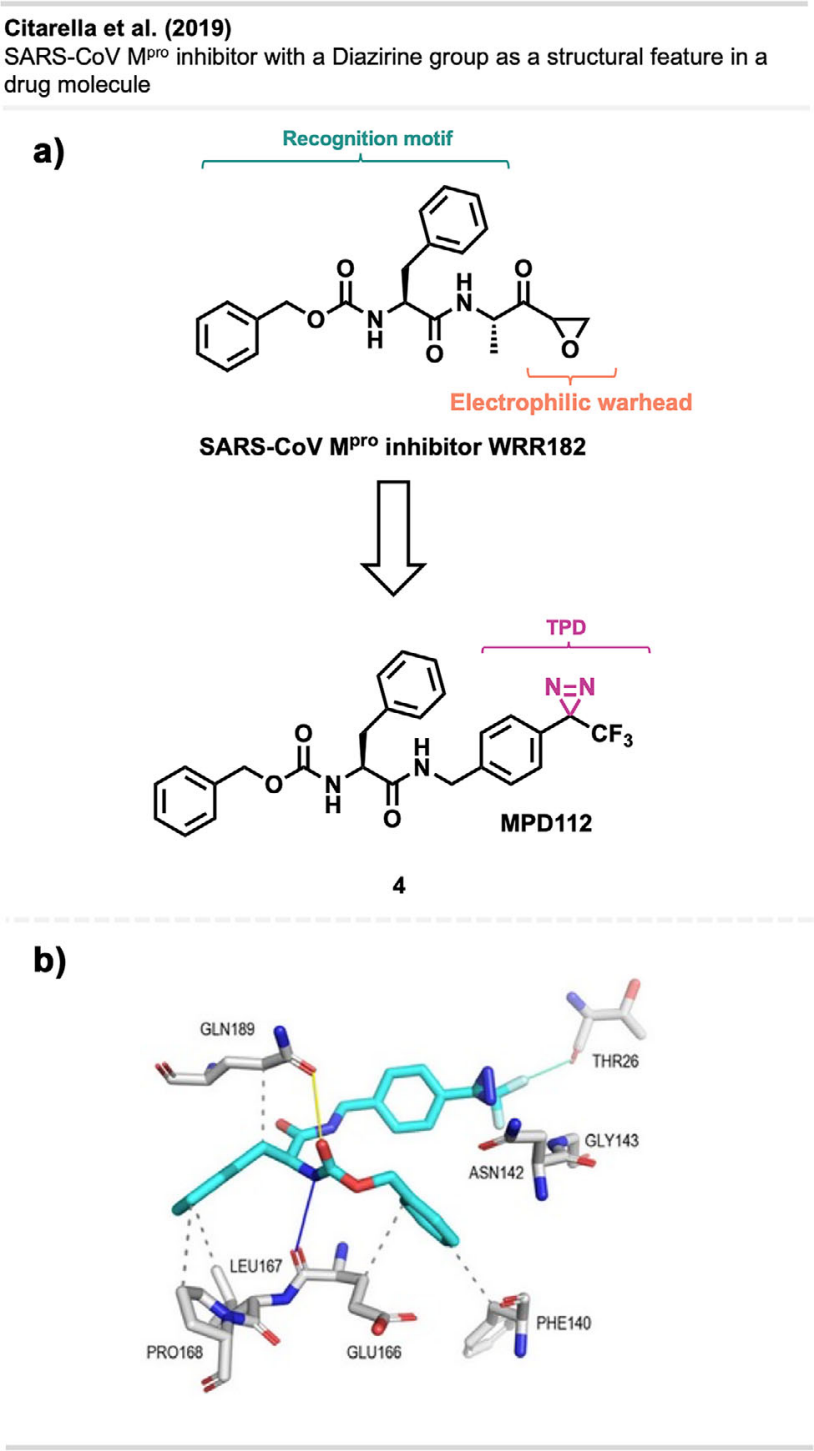
a) Representation of binder evolution from WRR182 to MPD112. b) Binding mode of MPD112 in SARS‐CoV‐Mpro´s active site, as determined by molecular docking. Section b is a direct reproduction from Citarella et al.,^[^ ^43^ ^]^ licensed under CC BY. Created with BioRender.com.

Despite its widespread use as a valuable crosslinking tool in chemical biology, the diazirine motif has not found significant application as a bioactive group in pharmaceutical compounds so far. Recently, Citarella et al.^[^ ^43^ ^]^ discovered MPD112 **(4)** (Figure 6a), a trifluoromethyl diazirine‐based compound that selectively inhibits the enzymatic activity of SARS‐CoV‐2 main protease (M^pro^). This main protease serves as a crucial enzyme in coronaviruses, playing a pivotal role in facilitating viral replication and transcription.^[^ ^44^ ^]^

The design of MPD112 **4** is based on a study by West et al.,^[^ ^29^ ^]^ in which the labeling preferences of trifluoromethyl diazirines were investigated. It was shown that cysteine was the most reactive amino acid toward 3‐trifluoromethyl‐3‐phenyldiazirines (TPDs), reacting with the electrophilic diazirine carbon. Hence, the electrophilic warhead of WRR182 (Figure 6a), a known mechanism‐based inhibitor of SARS‐CoV‐2 M^pro^, was replaced by the TPD motif. The compound showed low‐micromolar inhibition of recombinant SARS‐CoV‐2 M^pro^ in vitro, and computational studies suggested electrostatic interactions of the electrophilic diazirine carbon with the nucleophilic thiol moiety of Cys145. Moreover, hydrophobic interactions of the phenyl groups with apolar amino acid residues and hydrogen bonding of the carbamate group to the backbone oxygen of a glutamic acid residue (Glu166) contribute to the affinity of MDP112 for SARS‐CoV M^pro^ ´s active site (Figure 6b). This initial finding introduces a novel biological application of trifluoromethyl diazirine derivatives as inhibitors of SARS‐CoV‐2 M^pro^ and shows the potential of diazirine moieties as new functional groups in drug discovery.

### 3.4 Protein Modification by Photo Conjugation

**Figure 7:**
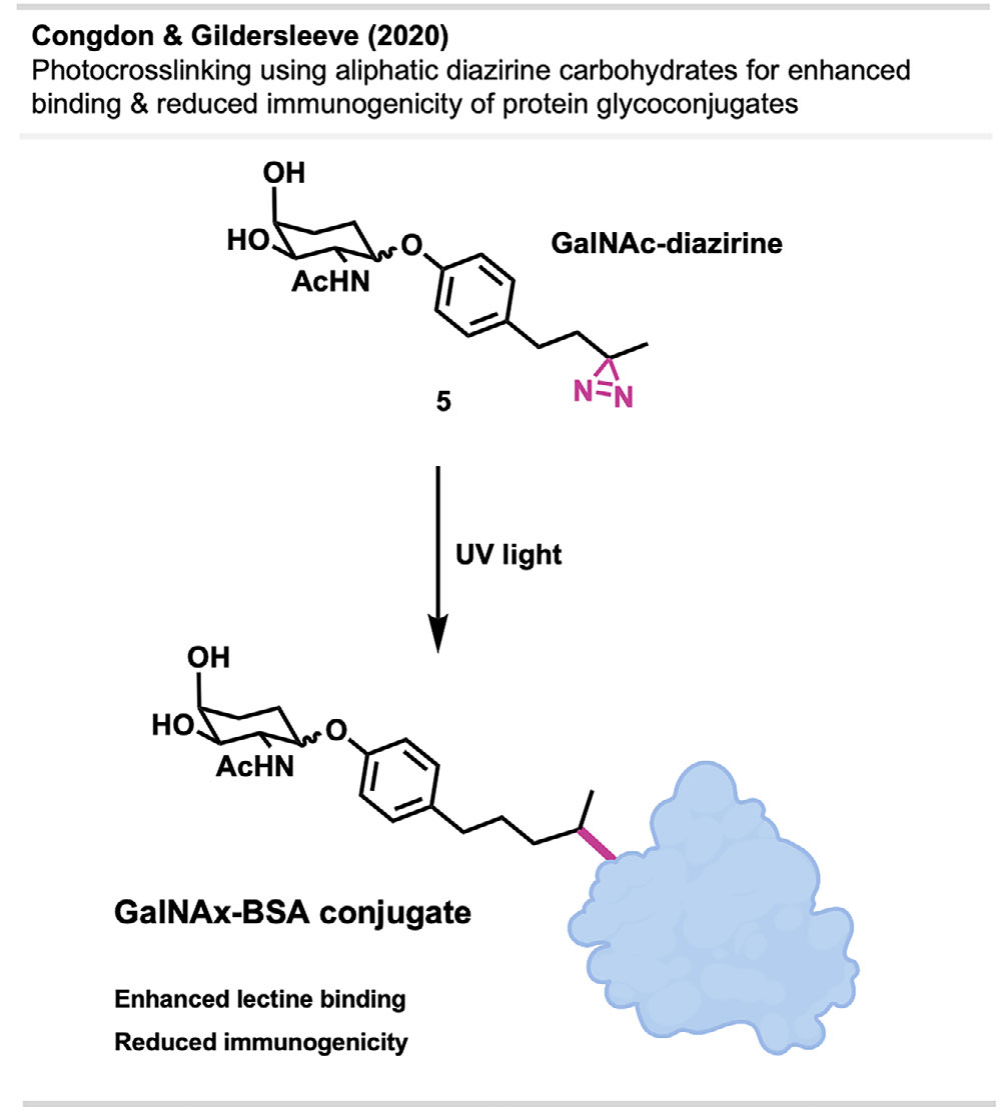
Schematic representation of carbohydrate–protein conjugation. Created with BioRender.com.

Another alternative biological application of diazirines has been explored by Congdon and Gildersleeve.^[^ ^45^ ^]^ They utilized a solid‐state photoactivation method for aliphatic diazirines to create carbohydrate–protein conjugates involving BSA and Human Serum Albumin (HSA) (Figure 7).

While various conjugation methods exist, such as amide formation, Michael addition with maleimide‐modified biomolecules, or utilizing isothiocyanates as well as azide‐alkyne‐based click chemistries, some protein targets lack necessary residues or have competing reactive moieties at critical locations. The use of a non‐specific photophore allows potential modification at any surface‐exposed area without causing severe alterations to the protein, which could lead to destabilization or denaturation. This approach is not restricted to specific residues, and it avoids changes in isoelectric points and net charge, common issues with amide bond formations involving lysine residues.

To address the challenge of low yield in photoactivated biological conjugation—arising from the short lifespan of carbenes in aqueous media—the authors introduced a solid‐state method to enhance conjugation efficiency in glycoconjugate formation. This was accomplished by irradiating a solvent‐free mixture of an N‐acetyl galactose (GalNAc) derivative carrying an aliphatic diazirine and BSA, prepared through lyophilization of a solution containing both coupling partners (Figure 7). This innovative photo conjugation methodology resulted in multivalent carbohydrate–protein conjugates with unchanged protein charge and intact secondary structure. It yielded up to a 100‐fold improvement in binding to lectins (carbohydrate‐binding proteins) compared to lysine‐*N*‐hydroxysuccinimide (NHS)‐based amide‐linked carbohydrate conjugates. Moreover, it led to diminished immunogenicity in mice.

### 3.5 Micelle‐Based Drug Delivery Systems

**Figure 8:**
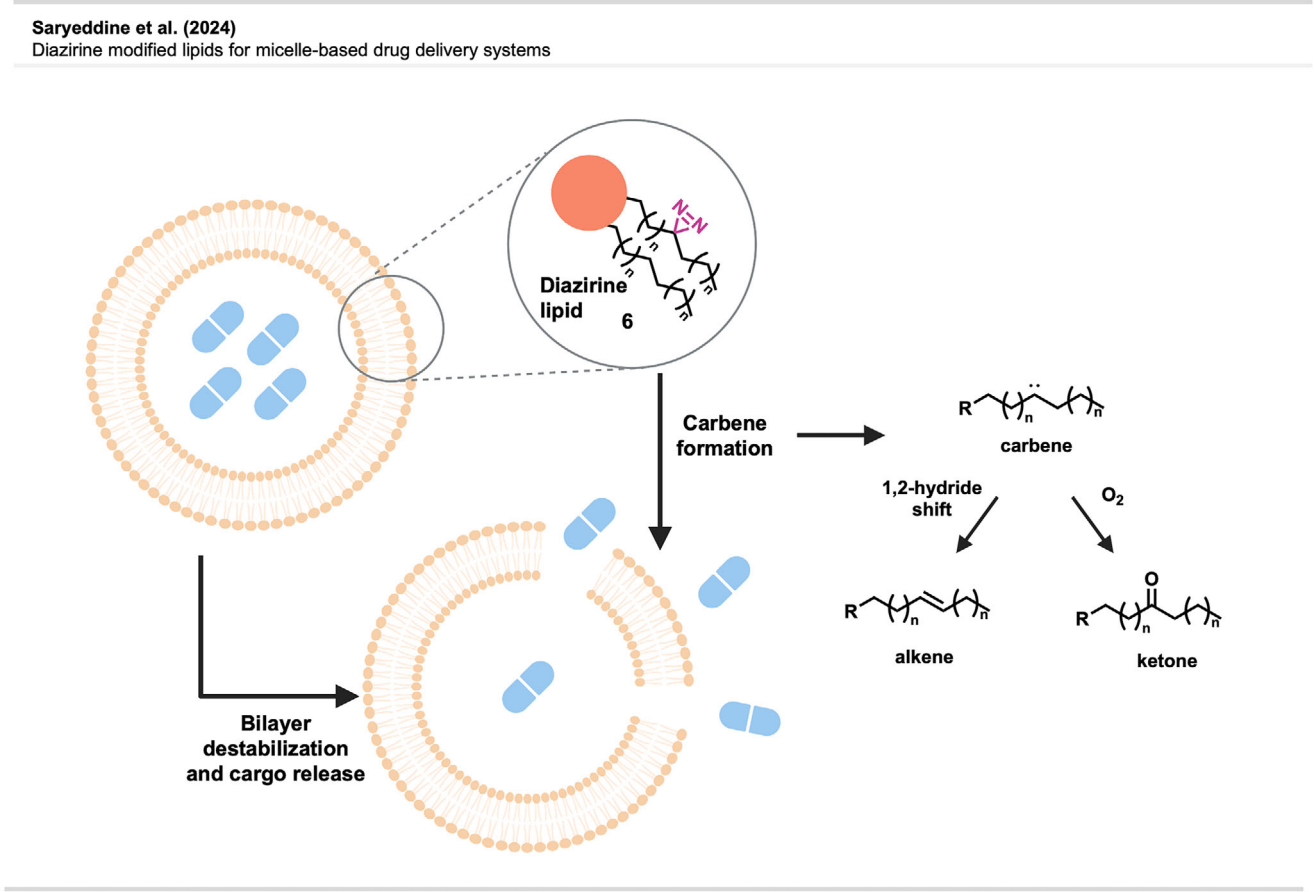
Schematic depiction of bilayer disruption after irradiation and observed follow‐up reactions upon carbene formation. Created with Biorender.com.

To design a light‐responsive drug delivery system for controlled cargo release, Badarau^[^ ^46^ ^]^ and coworkers modified an existing liposome‐based system, known as 1,2‐dimyristoyl‐*sn*‐glycero‐3‐phosphocholine (DMPC), by incorporating diazirine moieties into the fatty acid chains. While unmodified DMPC is not able to retain cargo due to the inherent fluidity of the formed bilayers, it was found that modifications of the *sn*‐2‐fatty acid by incorporation of a diazirine moiety at the C5‐position led to stiffer membranes and an increase in stability of the formed bilayers. This enhancement was evidenced by solid‐state NMR and cryoEM methods. However, light activation and subsequent reactions of the corresponding carbenes result in highly permeable vesicles due to morphological defects, unable to retain the cargo, leading to a controlled release of the payload. Formulations of DMPC‐diazirine carrying the hydrophilic fluorophore calcein as model cargo were prepared, and it was shown that, in solution, the liposomes were able to retain the cargo for at least 30 days. Irradiation at 365 nm, however, triggered cargo release in a magnitude dependent on irradiation time and intensity. Analysis of subsequently formed products by HPLC showed that the two main reaction products were unsaturated phospholipids (Figure 8) due to a carbene‐induced 1,2‐hydride shift, an inherent trait of alkyl diazirine‐derived carbenes (see Chapter **2**), and the corresponding ketones (Figure 8) due to reaction of the carbene with molecular oxygen. Both functionalities are known to be membrane softeners, leading to increased permeability and therefore a decrease in cargo‐retention capabilities. Furthermore, no possible crosslinks between fatty‐acid chains were detected. Hence, the authors hypothesized that crosslinking only plays a marginal role in the triggered cargo release.

## 4 Diazirines in Materials Science

In the dynamic field of materials science, the utilization of diazirines is currently emerging as a fascinating avenue, showcasing their versatility and impact across various domains. This chapter delves into the multifaceted applications of diazirines in polymer science, applications in electronic devices, surface modifications, and bioadhesives, shedding light on their transformative role in enhancing material properties and functionalities.

### 4.1 Diazirines in Polymer Crosslinking

Although carbene‐generating reagents have been used in chemical biology for decades, until recently, only a few studies have used diazirines in polymer science, and they mainly focused on highly functionalized materials or substrates with weak C─H bonds.

**Figure 9:**
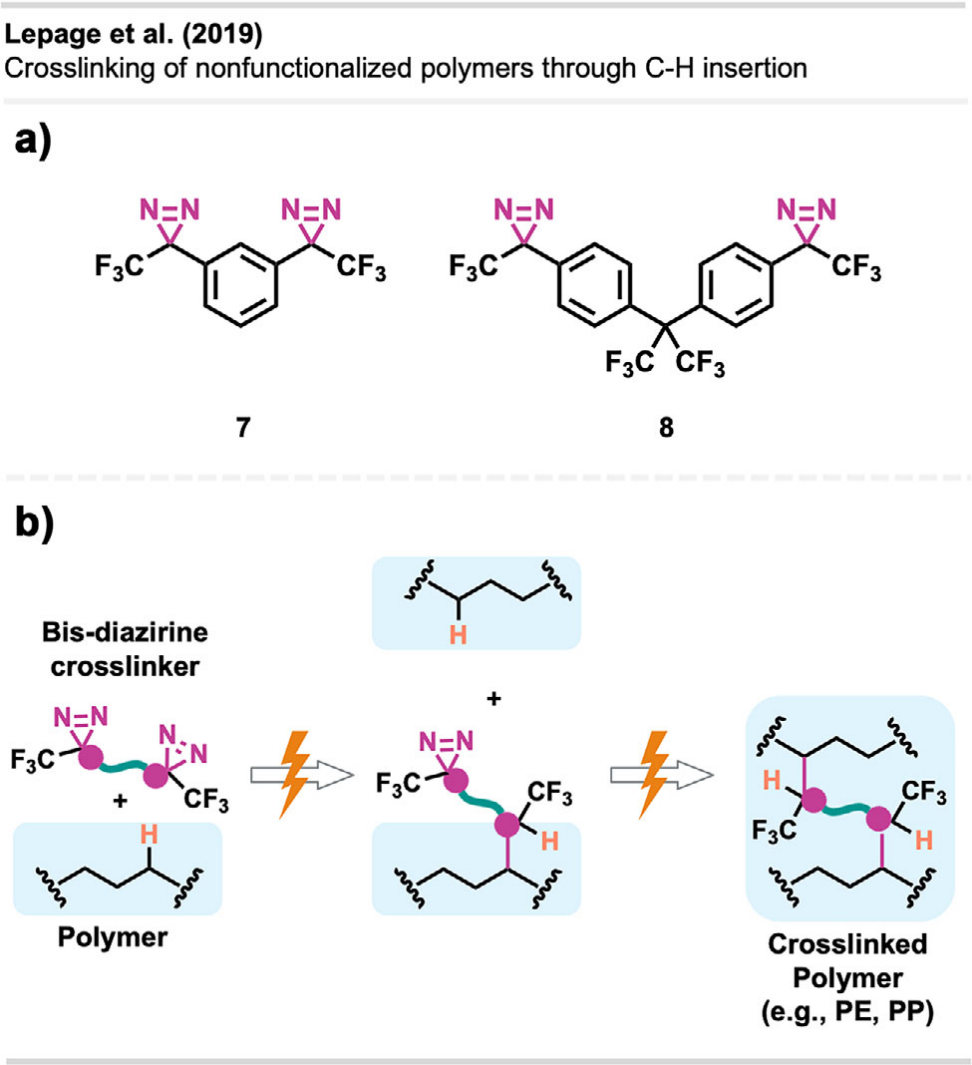
a) Bis‐diazirine crosslinkers **7** and **8**. b) Depiction of the crosslinking mechanism with polymers via C─H insertion. Section b is redrawn and adapted from LePage et al.,^[^ ^37^ ^]^ permission acquired via RightsLink. © The Authors of the original publication. Created with BioRender.com.

The use of diazirines to modify polymers was first reported by Blencowe and Hayes^[^ ^47^, ^48^, ^49^, ^50^ ^]^ in the mid‐to‐late 2000s. In 2006, they demonstrated the successful incorporation of several diazirine‐functionalized molecules onto the backbone of nylon 6,6^[^ ^50^ ^]^ and other polymers via carbene insertion.^[^ ^47^, ^49^ ^]^ Furthermore, bis‐diazirine **7** (Figure 9a) and other tri‐ and tetravalent diazirine linkers were used for the functionalization and crosslinking of poly(ethylene oxide)‐based gels and PEG block copolymers.^[^ ^48^ ^]^

It took more than a decade after the first report of bis‐diazirine‐based crosslinking for their full potential to gain broader recognition. Featuring reactive groups at both ends, these crosslinkers enable covalent bonding between polymer chains, even across unreactive or chemically distinct materials (Figure 9b).

In recent years, Wulff and coworkers have made significant advances in diazirine‐based polymer crosslinking. Wulff has thoroughly detailed his substantial contributions to the field in a recent account.^[^ ^51^ ^]^

Lepage et al.^[^ ^37^ ^]^ delivered a hallmark contribution to the field by introducing a universally applicable bis‐diazirine crosslinker **8** (Figure 9) that covalently binds virtually any aliphatic polymer via C─H insertion, in contrast to other crosslinking strategies that rely on functionalized polymers. In their design, they omitted labile heteroatomic bonds as well as any aliphatic C─H bonds that could participate in self‐reactions. Another design goal was diminishing safety hazards through increased molecular weight, reducing volatility and explosion risk compared to analogous phenyl‐ and pyridine bis‐diazirines. The crosslinkers were activated by long‐wave ultraviolet irradiation at 350 nm and heating at 110 °C–140 °C.

Bis‐diazirines enabled efficient crosslinking of diverse polymers, including polyethylene, polypropylene, polyisoprene, polystyrene, polycaprolactone (PCL), polyvinyl alcohol, and polydimethylsiloxane (PDMS). Furthermore, bis‐diazirine **8** enhanced adhesion and mechanical strength in high‐density polyethylene (HDPE) and ultrahigh‐molecular‐weight polyethylene (UHMWPE), demonstrating its potential for industrial applications. In a safety assessment, Baran et al.^[^ ^52^ ^]^ evaluated bis‐diazirine crosslinker **8**, which was later commercialized as BondLynx, using a comprehensive array of in vitro tests. The compound was found to be non‐mutagenic, non‐phototoxic, and non‐corrosive or irritating to skin and eyes. These results support a favorable safety profile for diazirine‐based crosslinkers and adhesives in material applications.

**Figure 10:**
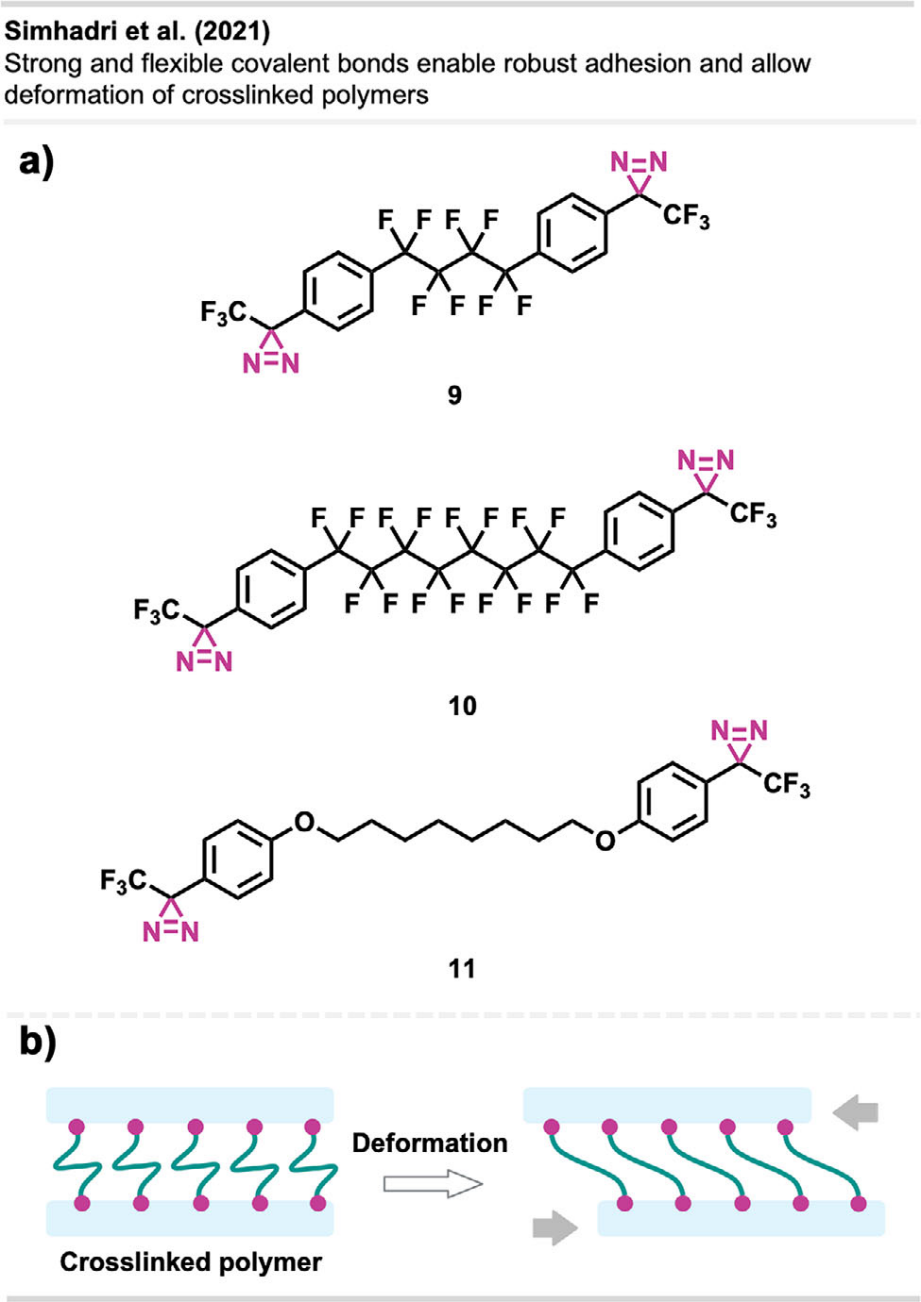
a) Flexible polyfluorinated crosslinker **9**, **10** & **11** and electronically optimized bis‐diazirine with an electron‐donating linker. b) Schematic depiction of flexible crosslinking of polymers with polyfluorinated diazirenes. Section b was redrawn and adapted from Simhadri et al.,^[^ ^53^ ^]^ licensed under CC BY. © 2021 Royal Society of Chemistry. Created with BioRender.com.

In follow‐up studies, Wulff and coworkers further demonstrated the potential of crosslinking‐based adhesion with a second generation of bis‐diazirines **9** and **10** bearing polyfluorinated chains (Figure 10a). These flexible linkers allow the deformation of the adhered materials without mechanical failure (Figure 10b). Although traditional adhesives like cyanoacrylates (“super glue”) also allow plastic deformation and strong bond formation with several surfaces, they still suffer from weak adhesion to low surface energy materials (e.g., PE, PP). The crosslinked samples achieved far better results than the “super glue” controls in the adhesion testing experiments. Thus, diazirine‐based adhesives are promising alternatives to traditional products.^[^ ^53^ ^]^

Furthermore, electronically optimized bis‐diazirine crosslinkers have been developed by the same group. It was found that electron‐rich aryl bis‐diazirines outperformed all counterparts from earlier generations at crosslinking cyclohexane, used as a model substitute for polyethylene. While bis‐diazirines **9** and **10** showed C─H insertion yields of only 6% to 7%, **11** (Figure 10a) showed a 10‐fold better crosslinking efficiency.^[^ ^54^ ^]^ These findings align with recent Hammett studies of mono‐diazirines that demonstrated an increased stabilization of the intermediate singlet‐carbene by electron‐rich substituents and hence, a preferred C─H insertion over radical mechanisms. Despite the long aliphatic tether, no intramolecular side reactions were observed.^[^ ^27^ ^]^

**Figure 11:**
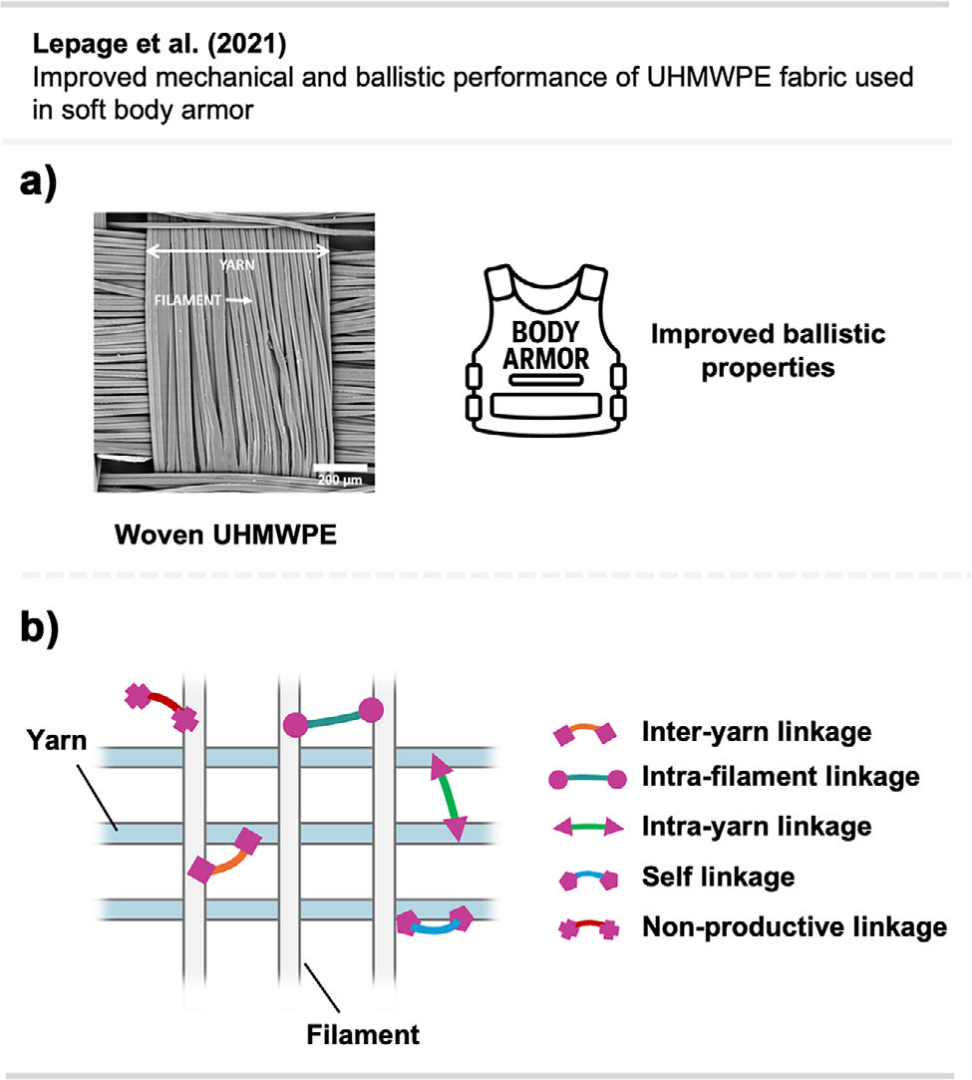
a) Scanning electron micrograph of UHMWPE fabric illustrating the microstructure at a yarn intersection (the white scale bar represents 200 µm). b) Schematic depiction of crosslinking of woven UHMWPE for reinforcement of ballistic fabric. The scanning electron micrograph in Section a is a direct reproduction from Lepage et al.,^[^ ^55^ ^]^ permission acquired via RightsLink. © American Chemical Society. Created with BioRender.com.

The adhesive strength of **9** and **10** was also demonstrated by topical crosslinking of woven UHMWPE fabric used in soft body armor and ballistic clothing (Figure 11a). Thereby, the fabric's stiffness and resistance to tearing and perforation could be drastically increased by interyarn and intrayarn linking (Figure 11b).^[^ ^55^ ^]^

In a recent study, Mahbod et al.^[^ ^56^ ^]^ demonstrated that bis‐diazirine crosslinking of UHMWPE fabrics, combined with a shear‐thickening fluid, markedly enhanced puncture resistance and impact absorption. Thermal activation enabled covalent bonding to the polymer backbone, resulting in a 92% increase in puncture resistance and enhanced strain rate‐dependent behavior across various impact conditions, highlighting a promising strategy for lightweight, high‐performance armor and protective composites.

It has been demonstrated that bis‐diazirines facilitate the fabrication of thermosets (i.e., an insoluble polymer permanently crosslinked through an irreversible curing reaction), endowing them with heightened strength and superior material properties. Nevertheless, the challenge in reprocessing and recycling these thermosets lies in the covalent interchain crosslinks contributing to their appeal.

**Figure 12:**
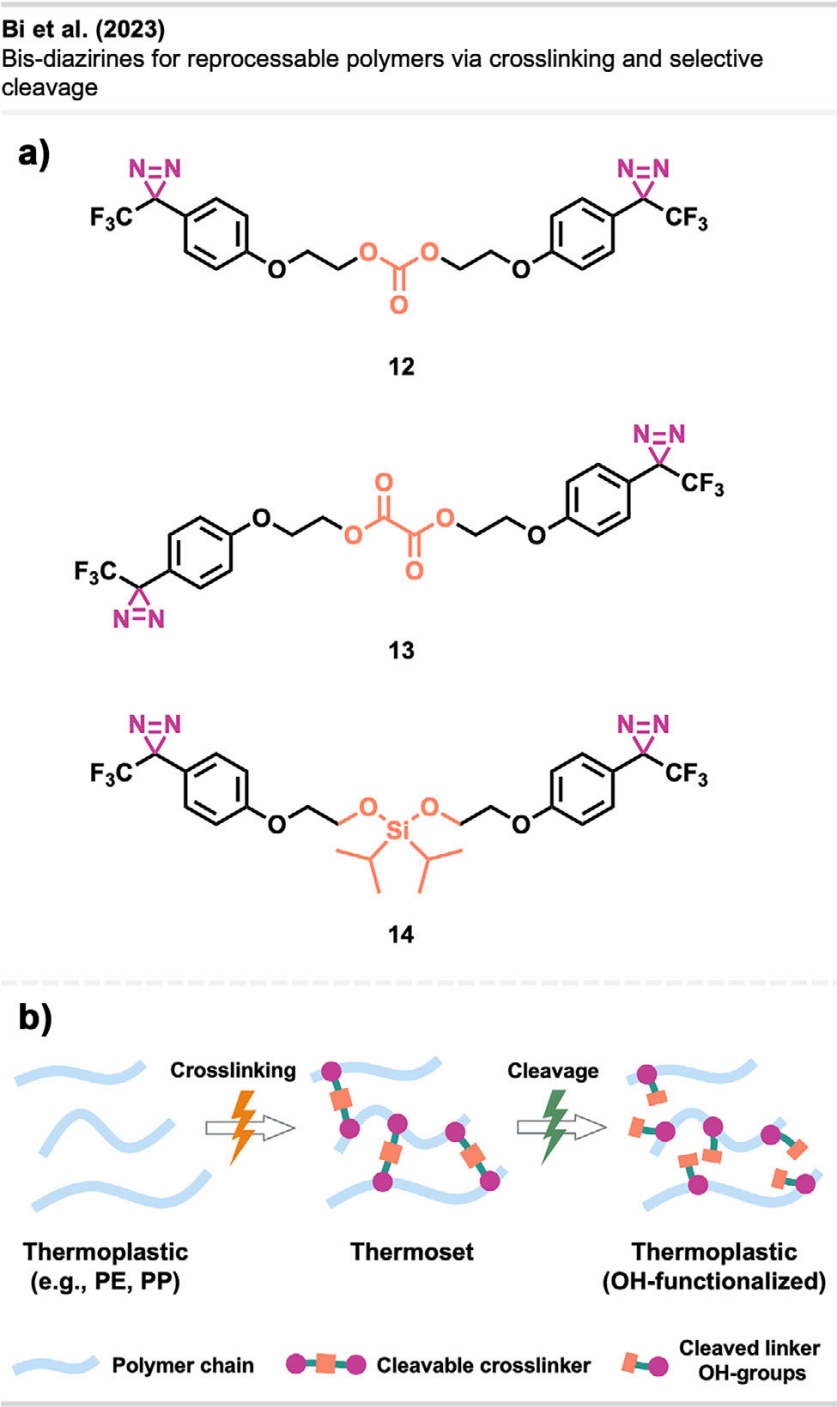
a) Crosslinker **12**–**14** with chemically cleavable functional groups. b) Thermoplastics can be crosslinked to form strong thermosets bearing a cleavable linker to enable thermoplastic regeneration and potential re‐functionalization. Section b was redrawn and adapted from Bi et al.,^[^ ^57^ ^]^ licensed under CC BY. © 2023 The Authors of the original publication. Created with BioRender.com.

A study by Bi et al.^[^ ^57^ ^]^ introduced a novel approach equipping bis‐diazirine linkers with chemically cleavable functional groups, including carbonate **12**, oxalate **13**, and silyl ether functionalities **14** (Figure 12a). These groups may enable reprocessing of crosslinked low‐functionality commodity polymers through strong covalent bonds that, at the same time, can be selectively cleaved by chemical stimuli (e.g., acid, base, or fluoride).

The crosslinkers demonstrated excellent C─H insertion efficiency, transforming thermoplastics into high‐performance thermosets with tunable modulus and robust mechanical properties. Crucially, the crosslinked networks could be selectively cleaved to regenerate thermoplastic materials or allow re‐functionalization, offering a circular route for polymer processing and recycling (Figure 12b). The method was also successful in mixed polyolefin systems, addressing a key challenge in plastic waste management.

This study demonstrated crosslinking and de‐crosslinking of common polymers, including isotactic polypropylene (iPP), PDMS, and blends of typically incompatible materials like iPP and PE. De‐crosslinked products retained hydroxyl groups, enabling re‐crosslinking and further modification, opening new avenues for polymer upcycling and advanced material design.

**Figure 13:**
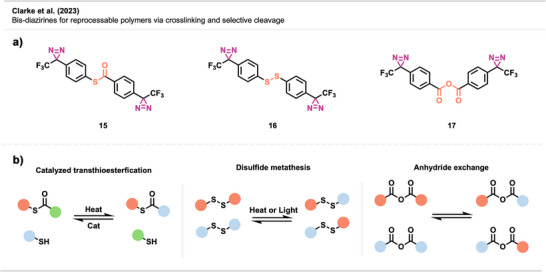
a) Crosslinkers **15**–**17** with chemically cleavable functional groups. b) The crosslinkers contain thioester, disulfide, and anhydride moieties that can undergo catalyzed transthiolate‐thioesterfication, disulfide metathesis, and anhydride exchange reactions, respectively. The linker concept enables compatibility, increases the performance of crosslinked thermosets, and allows on‐demand cleavage for recyclability.

In a similar approach, Clarke et al.^[^ ^58^ ^]^ introduced novel bis‐diazirine crosslinkers **15**–**17 (**Figure 13a) to compatibilize (i.e., merge otherwise incompatible polymers into a stable and uniform blend) various classes of immiscible polymer mixtures, creating reprocessable and recyclable thermosets. For that, they designed crosslinkers containing thioester, disulfide, and anhydride moieties that can capitalize on catalyzed thiolate‐thioester, disulfide metathesis, and anhydride exchange reactions, respectively (Figure 13b). This dynamic system enables selective bond exchange for recycling and facilitates compatibilization of otherwise immiscible polymer blends by forming grafted multiblock copolymer (gMBCP)‐like architectures that bridge polar and apolar domains.

To demonstrate reprocessability, the authors performed model reactions comparing carbene insertion into cyclohexane and methyl methoxyacetate, mimicking apolar polyolefins (e.g., PE) and polar bioplastics (e.g., PLLA or P3HB), respectively. The results revealed insertion and dynamic exchange, showcasing how such systems enable reversible bonding and compatibilization in real polymer blends.

The authors showed crosslinking of polyolefins such as LDPE, HDPE, iPP, and UHMWPE, yielding materials with improved mechanical strength and creep resistance. Their approach also enabled compatibilization in complex blends, including a ternary LDPE‐iPP‐PLLA system and real‐world plastic waste. Additionally, **16** proved effective in compatibilizing mixed plastics with dyes and additives, as demonstrated in mechanically shredded flakes of an LDPE bag and a PLLA cup. Such approaches offer promising strategies for recycling mixed plastics and recovering material value.

**Figure 14:**
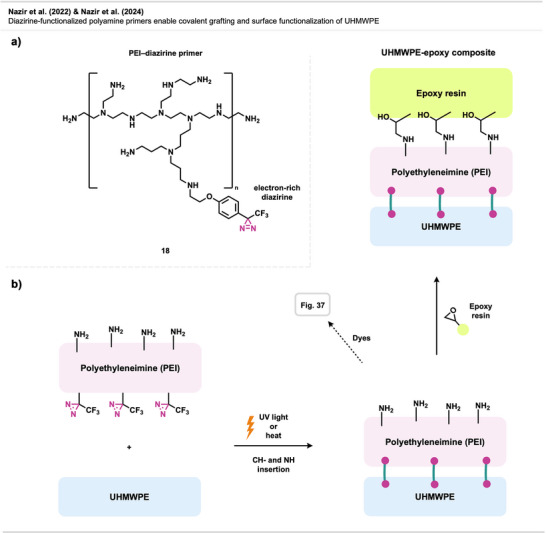
Diazirine‐polyamine conjugate **18** was used to covalently modify polyethylene fibers, enabling the fabrication of polyethylene‐epoxy composite materials.

Nazir et al.^[^ ^59^ ^]^ introduced a diazirine‐based strategy to improve the interfacial adhesion between UHMWPE and epoxy matrices by using diazirine‐functionalized polyamine primers (Figure 14). Upon thermal or photochemical activation, these primers generate carbenes that insert into C─H bonds on the UHMWPE surface while presenting amine groups for covalent coupling with epoxy resins. Polyethylenimine (PEI)‐based primers outperformed small tetraamine and dendrimeric analogues, yielding composites with high interfacial shear strength and cohesive failure.

Building on this concept, the same research group^[^ ^60^ ^]^ later applied PEI‐diazirine conjugates to the surface modification and dyeing of UHMWPE fabrics (see Figure 37), with the electron‐rich PEI‐diazirine conjugate **18** showing the highest performance and achieving robust covalent grafting without oxidative pretreatment.

The electron‐rich diazirine primers exhibited higher carbene insertion efficiency and stronger surface bonding than electron‐neutral analogues. The reaction proceeds through C─H insertion into polyethylene and N─H insertion or self‐crosslinking within the PEI network. The N─H insertion preference of electron‐rich diazirines was demonstrated through model photolysis experiments and spectroscopic analysis, revealing singlet‐carbene‐mediated self‐crosslinking within the polyamine coating, which forms a durable, reactive layer capable of binding epoxy matrices, dyes, or other functional molecules. Together, these studies establish diazirine‐mediated polyamine grafting as a versatile, non‐oxidative method for functionalizing inert polyolefin materials with strong mechanical and chemical robustness.

**Figure 15:**
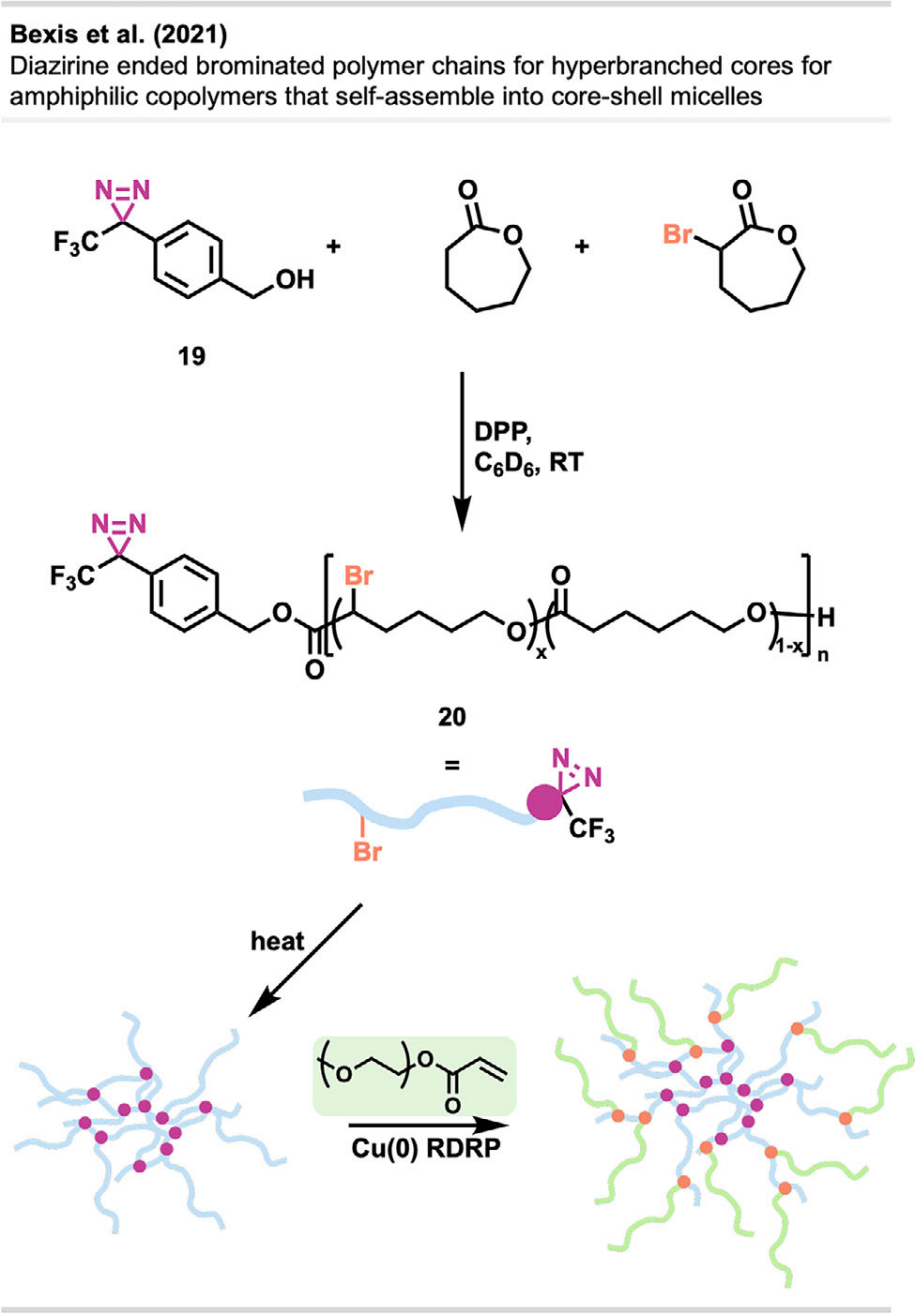
Copolymerization of ε‐caprolactone and α‐bromo‐ε‐caprolactone catalyzed by DPP and initiated by **19**, yields α‐diazirine copolymers. Thermal activation of the diazirine moieties generates carbenes, inducing hyperbranching of the linear polymer precursors **20**. The subsequent application of Cu(0)‐mediated RDRP allows for “grafting‐from” the PCL‐based backbone, affording amphiphilic polymer architectures. The figure was redrawn and adapted from Bexis et al.,^[^ ^61^ ^]^ licensed under CC BY. © 2021 Royal Society of Chemistry. Created with BioRender.com.

Bexis et al.^[^ ^61^ ^]^ showed the formation of hyperbranched, biodegradable PCL‐based polymer networks used for “grafting‐from” polymerizations. These amphiphilic copolymers self‐assemble into spherical core–shell micelles, making them attractive materials for biological applications. ε‐Caprolactone and α‐bromo‐ε‐caprolactone were first copolymerized with **19** as an initiator in a ring‐opening polymerization catalyzed by diphenyl phosphate (DPP) (Figure 15). This yields polyester chains containing a terminal diazirine moiety **20**. Thermal activation releases the reactive carbene species to initiate branching of the polymer chains. The bromine handle is then used to graft hydrophilic PEG chains by Cu(0)—reversible deactivation radical polymerization (RDRP) techniques.

**Figure 16:**
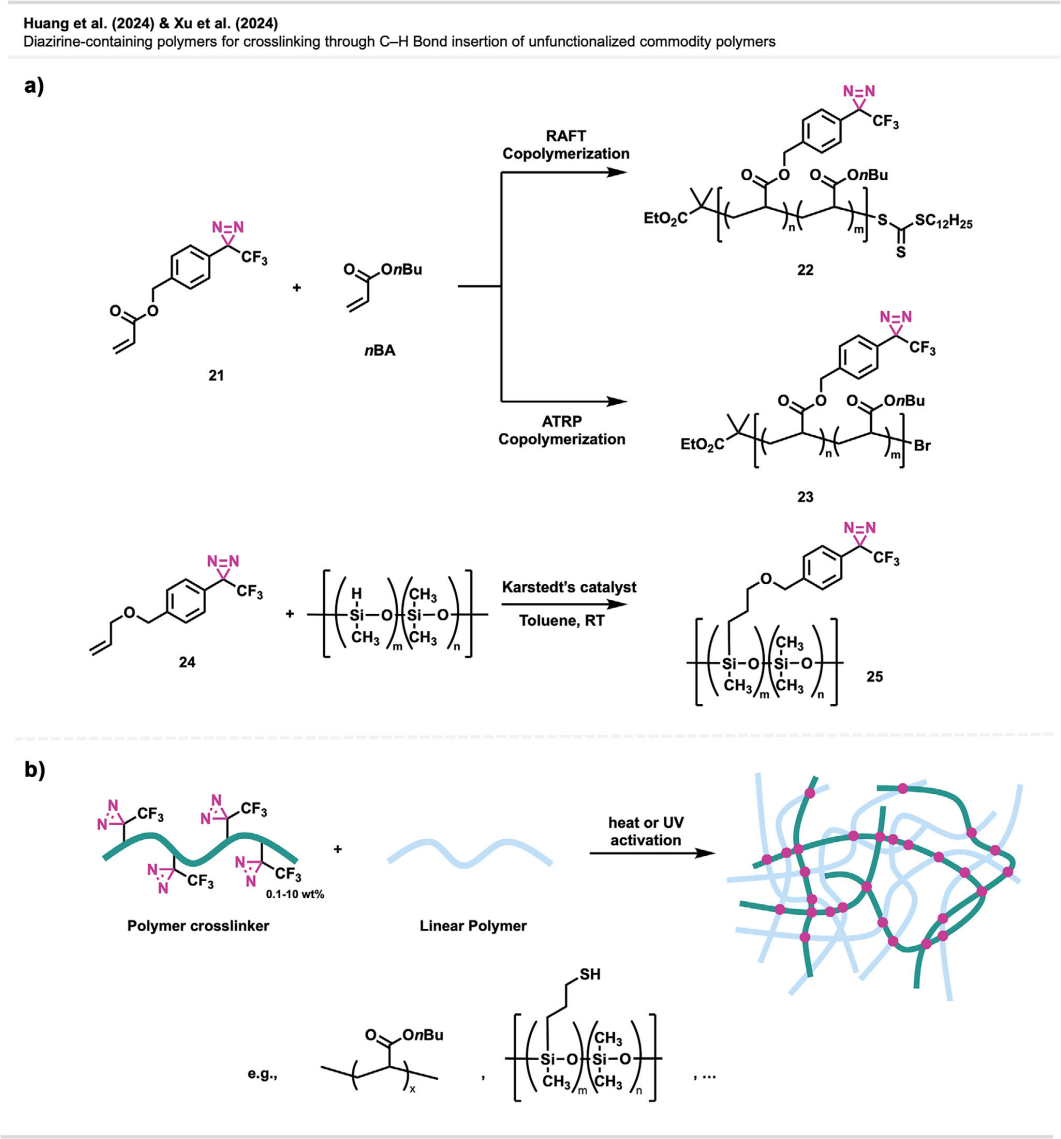
a) Formation of diazirine‐containing polymers. b) The diazirine polymer crosslinker is reacted with linear polymers to form crosslinked networks. Section b was redrawn and adapted from Xu et al.,^[^ ^63^ ^]^ licensed under CC BY. © 2024 The Authors of the original publication. Created with BioRender.com.

Huang et al.^[^ ^62^ ^]^ developed a versatile method for synthesizing diazirine‐containing polymers, employing both reversible addition‐fragmentation chain transfer (RAFT) and atom transfer radical polymerization (ATRP) techniques. For instance, they introduced diazirine‐functionalized acrylate **21** and methacrylate monomers, which were copolymerized with common vinyl monomers such as poly(*n*‐butyl acrylate) (Figure 16a). The resulting polymers **22** and **23** exhibited precise molecular control and retained full diazirine integrity. Upon activation by either heat (∼100 °C) or UV light (370 nm), efficient carbene‐mediated C─H insertion crosslinking was observed. Gel fractions (i.e., the portion of a polymer network insoluble after crosslinking) above 95% demonstrated excellent crosslinking efficiency. Blending studies further confirmed that these diazirine copolymers could effectively crosslink commodity polymers like poly(nBA), broadening their application in adhesives and coatings.

**Figure 17:**
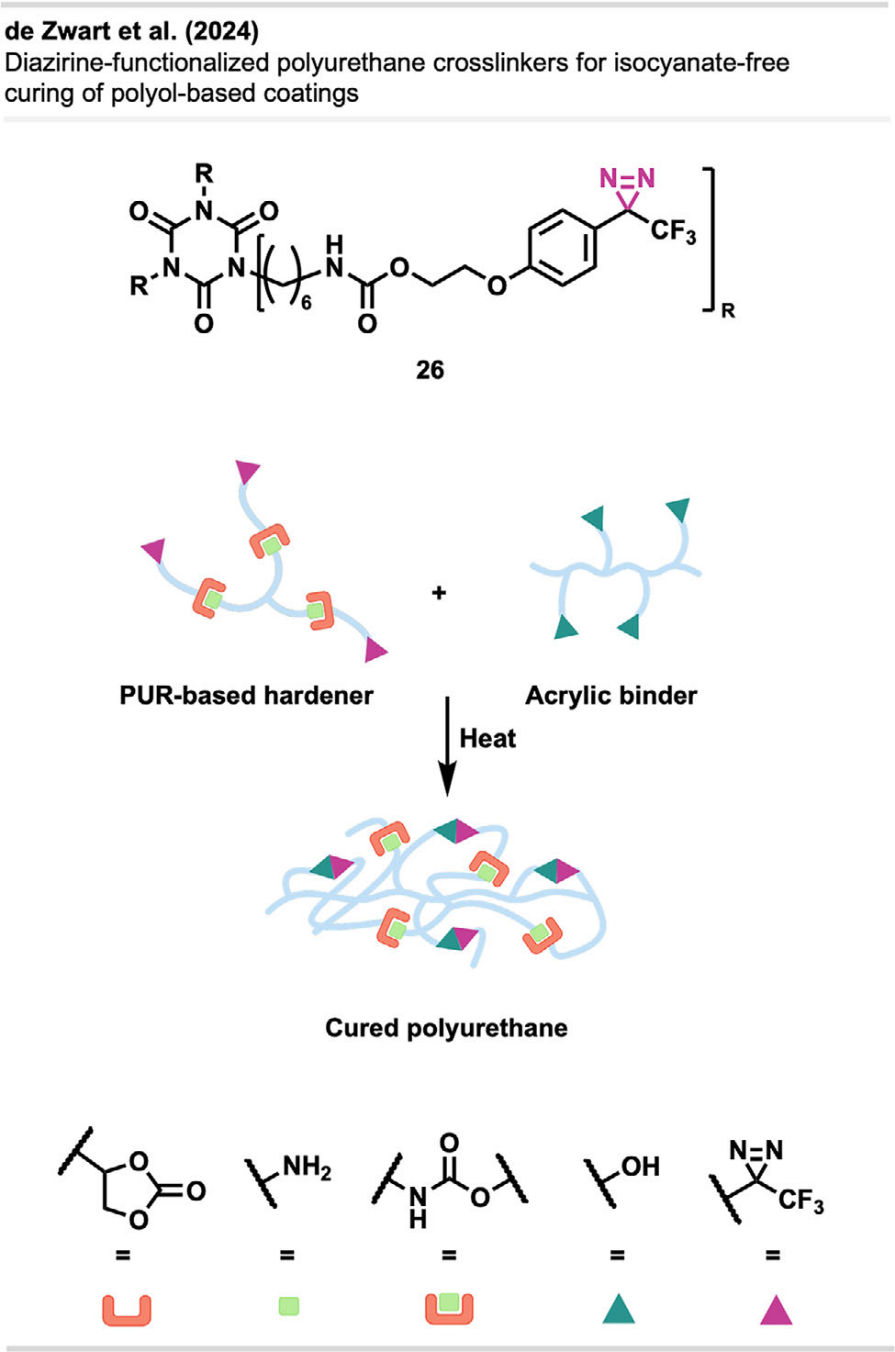
Crosslinker **26** consists of a commercial isocyanurate trimer core functionalized with three ethylene glycol‐tethered diazirine groups. This enables trifunctional carbene generation upon thermal activation for covalent insertion into polyol O─H bonds. The figure was redrawn and adapted from de Zwart et al.,^[^ ^64^ ^]^ licensed under CC BY. © 2024 The Authors of the original publication. Created with BioRender.com.

Xu et al.^[^ ^63^ ^]^ further demonstrated the approach's applicability by efficiently crosslinking unfunctionalized commodity polymers such as PDMS blends and several other polymers. A diazirine‐functionalized PDMS crosslinker **25** (Figure 16a) was synthesized via hydrosilylation using diazirine precursor **24** with near‐complete conversion, demonstrating the compatibility of diazirines with silicone chemistry. While unmodified PDMS showed no crosslinking under UV‐light irradioation, blending **25** with thiol‐functionalized PDMS achieved gel fractions of 58% (Figure 16b). Notably, polymeric diazirines outperformed bis‐diazirine small molecules, which failed at comparable loadings.

de Zwart et al.^[^ ^64^ ^]^ introduced a diazirine‐functionalized polyurethane crosslinker **26** (Figure 17) as a safer, isocyanate‐free alternative for polyol‐based coatings. Upon heating, carbenes inserted into O─H bonds of acrylic polyols, forming robust networks with mechanical properties comparable to traditional systems. The coatings showed high storage modulus, good thermal stability, and long shelf life, even when premixed, highlighting the promise of diazirine curing for sustainable, one‐component, high‐performance coatings.

### 4.2 Diazirines as Crosslinkers for Electronic Materials and Devices

Diazirine‐based crosslinkers are gaining momentum as powerful tools in the fabrication of electronic materials and devices. They show great potential for photopatterning and enable reliable solution processing, improved layer stability, and precise deposition in multilayer architectures. Their compatibility with standard fabrication techniques, alongside high spatial resolution and solvent resistance, makes them particularly attractive in organic electronics. Beyond this, diazirine‐based approaches have shown broader utility in fields such as quantum dots and perovskite solar cells (PSCs). Their versatility and processability position diazirines as a transformative platform for next‐generation electronics.

Solution processing is a cost‐effective method for fabricating organic electronics, where materials are deposited from solution onto a surface or layer by layer (e.g., by spin coating), but it often suffers from solvent‐induced damage, layer mixing, and uneven interfaces.^[^ ^65^, ^66^, ^67^ ^]^ Diazirine‐based photocrosslinkers address these issues by forming highly crosslinked, insoluble networks, allowing the deposition of layers without limitations for solvents used in the subsequent layer. This improves structural integrity and enables reliable multilayer fabrication in organic electronic devices.

**Figure 18:**
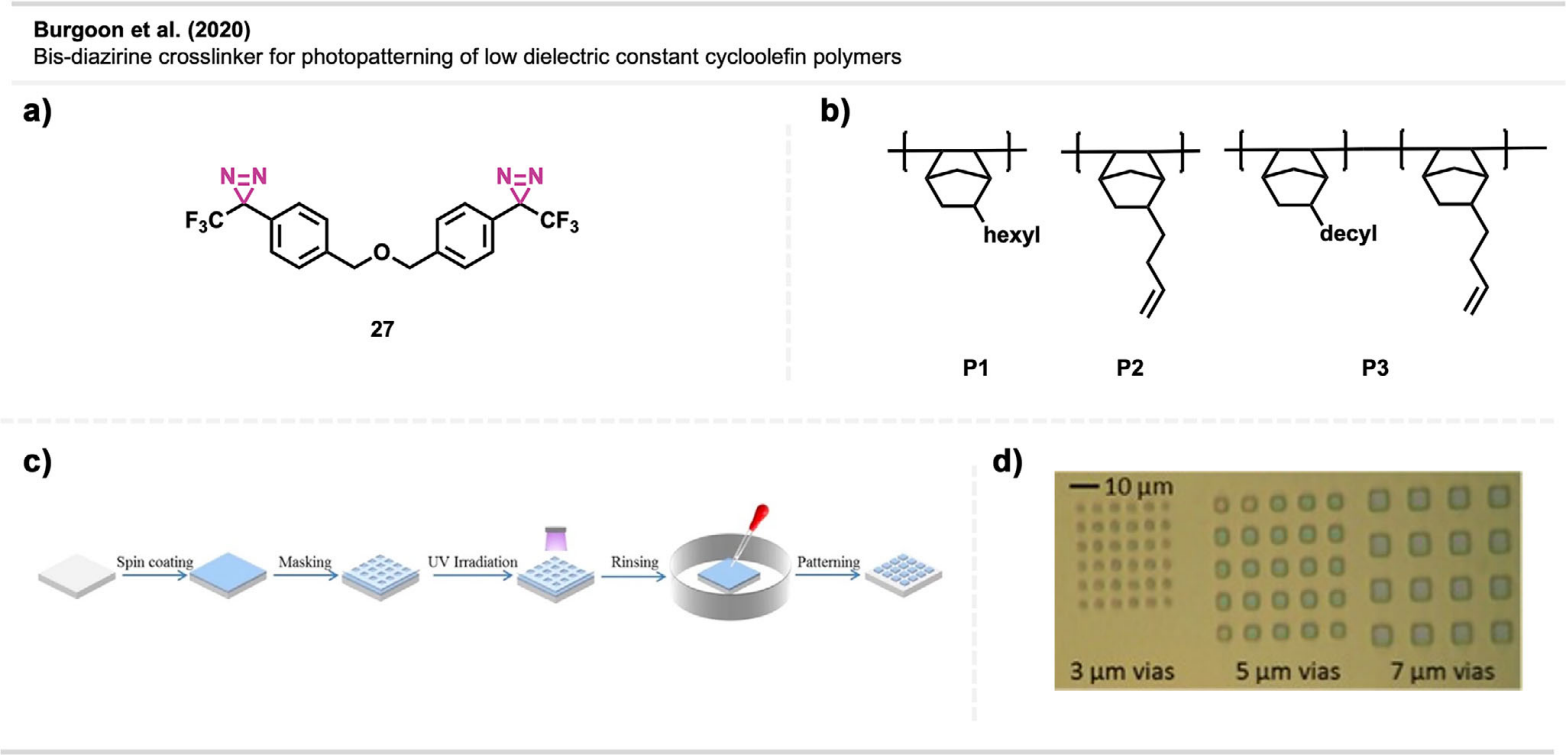
a) Bis‐diazirine crosslinker **27**. b) Cycloolefin polymers **P1**–**P3**. c) Representative depiction of photopatterning workflow. d) Representative photomicrograph of **P1**/crosslinker **27** formulation, demonstrating pattern resolution of 3–7 µm via holes. Section c is a direct reproduction from Wu et al.,^[^ ^69^ ^]^ permission acquired via RightsLink. © American Chemical Society. Section d is a direct reproduction from Burgoon et al.,^[^ ^68^ ^]^ permission acquired via RightsLink. © 2020 American Chemical Society.

Burgoon et al.^[^ ^68^ ^]^ applied bis‐diazirine crosslinker **27** (Figure 18a) in the photopatterning of low dielectric constant cycloolefin polymers **P1**–**P3** (Figure 18b,c), which are attractive insulators for electronic wiring applications. By crosslinking these polymers using photopatterning techniques, the materials can be readily integrated into electronic devices. The polymer solutions are spin‐coated onto silicon wafers, and after solvent removal, the films are exposed to 365 nm light through a mask to afford patterned via holes (vertical electrical connections between conductive layers in a printed circuit board [PCB]) (Figure 18d). The illuminated areas form three‐dimensional, crosslinked polymer networks no longer soluble in the developer solvent. Image‐wise photopatterning was successfully achieved for unreactive, aliphatic cycloolefin films containing the crosslinker. Notably, these diazirine‐crosslinked films exhibited substantially lower dielectric constants compared to films crosslinked with an azide‐based photocrosslinker, highlighting the superior performance of diazirine chemistry.

**Figure 19:**
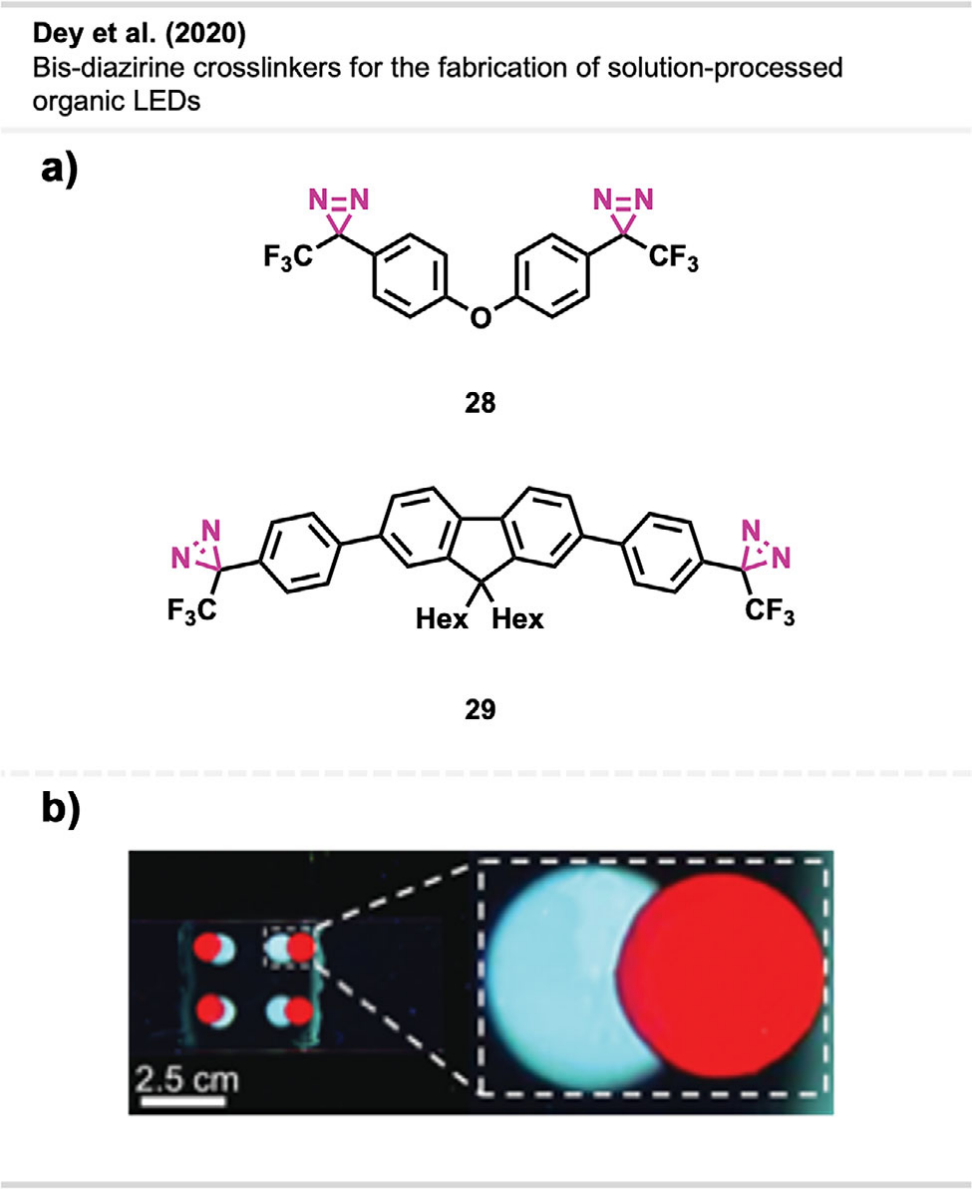
a) Crosslinkers **28** and **29** are used for crosslinking electroluminescent conductive polymers. b) Formed pixel overlap without mixing. Section b is a direct reproduction from Dey et al.,^[^ ^70^ ^]^ permission acquired via RightsLink. © 2020 Royal Society of Chemistry.

Dey et al.^[^ ^70^ ^]^ proved the potential of diazirine crosslinkers **28** and **29** (Figure 19a) in solution processing with the photolithographic patterning of the electroluminescent conductive polymers dioctyl polyfluorene (PFO) and regioregular poly(3‐hexylthiophene‐2,5‐diyl) (P3HT). Here, the red pixel (P3HT) was deposited on top of the blue pixel (PFO) in a way that the two pixels partly overlap (Figure 19b). The fluorescent image shows that the pixels were deposited in well‐defined layers, without interlayer mixing or interpenetration of fluorescent light. Furthermore, the diazirine‐based crosslinking method also substantially improved OLED performance compared to alternative crosslinking methods in the literature.

The same group demonstrated that diazirine‐based crosslinking significantly enhances the surface morphology and performance of polymer light‐emitting diodes (PLEDs) using polyvinyl carbazole (PVK)‐based fluorescent polymers. Incorporating 10% of diazirine crosslinker **28** improved film uniformity and performance in PVK‐based PLEDs, reducing surface roughness without altering chemical or electronic properties. This simple, low‐cost, and air‐compatible method offers a promising route to enhance OLED device fabrication.^[^ ^71^ ^]^

**Figure 20:**
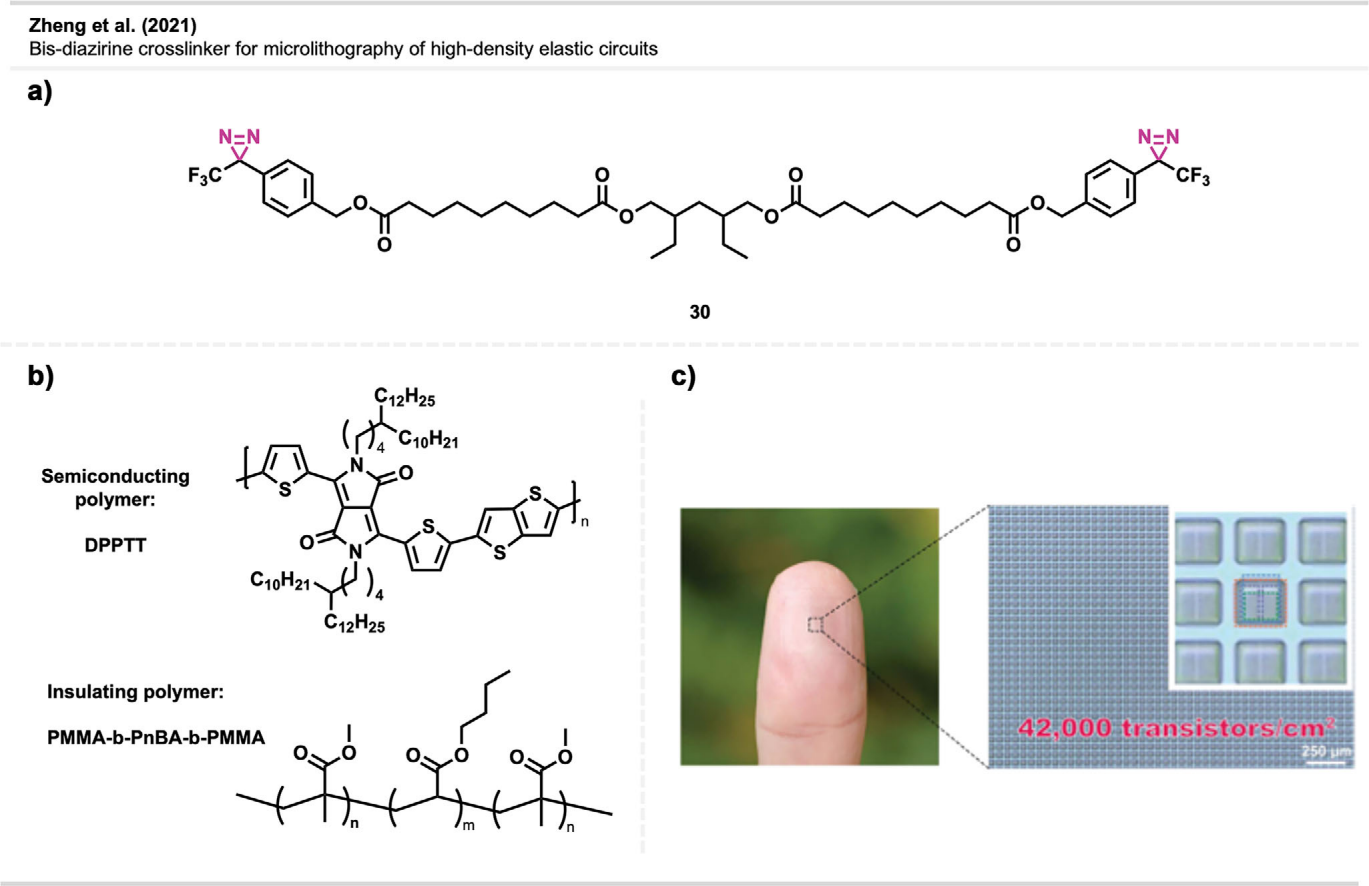
a) Crosslinker **30**. b) A semiconducting diketopyrrolopyrrole‐thienothiophene (DPPTT) polymer and polymethylmethacrylate (PMMA) and poly(*n*‐butyl acrylate) as an insulating triblock copolymer. c) Representative picture of flexible electronic material. Section c is a direct reproduction from Zheng et al.,^[^ ^72^ ^]^ permission acquired via RightsLink. © 2021 The Authors of the original publication.

Zheng et al.^[^ ^72^ ^]^ showed the carbene‐mediated optical lithography of semiconducting polymers for the fabrication of elastic electronic materials in micrometer‐scale resolution. The tethered bis‐diazirine **30** (Figure 20a) facilitated the sequential deposition of conducting and insulating polymers (Figure 20b) to form complex, high‐density, multilayered elastic circuits. With this process, they have successfully built flexible transistors with short channel lengths and increased transistor density by more than two orders of magnitude higher than earlier benchmarks (Figure 20c).^[^ ^73^ ^]^ This strategy provides a general, scalable route for all‐solution‐processed, photopatterned, flexible electronic devices with high fidelity and operational stability.

**Figure 21:**
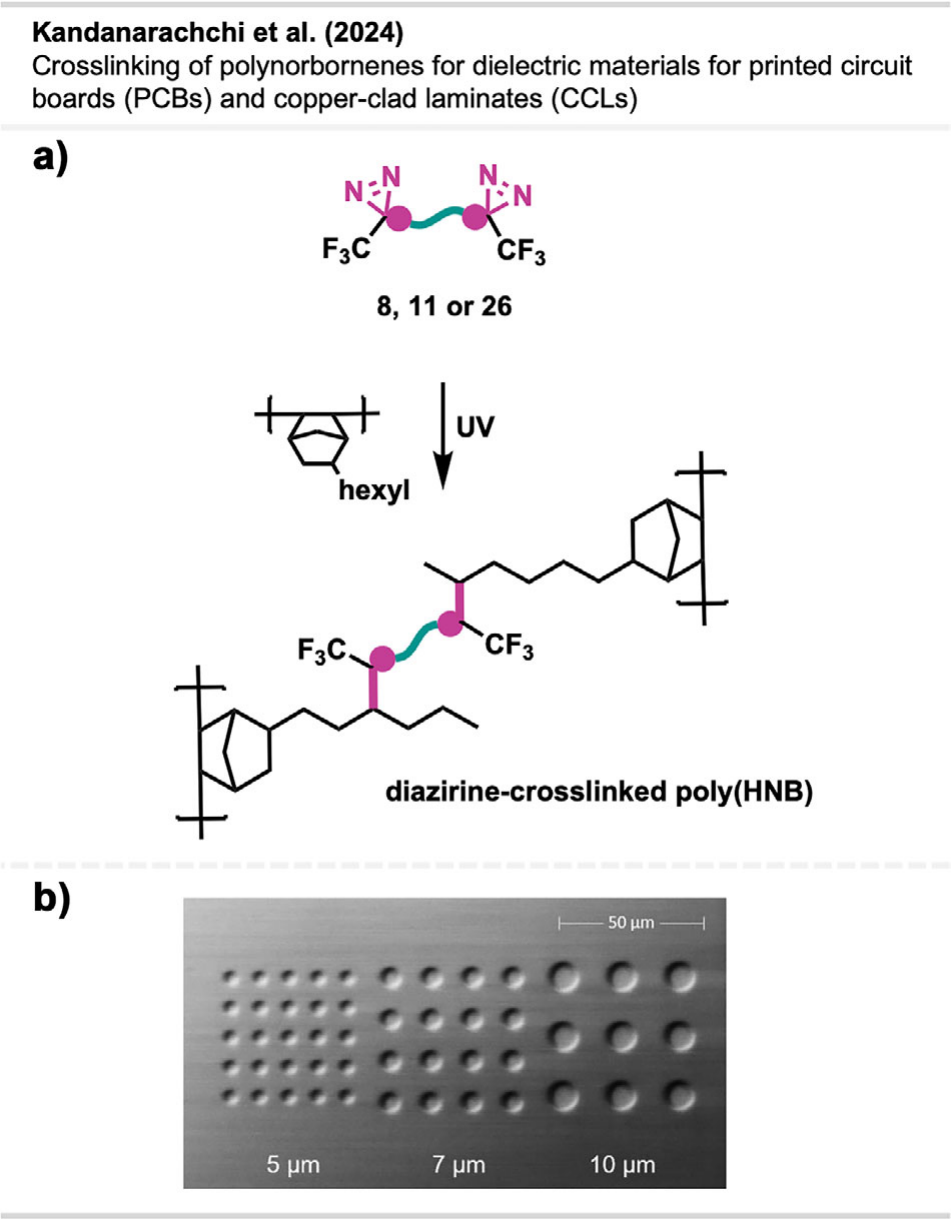
a) Crosslinking of poly(NHB). b) Representative SEM images of poly(HNB) photopatterned vias with diazirine **11**. Section b is a direct reproduction from Kandanarachchi et al.,^[^ ^74^ ^]^ licensed under CC BY. © 2024 The Authors of the original publication.

Kandanarachchi et al.^[^ ^74^ ^]^ investigated the use of difunctional diazirine crosslinkers **8**, **11**, and **27** for crosslinking of polynorbornenes (poly(HNB)) as next‐generation dielectric materials for PCBs and copper‐clad laminates (CCLs) (Figure 21a). The resulting thermosets exhibit low dielectric constants, low dielectric loss, and excellent thermal stability (up to 338  °C), meeting key requirements for high‐frequency electronics. Among the tested crosslinkers, diazirine **11** demonstrated the highest photopatterning efficiency and lowest activation temperature. Diazirine‐crosslinked poly(HNB) showed strong copper adhesion and THF‐insolubility, enabling high‐resolution patterning for pillars (3 µm) and vias (5 µm) on smooth copper for PCB applications (Figure 21). This system offers a processable, photopatternable alternative to PTFE in semiconductor packaging and microelectronics.

Due to their tunable performance and material flexibility, organic electronics have rapidly expanded into logic circuits,^[^ ^75^ ^]^ bioelectronics,^[^ ^76^ ^]^ and thermoelectrics.^[^ ^77^ ^]^ Currently, memory and neuromorphic computing dominate research in this field. Organic field‐effect transistor (OFET) memory stands out for its lightweight, low‐cost, and flexible charge storage capabilities. Ongoing improvements in charge mobility and stability have further enhanced OFET functionality, positioning them as key players in next‐generation electronic technologies.^[^ ^75^, ^78^ ^]^

Patterning organic semiconductors is crucial for high‐performance OFETs, yet it remains challenging in fully solution‐processed electronics due to solvent incompatibility. Recent approaches using azide‐based crosslinkers enable precise photo‐patterning and improve compatibility by preventing damage to underlying semiconductor layers.^[^ ^79^, ^80^ ^]^

**Figure 22:**
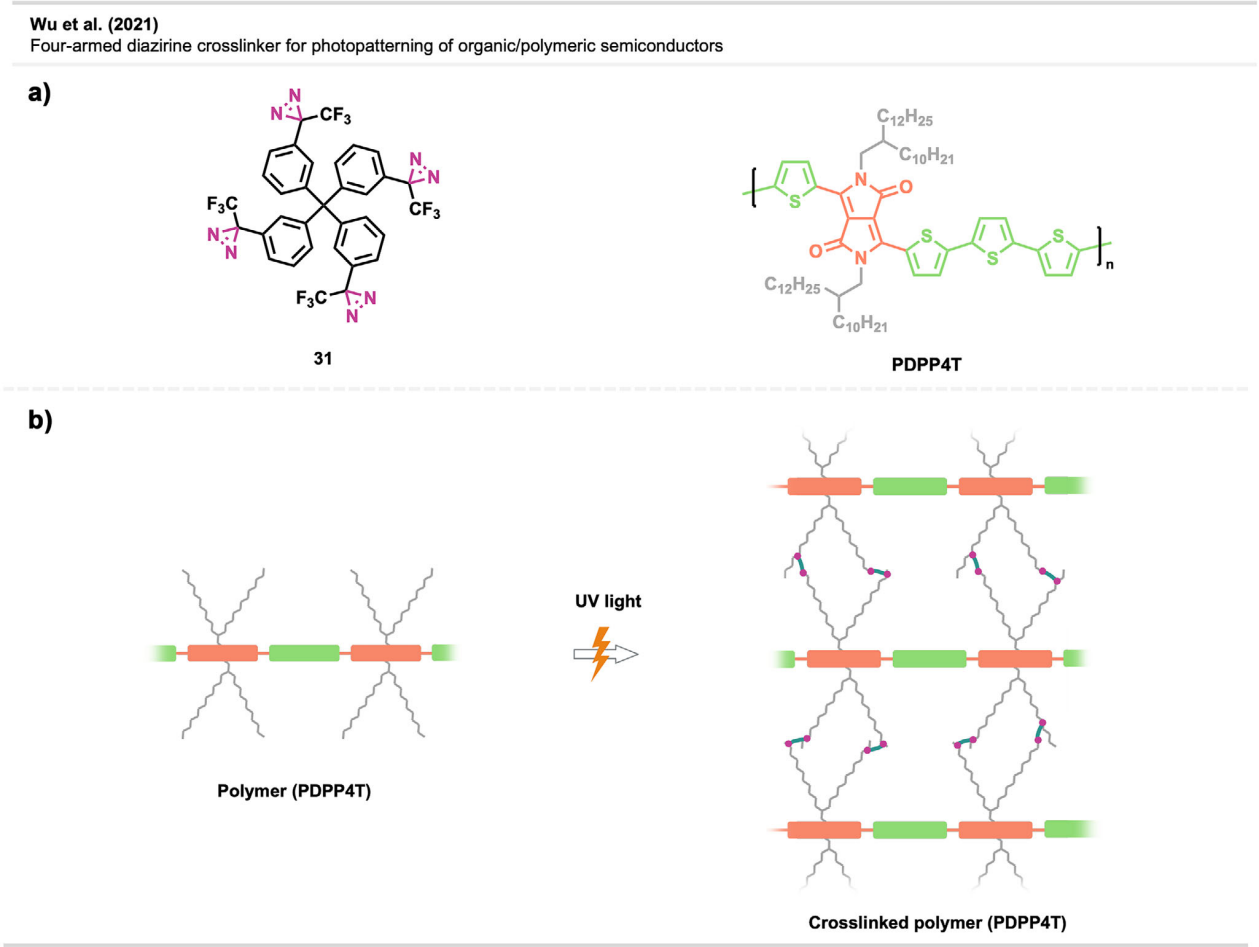
a) Four‐armed diazirine linker **31** and representative semiconducting polymer PDPP4T. b) Crosslinked semiconducting polymer. Section b was redrawn and adapted from Wu et al.,^[^ ^69^ ^]^ permission acquired via RightsLink. © 2021 Wiley.

Wu et al.^[^ ^69^ ^]^ used diazirine‐based four‐armed crosslinker **31** for the photopatterning of semiconducting polymers such as PDPP4T (Figure 22a). Due to its four diazirine moieties with tetrahedral geometry, **31** can efficiently crosslink polymers with small loadings. The method successfully patterned p‐, n‐, and ambipolar semiconducting polymers with aliphatic side chains via carbene‐induced C─H insertion (Figure 22b). The crosslinked layers were robust and well‐defined, and showed good resistance to organic solvents. Hence, **31** is a promising crosslinker for all‐solution‐processed (i.e., allowing fully solution‐processed device manufacture) flexible electronic devices.

**Figure 23:**
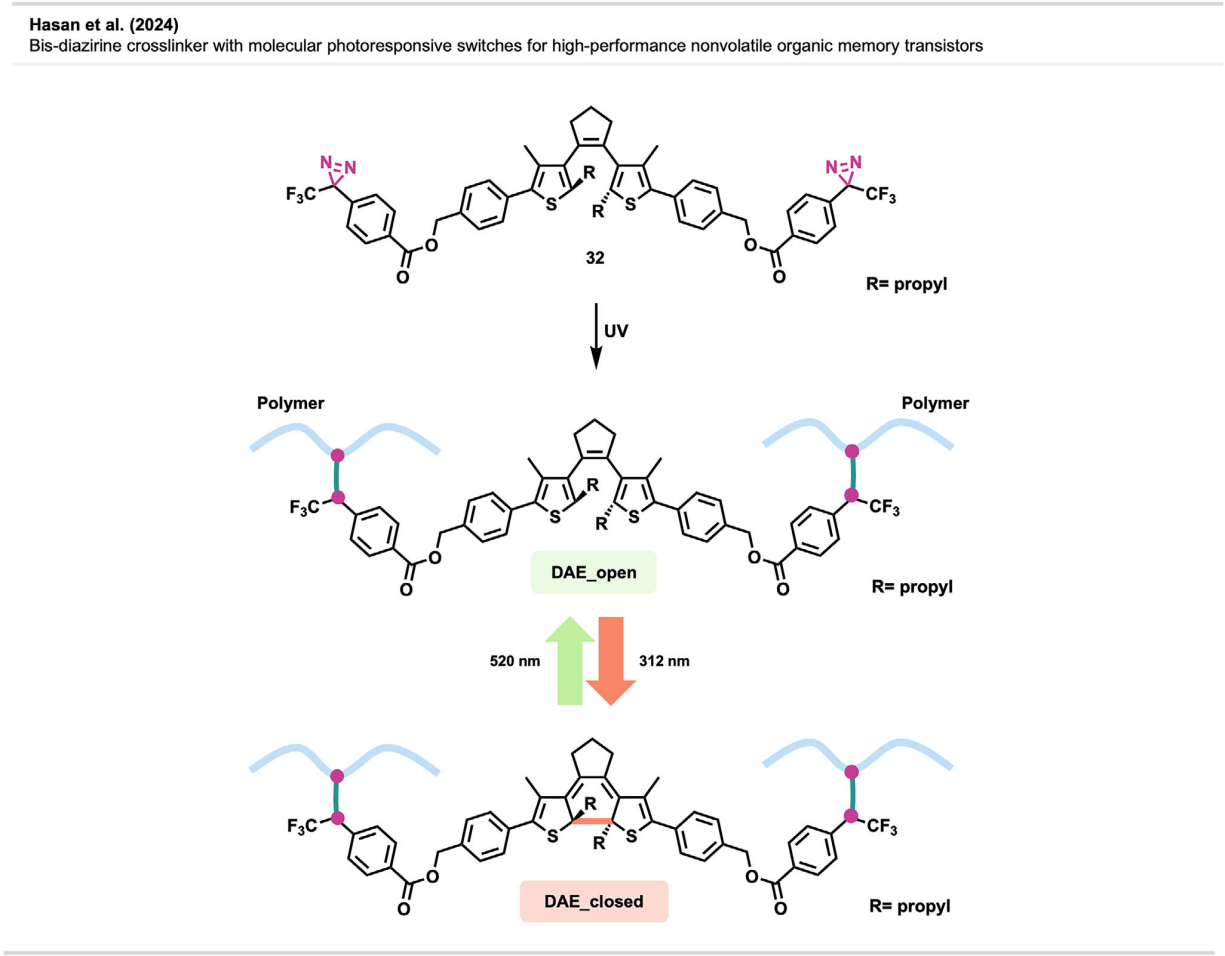
UV‐activated crosslinking of **32** to a polymer, both open (DAE_open) and closed (DAE_closed) forms, with reversible photoswitching behavior. The figure was redrawn and adapted from Hassan et al.,^[^ ^81^ ^]^ licensed under CC BY. © 2024 The Authors of the original publication. Created with BioRender.com.

Recent advances in OFETs have fueled efforts to expand their functionality, particularly by integrating photoresponsive molecular switches. One promising strategy involves using diarylethenes (DAEs), which undergo reversible structural changes upon light exposure (Figure 23). These photochromic switches can modulate trap states in organic semiconductors, enabling photoprogrammable behavior. In their open form (DAE_open), DAEs have limited π‐conjugation, while the closed form (DAE_closed) features extended π‐conjugation and a reduced HOMO–LUMO gap, allowing it to act as an efficient charge trap. Their bistability makes them ideal for multifunctional OFET applications.

This study^[^ ^81^ ^]^ presents a novel approach using photophore‐anchored DAE molecular switches that function dually as molecular switches and photocrosslinkers in memory‐type OFETs. By incorporating diazirine moieties **32** (and azide functionalities, not shown) (Figure 23), these compounds enable UV‐triggered covalent attachment to polymer semiconductors, effectively stabilizing the closed isomeric form (DAE_closed) as deep trap states. When blended with the polymer semiconductor F8T2, the system demonstrated excellent device performance. Notably, the diazirine‐containing switch outperformed its azide counterpart due to more favorable crosslinking behavior. The crosslinked films exhibited enhanced morphological stability, operational robustness, and high‐resolution photopatternability. Covalent anchoring suppressed phase separation, enabling superior retention and long‐term reliability. Furthermore, the patterned films retained their optical and electronic properties, highlighting compatibility with scalable microelectronics manufacturing.

Organic electrochemical transistors (OECTs) are also emerging as versatile tools in bioelectronics, valued for their ability to transduce and amplify ionic and biological signals into electronic outputs.^[^ ^82^, ^83^ ^]^ OECTs rely on organic mixed ionic‐electronic conductors (OMIECs), which transport both ionic and electronic charge carriers, ideal for bridging biological processes with electronic systems. OMIECs also offer mechanical flexibility, aqueous compatibility, and cytocompatibility, making them suitable for applications such as neural interfaces and biosensors.^[^ ^84^, ^85^ ^]^ Efficient OECT operation depends on well‐designed channels that support both ionic doping/dedoping and electronic charge transport. Incorporating photo‐patternable materials is essential to minimize parasitic capacitance and simplify fabrication. However, achieving precise patterning while maintaining control over ionic‐electronic coupling remains a major challenge.^[^ ^86^ ^]^ Recently, a vertical organic electrochemical transistor (vOECT) architecture demonstrated high transconductance and excellent cycling stability using cinnamate‐cellulose as a photo‐crosslinker to simplify channel fabrication.^[^ ^87^ ^]^

**Figure 24:**
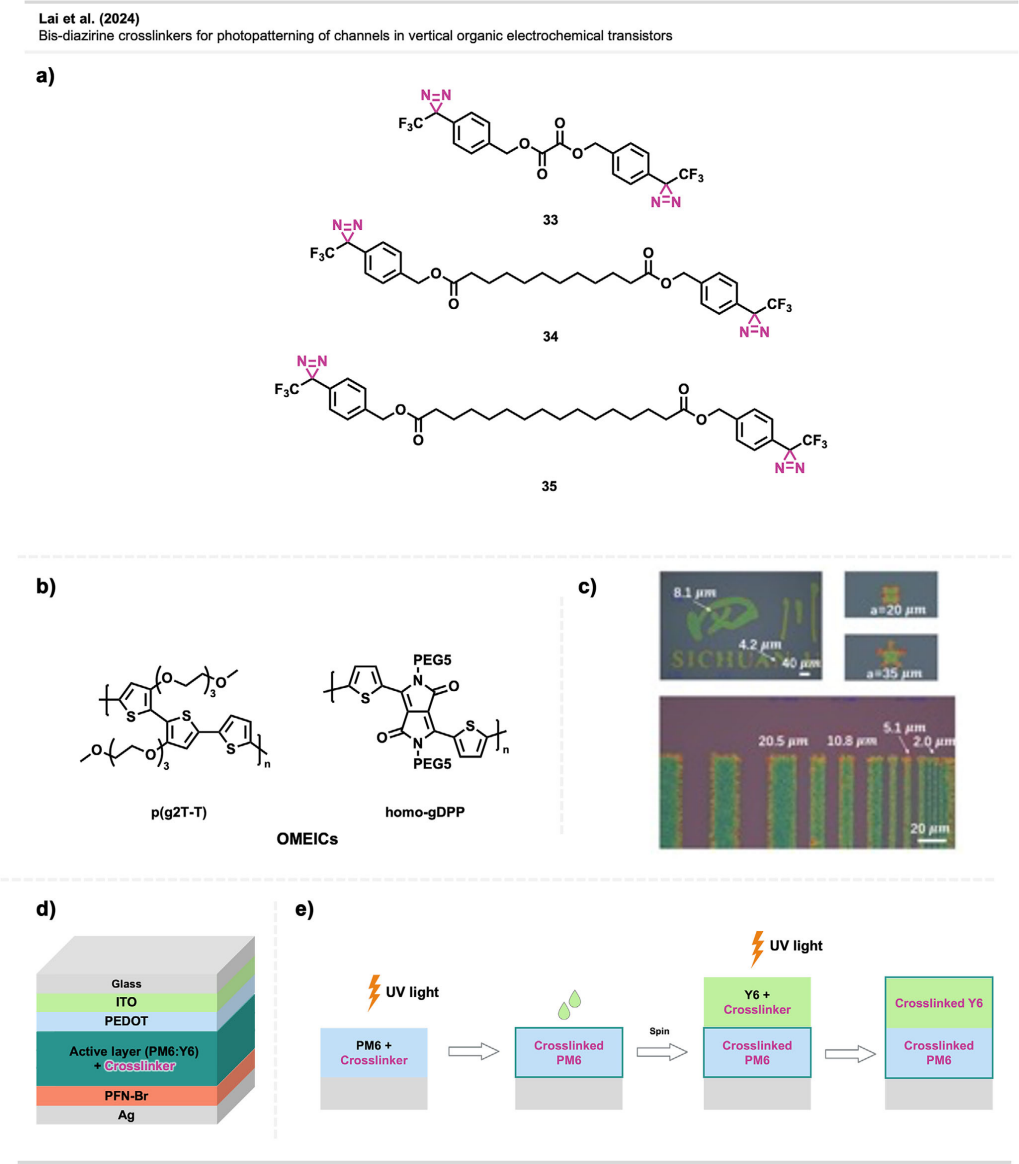
a) Crosslinker **33**–**35** b) Structures of OMIECs used in OECTs. c) Optical microscope images of patterned polymer blends using crosslinker **35**. d) Schematic depiction of an OPV. The multilayer architecture consists of a glass substrate, followed by a transparent anode of indium tin oxide (ITO) and a hole transport layer of poly(3,4‐ethylenedioxythiophene) polystyrene sulfonate (PEDOT:PSS). The active layer comprises a BHJ blend of PM6 and Y6, with a diazirine crosslinker **34**. Below the active layer is a cathode interlayer of poly[(9,9‐bis(3′‐(*N,N*‐dimethylamino)propyl)‐2,7‐fluorene)‐alt‐2,7‐(9,9‐dioctylfluorene)] bromide (PFN‐Br) with a silver (Ag) electrode. e) Stepwise fabrication of a bilayer OPV film using diazirine‐crosslinker 34 for vertical phase stabilization. First, a PM6:2Dz layer is deposited and crosslinked via UV irradiation. A second layer of Y6:2Dz is then spin‐coated on top and subsequently UV‐crosslinked. This sequential crosslinking locks the vertical composition gradient in place, preventing interdiffusion and improving device stability. Section c is a direct reproduction from Lai et al.,^[^ ^86^ ^]^ permission acquired via RightsLink. © 2024 The authors of the original publication. Sections d and e were redrawn and adapted from Suzuki et al.,^[^ ^88^ ^]^ permission acquired via RightsLink. © 2023 American Chemical Society. Created with Biorender.com.

A study by Lai et al.^[^ ^86^ ^]^ aimed to demonstrate that diazirine‐based crosslinkers offer a more straightforward synthesis, along with enhanced formulation flexibility and a broader processing window, providing a more practical and efficient strategy for device fabrication compared to other existing methods. This work introduced bis‐diazirine crosslinkers **33**–**35** (Figure 24a) with varying alkyl chain lengths for patterning OMIEC channels in OECTs based on p(g2T‐T) and Homo‐gDPP (Figure 24b). The crosslinkers enabled micrometer‐scale patterning and tuning of channel structure in OECTs while also modulating ionic‐electronic coupling to enhance doping/dedoping control, charge transport, and overall device performance.

The diazirine‐based approach for vOECT offered high reactivity, fine patterning resolution (∼2 µm), and no need for photo‐initiators (Figure 24c). The crosslinkers selectively reacted with OMIEC side chains without damaging the conjugated backbone. Resulting devices exhibited excellent performance—high transconductance, on/off ratio, and cycling stability (>100 000 cycles)—demonstrating their strong potential for bioelectronic applications.

Suzuki et al.^[^ ^88^ ^]^ published a critical study on the performance of bulk heterojunction (BHJ) organic photovoltaics (OPVs) using diazirine **34** and an azide‐based crosslinker (not shown). OPV devices convert light into electricity using organic semiconductors. A common architecture is the BHJ, where donor and acceptor materials are blended to form a single active layer (Figure 24d,e). Materials such as donor polymer PM6 and non‐fullerene acceptor Y6 have pushed power conversion efficiencies to around 19%.^[^ ^89^ ^]^

In PM6:Y6 BHJ organic photovoltaics, they observed a 35% efficiency loss after UV exposure, attributed to side reactions during crosslinking. Although the findings raise important concerns about crosslinker compatibility, further studies across varied material systems are needed to fully evaluate both the potential and the limitations of universal photocrosslinking strategies in organic photovoltaics.

**Figure 25:**
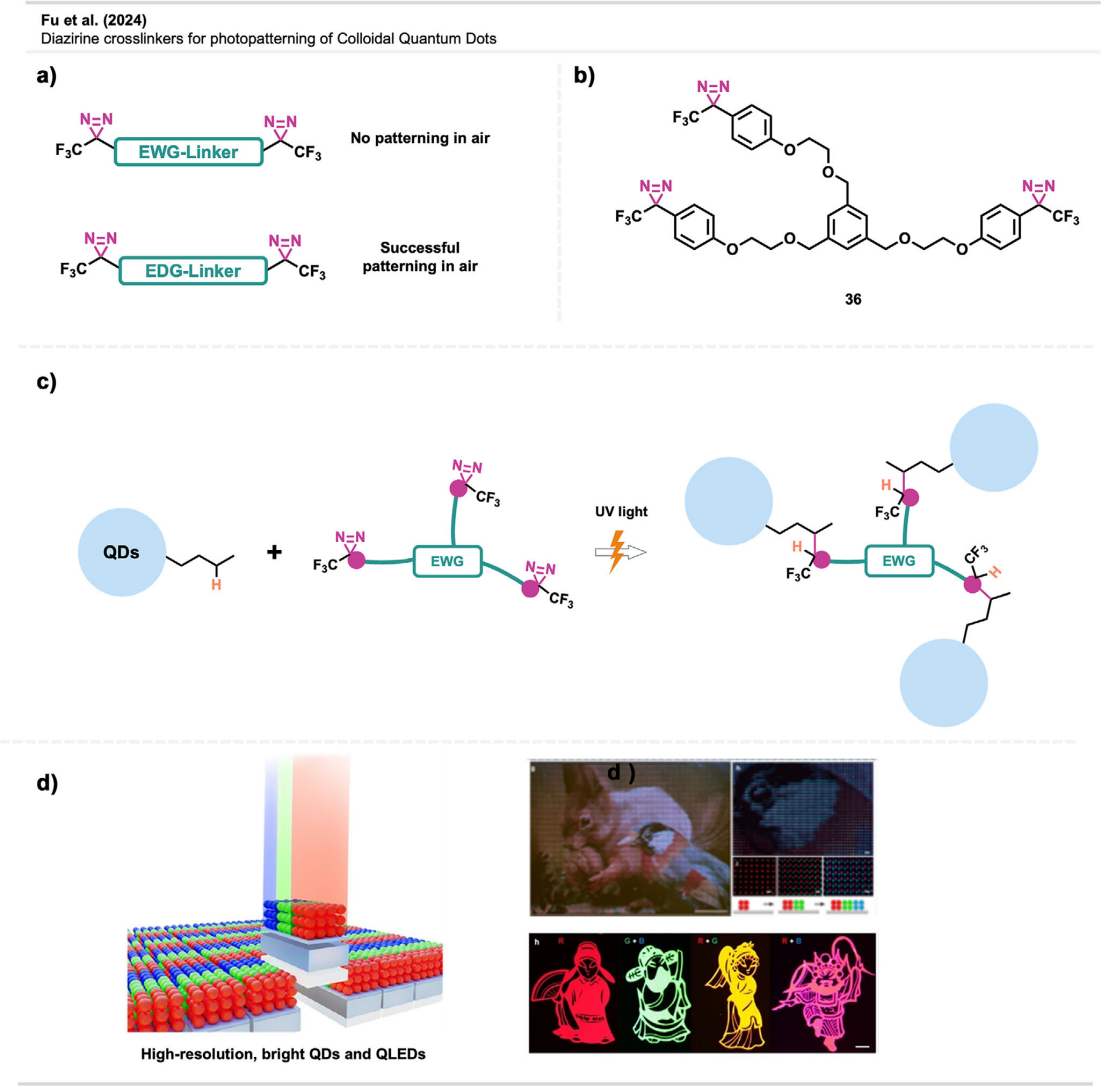
a) Diazirine crosslinkers bearing EWGs are unsuitable for QD photopatterning in air due to triplet carbene formation and subsequent quenching by oxygen. In contrast, crosslinkers with EDGs were successfully patterned under ambient conditions. b) Schematic depiction of QDs, QLEDs, and their crosslinking. c) Three‐armed crosslinker **36** showed the best results in QD photopatterning. d) Left: Schematic depiction of photopatterned QDs and QLEDs. Top Right: Fluorescence microscopy images of sequentially arranged RGB QD patterns (1700 ppi) fabricated using compound **36** under ambient air conditions. Bottom Right: Fluorescence microscopic images of stacked QD layers to show mixed colors. Sections a–c were redrawn and adapted from Fu et al.,^[^ ^90^ ^]^ while Section d is a direct reproduction from the same work. Permission acquired via RightsLink. © 2024 American Chemical Society. Created with Biorender.com.

A study by Fu et al.^[^ ^90^ ^]^ introduced an electronically optimized diazirine‐based crosslinking strategy for the high‐resolution photopatterning of colloidal QDs, enabling the scalable fabrication of next‐generation QLED displays. The authors developed diazirine crosslinkers based on trifluoromethyl aryl frameworks with electron‐donating groups (EDGs) to achieve efficient and nondestructive patterning under ambient conditions. This promoted singlet carbene formation, suppressing oxygen‐induced side reactions observed with diazirine crosslinkers bearing electron‐withdrawing groups (EWGs) such as the polyfluorinated bis‐diazirines **9** (Figure 25a). Photogenerated singlet carbenes covalently bridged adjacent QDs by inserting into C─H bonds of native ligands such as alkyl carboxylates, amines, or thiols (Figure 25b). Among the tested crosslinkers, **36** (Figure 25c) exhibited the highest UV absorption and patterning efficiency, achieving ∼90% film retention at low UV doses, outperforming other tested crosslinkers. The use of three reactive groups per crosslinker molecule enabled efficient crosslinking with minimal reagent, preserving the optical and electronic properties of the QDs. Under ambient conditions, these crosslinkers successfully enabled the patterning of heavy metal‐free QDs. Using this method, the authors fabricated microarrays, RGB subpixels, and 3D‐stacked QD structures with sub‐micrometer resolution (>13 000 pixels per inch) (Figure 25d). AFM and SEM confirmed uniform morphology with minimal material loss. Notably, InP/ZnSe/ZnS QLEDs fabricated with this method displayed superior device performance compared to other patterning techniques. The process is compatible with flexible substrates and holds strong potential as a breakthrough technology for commercial QLED manufacturing.

**Figure 26:**
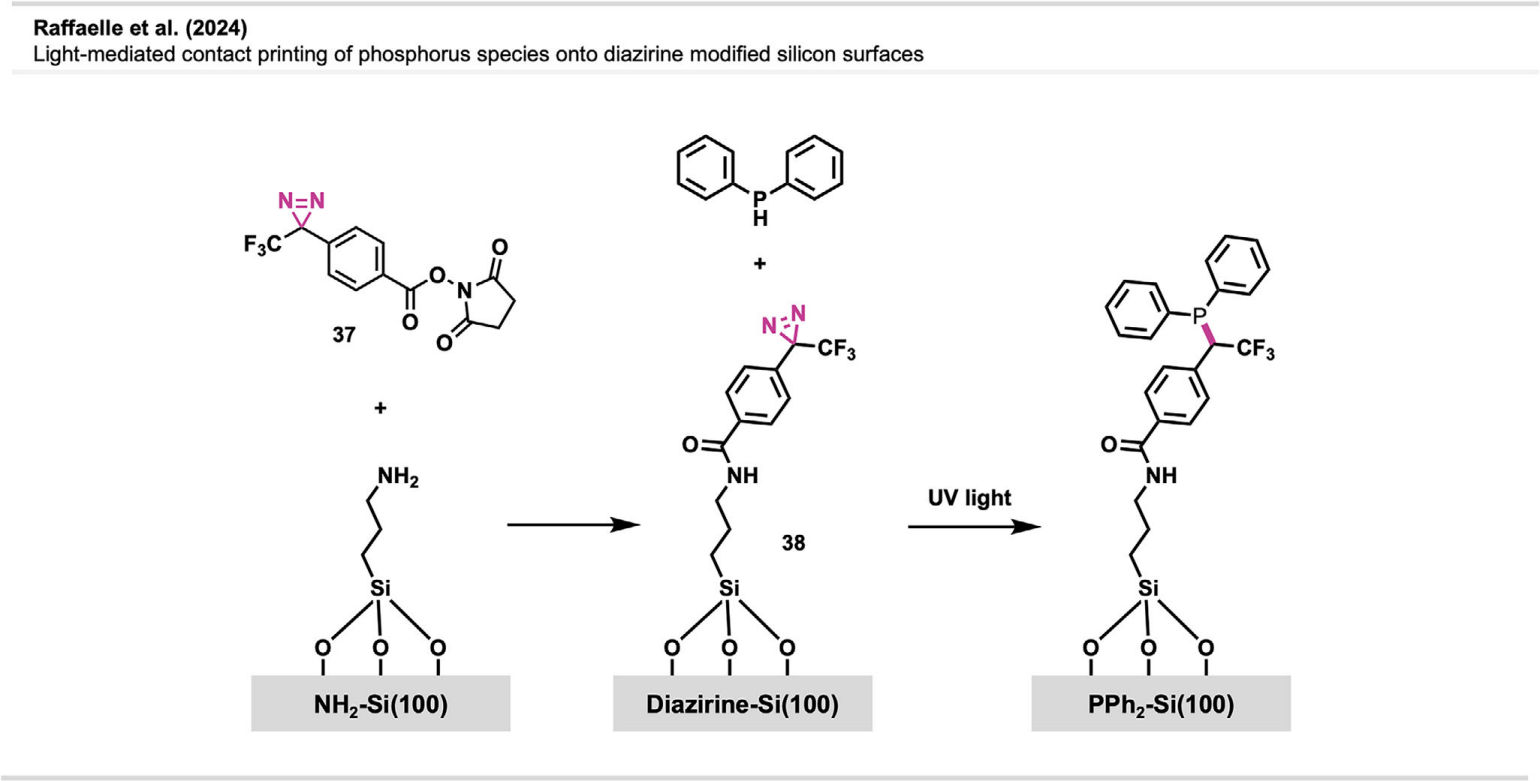
UV‐induced carbene insertion enables contact printing of diphenylphosphine onto diazirine‐functionalized silicon surfaces. The figure was redrawn and adapted from Raffaelle et al.,^[^ ^91^ ^]^ licensed under CC BY. © 2024 The authors of the original publication.

A study by Raffaelle et al.^[^ ^91^ ^]^ introduced a novel light‐activated contact printing method for precise phosphorus doping of silicon(100) using diazirine‐based carbene chemistry. A two‐layer molecular system—comprising a stabilizing aminosilane sublayer substituted with NHS‐diazirine **37** to form a diazirine‐functionalized overlayer **38**—enabled UV‐induced carbene formation and covalent attachment of phosphorus dopants. Carbenes facilitated direct C─P bond formation by inserting into P─H bonds of diphenylphosphine (Figure 26). This approach enabled selective and covalent immobilization of phosphorus‐containing molecules through contact printing, allowing for precise surface patterning and doping in electronic manufacturing.

### 4.3 Perovskite Solar Cell

Since their introduction in 2009, PSCs have gained significant attention as a promising alternative to silicon photovoltaics. Their rapid power conversion efficiency (PCE) gains—from 3.8% to 26.7% in just over a decade—underscore their potential, with tandem architectures combining perovskite cells and established silicon technology now surpassing single‐junction silicon cells and achieving efficiencies as high as 33.7%. Composed of abundant elements and compatible with scalable fabrication methods, PSCs combine low production costs, simple processing, and mechanical flexibility, positioning them as a highly attractive technology for future large‐scale solar energy deployment.^[^ ^92^, ^93^, ^94^, ^95^, ^96^ ^]^

**Figure 27:**
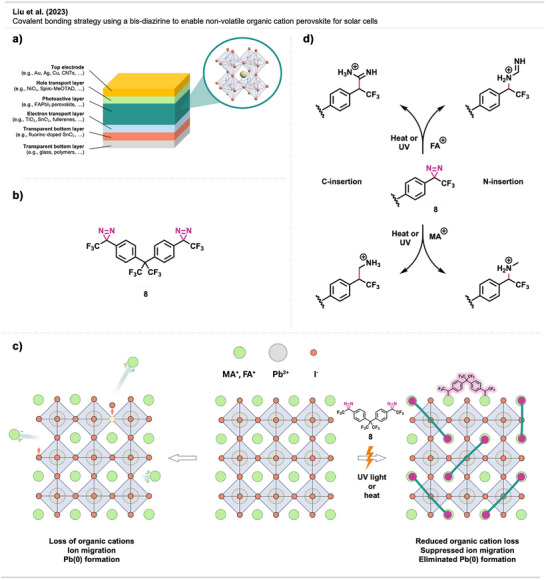
a) PSC architecture. b) Molecular mechanisms for diazirine‐based stabilization of PSCs. c) Perovskite lattice. The left panel shows the loss of organic cations and the migration of I^−^ ions. Right panel: Stabilized perovskite through diazirine crosslinking. Section c was redrawn and adapted from Liu et al.,^[^ ^102^ ^]^ permission acquired via RightsLink. © 2023 Elsevier.

The typical architecture of a conventional n‐i‐p‐type PSC consists of a glass or polymer substrate, a transparent electrode, a charge‐selective layer (n‐type), the perovskite absorber layer, a second charge‐selective layer (p‐type), and a top electrode (Figure 27a).

Crucial to PSC performance is the metal halide perovskite photoactive thin film, which absorbs light and converts it into electricity. Perovskites are crystalline materials with the general formula ABX_3_, in which A is a monovalent cation, e.g., methylammonium (MA^+^), formamidinium (FA^+^), or caesium; B is a divalent metal cation, commonly lead or tin; and X is a halide anion, such as chloride, bromide, or iodide. Among various perovskite formulations, FAPbI_3_‐based structures have set efficiency records due to their near‐optimal bandgap and enhanced thermal stability.^[^ ^92^ ^]^

Several challenges must be addressed to enhance the applicability and commercialization of this technology. Environmental stressors—such as heat, moisture, and mechanical strain—accelerate degradation by promoting ion migration and phase segregation, ultimately reducing efficiency and longevity. Organic cations such as FA⁺ and MA⁺ are prone to escaping from grain boundaries under thermal or operational stress, which significantly impairs device performance. In addition, trace organic vapors like MA and FA can accumulate beneath the gold electrode, causing surface cracking. The loss of organic cations also generates vacancies that promote iodide ion migration, destabilizing the perovskite lattice and accelerating metallic lead formation, ultimately undermining long‐term stability and overall device reliability.^[^ ^97^, ^98^, ^99^, ^100^, ^101^ ^]^

To tackle these problems, Liu et al.^[^ ^102^ ^]^ reported a covalent bonding strategy using bis‐diazirine **8** for stabilizing PSCs (Figure 27b). By forming covalent bonds with organic cations, this approach effectively immobilized volatile species, preventing their loss under thermal and operational stress (Figure 27c). As a result, ion migration and vacancy formation were significantly reduced, stabilizing the perovskite lattice and suppressing the formation of metallic lead (Pb⁰). This improved device efficiency, structural integrity, and long‐term operational stability.

Crosslinker **8** closely matches the lattice size of formamidinium (FA)‐based perovskites (9.01 Å). During the high‐temperature annealing process, the reactive carbenes are formed and covalently bind to FA⁺ and MA⁺ cations through C─H and N─H insertion (Figure 27d). Covalent bond formation in perovskites was investigated by NMR and FTIR spectroscopy. First‐principles calculations further revealed that both insertion products are thermodynamically stable, aligning with experimental results. Additionally, X‐ray diffraction (XRD) and scanning electron microscopy (SEM) analyses demonstrated improved film quality in crosslinked perovskites, including enhanced crystallinity, reduced defect density, and suppression of metallic lead formation. Notably, the crosslinked PSCs exhibited intact gold electrodes, in stark contrast to control devices, which showed significant electrode cracking and structural degradation.

The bis‐diazirine‐functionalized PSCs achieved a certified PCE of 24.02%, surpassing conventional perovskite devices. Impressively, the modified PSCs retained 98.6% of their initial performance after 1000 h of continuous illumination, while control devices showed a 35% drop after just 200 h. These results demonstrate that covalent bonding with bis‐diazirine molecules provides a transformative approach to stabilizing PSCs and establishes diazirine crosslinkers as a powerful tool in perovskite photovoltaics. Beyond solar cells, this strategy also holds great promise for improving the stability and performance of other perovskite‐based optoelectronic devices, including LEDs and photodetectors.

### 4.4 Diazirines as Tools for Surface Modification

The reactivity of diazirine‐derived carbenes allows for the easy formation of covalent bonds with surfaces of different kinds, including nanoparticles (NPs) and nanostructured surfaces, eliminating the need for complex surface chemistry. This makes it a valuable tool for functionalizing surfaces of any size or shape and for creating patterns, which is useful for building sensors, electronic devices, and medical applications.

#### 4.4.1 Nanotubes and Particles

Ismaili et al. presented a nanohybrid material composed of multi‐walled carbon nanotubes (MWCNT) covalently bonded by diazirine‐based crosslinking to monolayer‐protected gold nanoparticles (AuNPs), which can potentially be used for tailoring NP surfaces, sensing, and nanomedicine.^[^ ^103^, ^104^, ^105^ ^]^ Furthermore, they showed the formation of diamond–gold nanojewel hybrids as well as AuNP modifications of graphene and glass employing diazirine crosslinking.^[^ ^104^ ^]^

Gobbo et al.^[^ ^106^ ^]^ described the functionalization of glassy carbon electrodes (GCEs) with gold nanoparticles (NPs) using diazirines. The GCE could be functionalized with good surface coverage by dipping the slide in a diazirine‐AuNP solution or drop‐casting the diazirine‐AuNP directly onto the surface. Such materials could be used for the manufacturing of conductive and patternable electrode surfaces.

**Figure 28:**
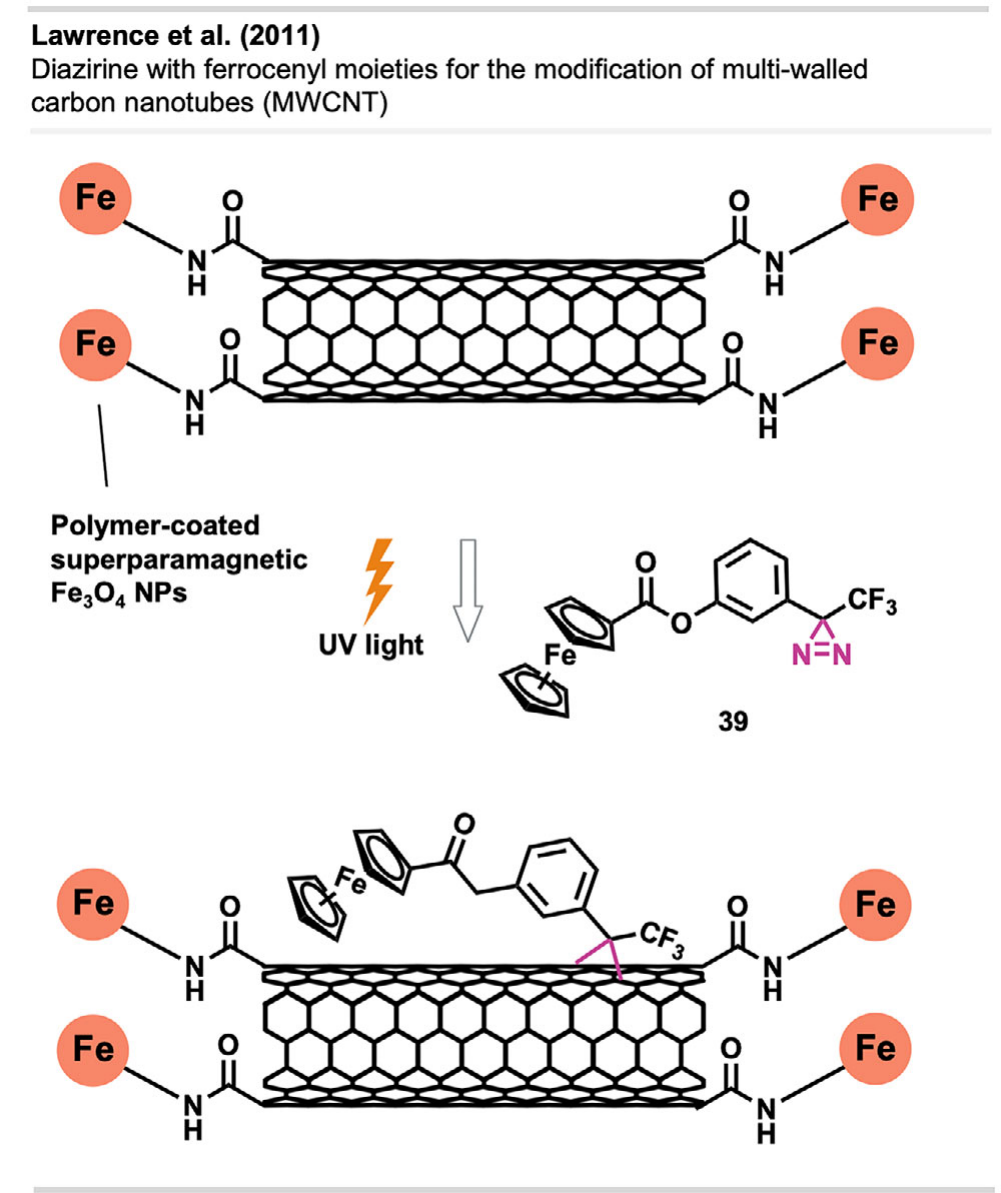
Covalent functionalization of magnetic MWCNTs with **39** through UV activation. Created with Biorender.com.

Lawrence et al.^[^ ^107^ ^]^ demonstrated the introduction of ferrocene groups on MWCNTs. In a related study, MWCNTs with polymer‐coated superparamagnetic Fe_3_O_4_ NPs attached to their ends were functionalized with diazirine **39** bearing a ferrocenyl moiety (Figure 28). This could be used to record stable non‐aqueous voltammetry even under vigorous conditions with a simple magnetic electrode design. This approach offers a wide range of possibilities for studying chemically modified CNT materials in non‐aqueous electrochemical applications, including electrocatalysis, batteries, and applications for electroanalysis.^[^ ^108^ ^]^ In a similar approach, Hesari et al. modified micro‐diamond surfaces^[^ ^109^ ^]^ and GCEs^[^ ^110^ ^]^ with redox‐active ferrocene moieties.

#### 4.4.2 Nanomedicine

Diazirine crosslinking has also been used in nanomedicine. There, polyethylene glycol is one of the most used stealth coatings for many types of nanomaterials, such as NPs, micelles, liposomes, polymeric NPs, or carbon nanotubes (CNTs). This hydrophilic layer avoids nonspecific interactions with off‐target sites and enables stealth‐like behavior (i.e., the ability to evade immune detection and clearance, thus prolonging its circulation or residence time). “Stealth NPs” are already used in biomedical applications for drug delivery, diagnosis, detection, and treatment.^[^ ^111^, ^112^ ^]^

**Figure 29:**
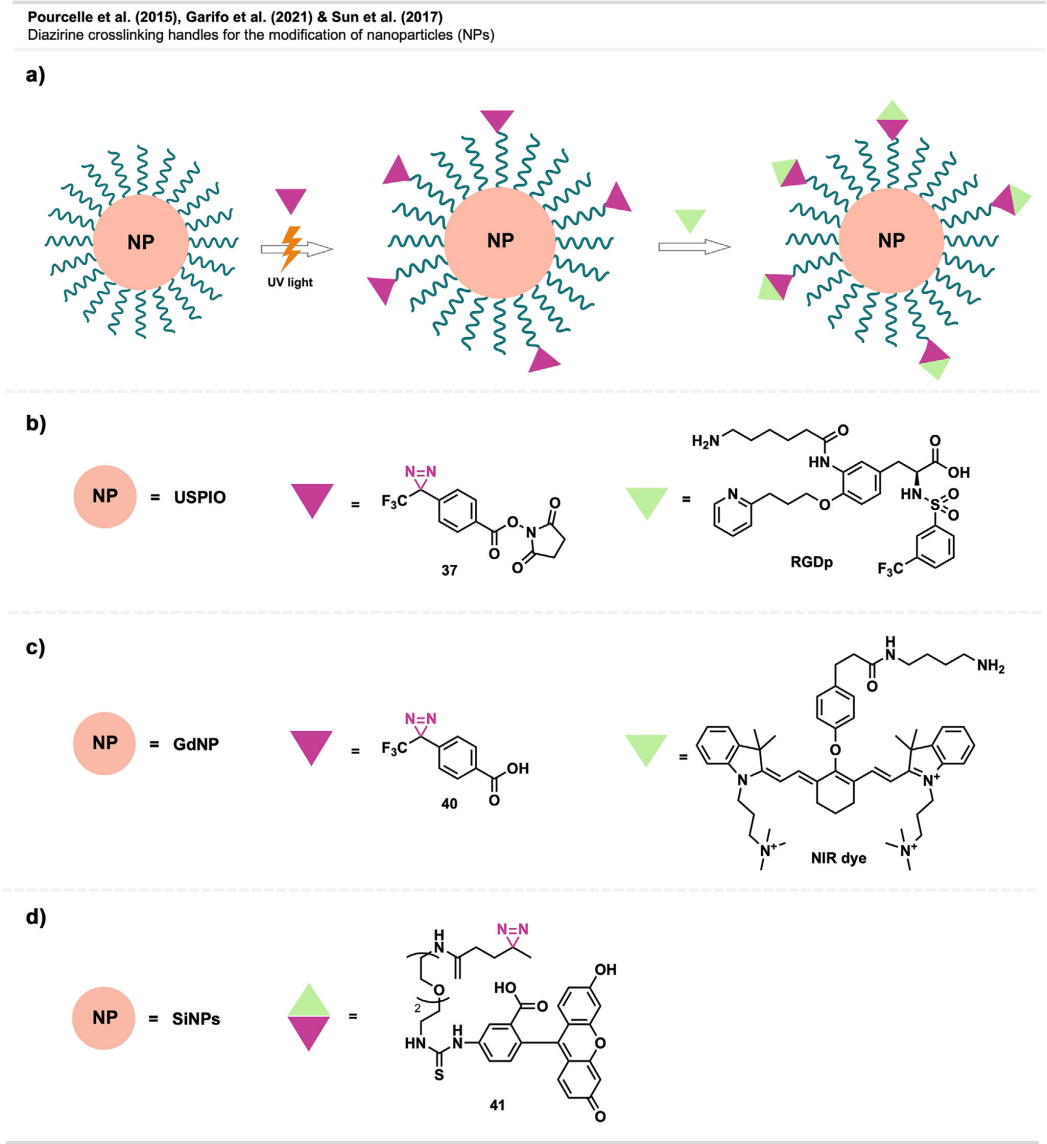
a) Schematic depiction of functionalization of PEGylated NPs. b) USPIO NPs modified with a peptide mimetic. c) Functionalization of GdNP with an NIR dye. d) Direct functionalization of silicon NPs with a fluorescent dye containing a diazirine linker. Created with Biorender.com.

To tune their ability to selectively interact with specific biomolecules, cells, or tissues, facile methods for modifying the unreactive PEG coatings are required. Diazirines have been successfully utilized for functionalizing PEGylated stealth NPs with potential use in targeted imaging technology in cancer therapy (Figure 29a).

Pourcelle et al.^[^ ^113^ ^]^ reported a robust and efficient method to graft molecular probes onto PEG chains of NP coronas in an aqueous environment. The work showed the successful functionalization of PEGylated ultrasmall superparamagnetic iron oxide (USPIO) NPs, a magnetic resonance imaging (MRI) agent, with an RGD peptide and other peptidomimetics. The molecular probes were introduced to the PEG surface by a bireactive linker bearing a diazirine group on one side and an NHS‐ester on the other (Figure 29b). The PEGylated NPs with specific targeting ligands showed good stealth properties in in vitro experiments and increased labeling of cells expressing the targeted integrins (a marker for certain cancer cells).

Recently, Garifo et al.^[^ ^114^ ^]^ reported another application of diazirine‐mediated functionalization of NPs used for sensitive MRI applications and optical imaging (Figure 29c). The work showed the development of a bimodal probe bearing both a paramagnetic Gd^3+^ complex inside the NP core and a near‐infrared luminescent dye (NIR‐dye) on the outer coating corona. The Gd‐HP‐DO3A chelate was encapsulated in PEG‐coated silicon NPs, followed by the photochemical introduction of a diazirine clip bearing a carboxy group. The NIR probe was, in turn, grafted onto the carboxylated SiO_2_‐Gd‐NPs using carbodiimide chemistry.^[^ ^115^ ^]^ The modified NP imaging agent was evaluated in vivo using small animal MRI and fluorescence optical imaging techniques.

Sun et al.^[^ ^116^ ^]^ reported a similar approach for the fluorescent labeling of silicon nanoparticles (SiNPs) with direct UV‐induced attachment of **40** for potential bioimaging applications using an aliphatic diazirine linker (Figure 29d).

As discussed in the first chapter, applications of diazirines in drug discovery have remained scarce, aside from PAL, primarily due to the lack of a practical method for activating diazirines in vivo. A recent and transformative approach reported by Zhu et al. is poised to change this paradigm.^[^ ^117^ ^]^

**Figure 30:**
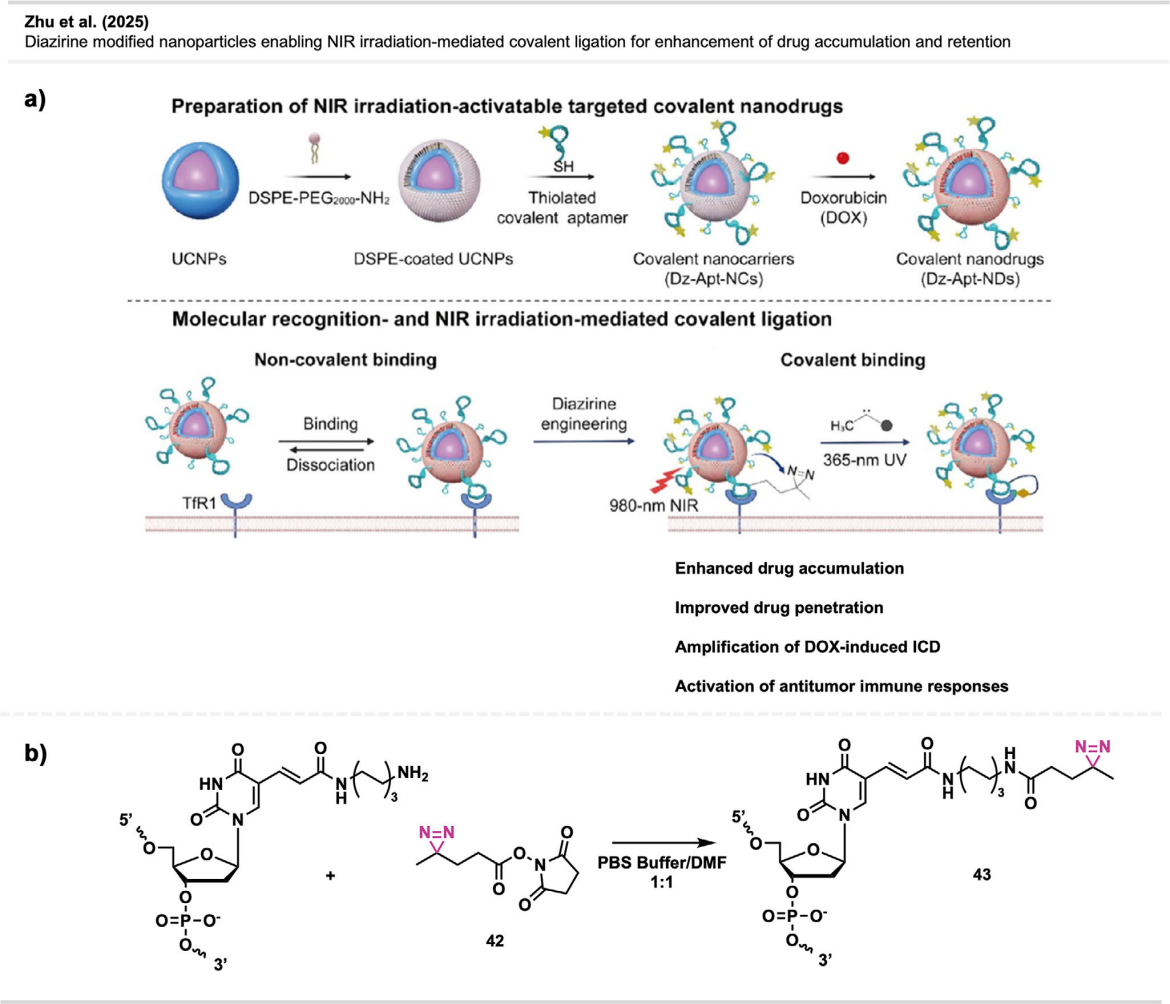
a) Upper panel: Schematic illustration of an NIR light‐activated targeted covalent nanodrug (Dz‐Apt‐NDs), constructed by grafting diazirine‐functionalized aptamers onto PEGylated phospholipid‐coated upconversion nanoparticles (UCNPs), followed by loading of the chemotherapeutic drug DOX. Lower panel: Compared to conventional targeted nanodrugs, targeted covalent nanodrugs combine molecular recognition, diazirine reactivity, and light‐controlled precision, enabling covalent binding to target proteins through proximity‐driven crosslinking activated by NIR irradiation. Targeted covalent nanodrugs significantly enhanced the accumulation, retention, and penetration of chemotherapeutics within tumors, thereby amplifying DOX‐induced immunogenic cell death (ICD), reactivating T cells, and stimulating antitumor immune responses in immunosuppressive 4T1 tumors. b) Synthesis of diazirine aptamer **42**. Section a is a direct reproduction with minor additions from Zhu et al.,^[^ ^117^ ^]^ permission acquired via RightsLink. © 2025 American Chemical Society.

The study presented a light‐activatable nanodrug platform that harnesses diazirine chemistry to achieve covalent binding to tumor cells (Figure 30a). The system was built around upconverting nanoparticles (UCNPs), which show photon upconversion by absorbing low‐energy near‐infrared (NIR) light and emitting higher‐energy ultraviolet or visible light, enabling deep‐tissue photoactivation in vivo. UCNPs are coated with amphiphilic DSPE‐PEG and functionalized with DNA aptamers (short, single‐stranded DNA) targeting transferrin receptor 1 (TfR1), a protein commonly overexpressed in cancer cells. The aptamers are modified with an NHS‐diazirine reagent **42**, which introduces a diazirine moiety via amine coupling to a thymidine base to give **43** (Figure 30b). Thiolated aptamers were then coated onto DSPE‐PEG2000‐NH_2_‐coated UCNPs using the heterobifunctional crosslinker sulfo‐SMCC to form covalent nanocarriers (Dz‐Apt‐NCs), which were subsequently loaded with the chemotherapeutic drug doxorubicin (DOX). Upon irradiation with 980 nm NIR light, the UCNP core emits localized UV light, triggering the photolysis of the diazirine group. Carbene activation created a covalent link between the NP and TfR1 on the cell surface. This light‐induced, proximity‐driven anchoring mechanism enabled precise, durable attachment of the NP to the tumor.

In vivo tests showed that the diazirine‐functionalized NPs lead to suppressed tumor growth and prolonged survival in mice. Notably, treated tumors displayed increased immune infiltration, suggesting the platform not only kills cancer cells but also stimulates antitumor immunity.

This work marks a conceptual leap in diazirine chemistry, demonstrating a feasible and highly controlled in vivo activation strategy for the first time. It opens the door to a new class of targeted covalent nanotherapeutics and illustrates a creative way to unlock the long potential of diazirines in drug discovery beyond in vitro PAL.

#### 4.4.3 Topical Crosslinking

Several applications of diazirines involving topical crosslinking have been reported.

**Figure 31:**
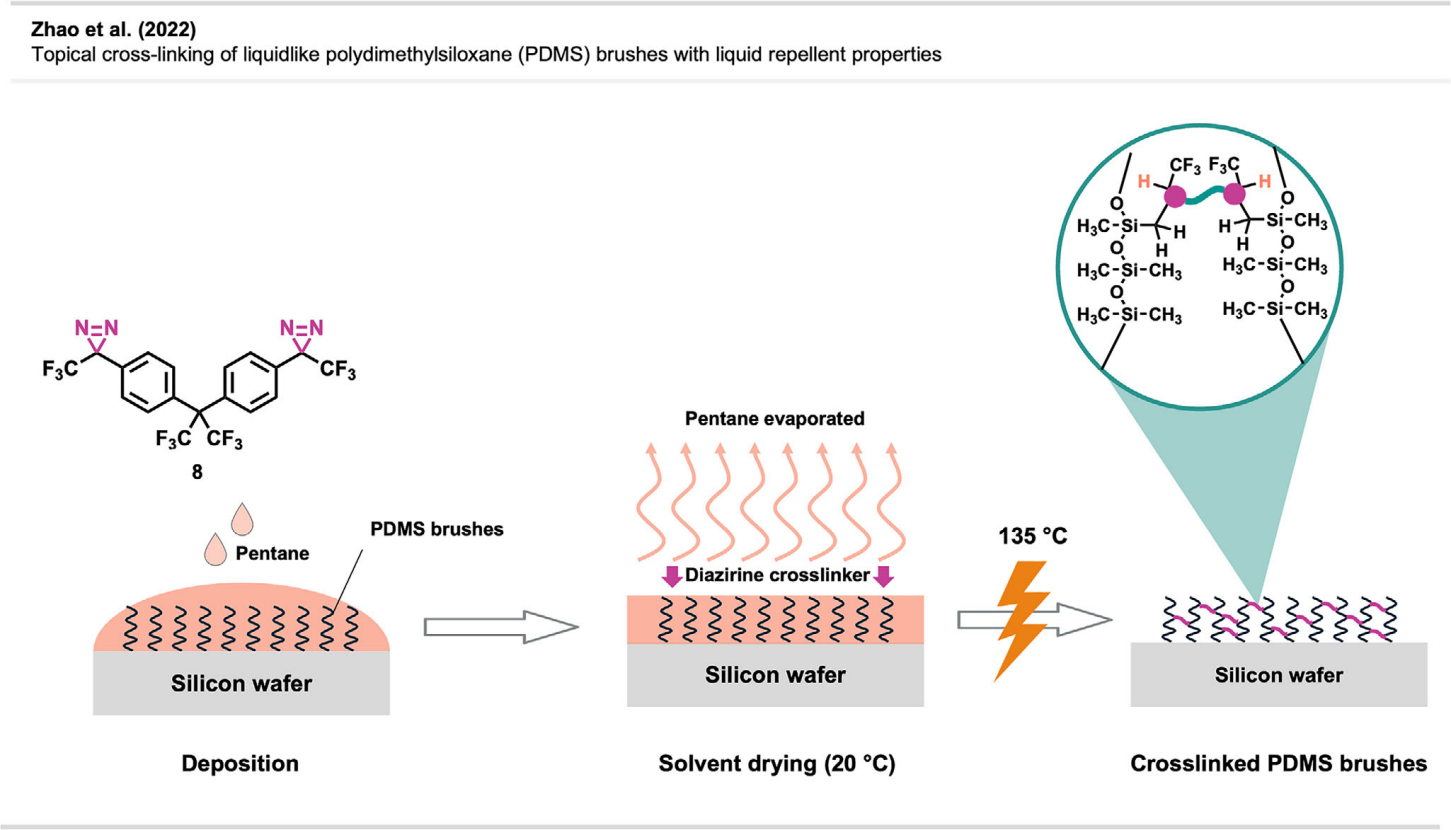
Overview of topical crosslinking of PDMS brushes immobilized to a silicon wafer with bis‐diazirine **8** upon thermal activation.

In a study by Zhao et al.,^[^ ^118^ ^]^ liquidlike PDMS brushes immobilized to a solid surface (silicon wafers, polycarbonate) were thermally crosslinked via carbene insertion using bis‐diazirine **8** (Figure 31). These brushes are assemblies of about 10 nm wide linear siloxane chains, behaving like untethered liquid lubricants due to their ability to move their non‐immobilized chain ends. Crosslinking these chains with diazirine linkers enhanced mechanical durability while their liquid repellent properties were conserved. The crosslinked brushes can be used on surfaces requiring optical transparency, durability, and liquid repellent properties, such as face shields or several other medical applications.

**Figure 32:**
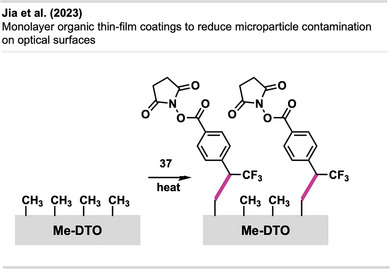
Surface modification of silicon substrate DTO with an NHS diazirine.

Jia et al.^[^ ^119^ ^]^ developed monolayer organic thin‐film coatings to reduce microparticle contamination on optical surfaces. One of the tested coatings included carbene‐based diazirine precursor **37**, applied via vapor‐phase deposition and UV activation (Figure 32). This coating enabled covalent grafting onto fused‐silica damage test optics (DTO), high‐purity silica substrates used for testing, via C─H insertion. This resulted in robust and stable surface layers. The NHS ester remained intact during carbene‐mediated anchoring and showed stability through the experiments. However, it could also be used as a reactive handle for post‐crosslinking surface functionalization (e.g., coupling to amines). The coatings improved particle removal, maintained laser‐induced damage threshold (LIDT) performance, and showed long‐term stability, marking the first demonstration of carbene‐functionalized surfaces under laser‐relevant conditions.

**Figure 33:**
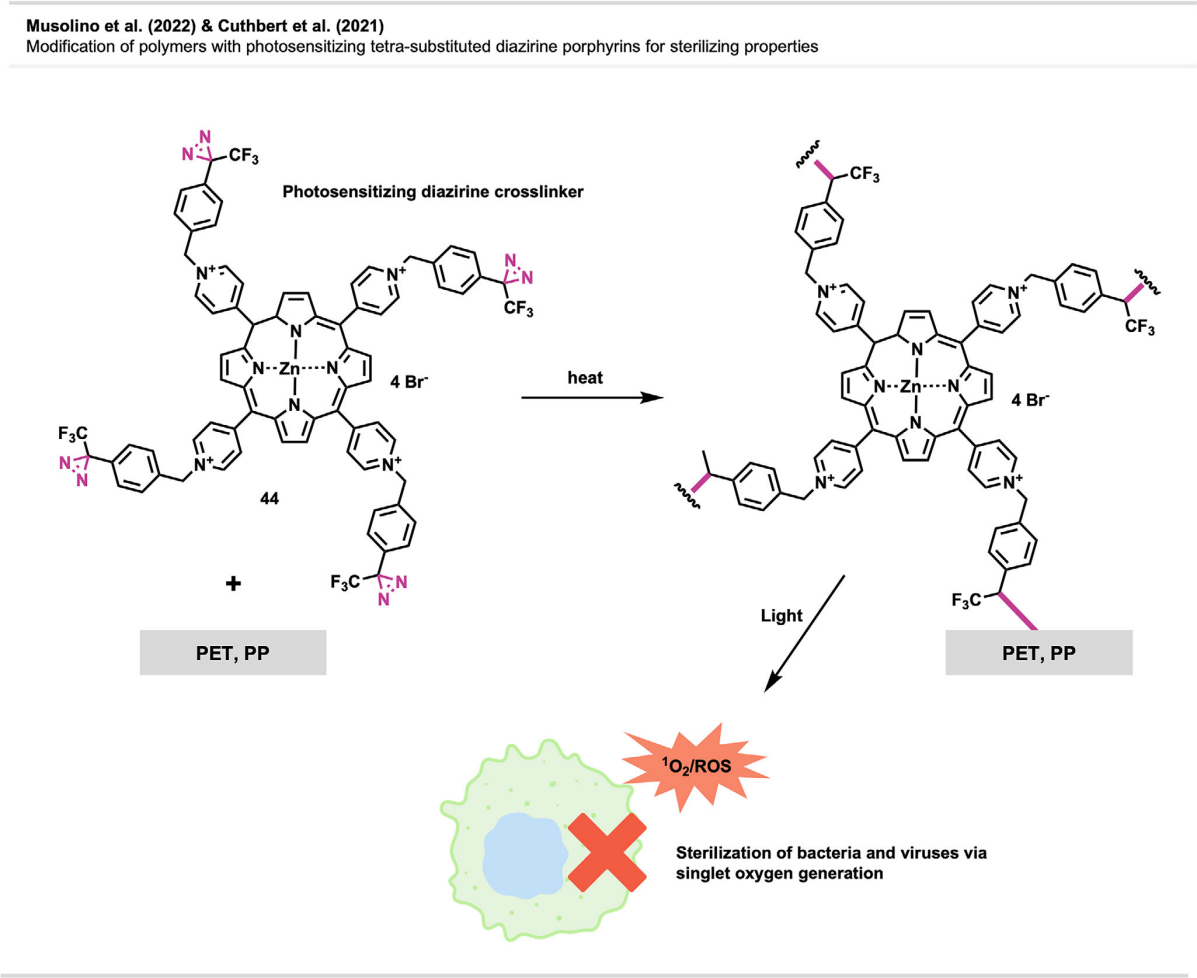
Modification of a PET surface with **44**. Upon light activation, the crosslinked photosensitizing tetra‐substituted diazirine porphyrin produces ^1^O_2_/ROS at the material's surface, leading to pathogen inactivation. Created with BioRender.com.

Diazirine‐mediated crosslinking has also been used to modify polymer surfaces with antimicrobial features. In a study by Musolino et al.,^[^ ^54^ ^]^ photosensitizing tetra‐substituted diazirine porphyrins **44** were crosslinked to a PET surface via carbene insertion. The antimicrobial activity of these applications is based on the production of reactive oxygen species (ROS), such as singlet oxygen (^1^O_2_), by the porphyrins upon light activation (Figure 33).

Experiments showed 97% inhibition of bacterial growth (*Staphylococcus aureus*) after 6 h of irradiation. In a similar study by Cuthbert et al.,^[^ ^120^ ^]^ polypropylene non‐woven filter material—commonly used in personal protective equipment such as N95 masks—was functionalized with a diazirine‐linked photosensitizer. Upon 4 h of visible light exposure, the material achieved 99.99% inactivation of the influenza A virus, demonstrating near‐complete sterilization via singlet oxygen generation. This approach offers a promising route toward self‐sterilizing, reusable protective equipment.

**Figure 34:**
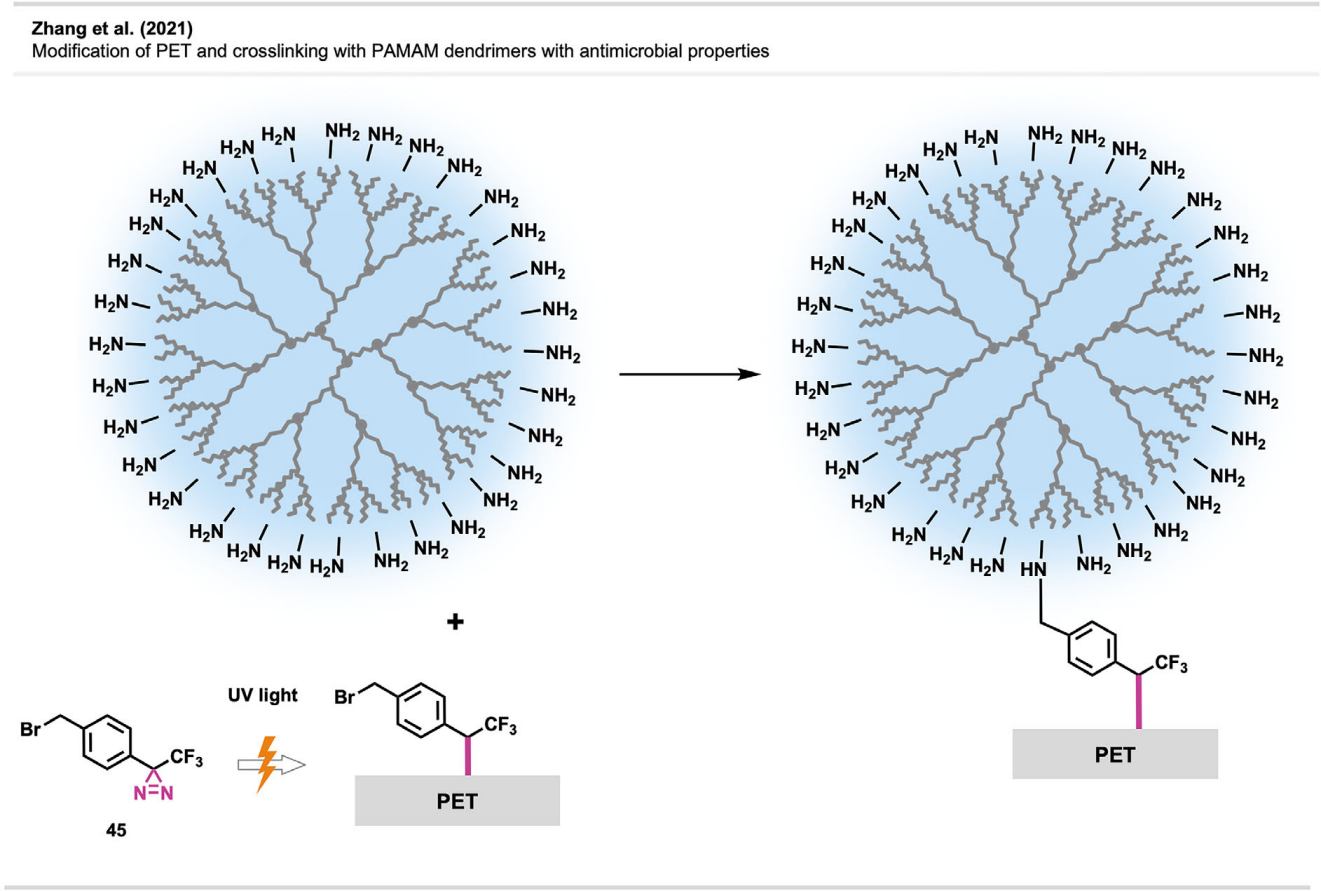
Modification of a PET surface via crosslinking of **45** followed by substitution with PAMAM dendrimer. Redrawn and adapted from Zhang et al.,^[^ ^121^ ^]^ permission acquired via RightsLink. © 2021 Elsevier. Created with Biorender.com.

Zhang et al.^[^ ^121^ ^]^ reported the modification of a PET surface with PAMAM dendrimers (discussed in detail in Section 4.5). The branched polymers exhibited antibacterial properties through electrostatic interactions of the positively charged chain ends with prokaryotic cell membranes. Compound **45** was covalently bound to the PET surface, leaving the benzyl bromide moiety to subsequently participate in nucleophilic substitution reactions with the primary amines of the PAMAM dendrimers. (Figure 34) The PAMAM graft was able to kill gram‐positive and gram‐negative bacteria without influencing the bulk properties of the materials.

#### 4.4.4 Dyeing

**Figure 35:**
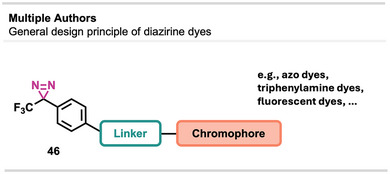
Generic structure of a diazirine dye.

Recently, diazirine crosslinking has also been used to dye various polymer materials for garments. Many synthetic fibers are colorless due to their lack of a suitable π‐conjugated system and therefore must often be dyed before garment fabrication. This is especially challenging for low‐energy surface materials. Moreover, traditional dyeing methods can be expensive, have poor color fastness, and can negatively affect the material's properties. Hence, crosslinking strategies that utilize diazirine‐based dyes—exemplified by **46** (Figure 35)—offer a promising solution for dyeing synthetic fibers.

Jiang^[^ ^122^ ^]^ and Guo^[^ ^123^ ^]^ developed azo chromophores containing diazirine moieties for the coloration of synthetic fibers. Deep dyeing performances were achieved on PET, nylon, PP, aramid, acrylic, and polyurethane (spandex) fibers. The dyed materials showed good color fastness without significantly altering their mechanical properties.

Wang et al.^[^ ^124^ ^]^ introduced three novel diazirine‐based carbene dyes designed for covalent dyeing of synthetic fabrics via thermally triggered C─H bond insertion. The dyes exhibited excellent dye fixation rates (up to 74%) and fastness properties on polyester, polypropylene, spandex, aramid, and polyester/spandex blends using a non‐aqueous methanol‐based process without additives. The dyes maintained vibrant color strength, superior leveling, and reusability of solvents and dyes. This work offers a promising strategy for environmentally friendly, additive‐free, and broadly applicable fiber dyeing using diazirine chemistry, with the potential to significantly reduce water pollution in the textile industry.

**Figure 36:**
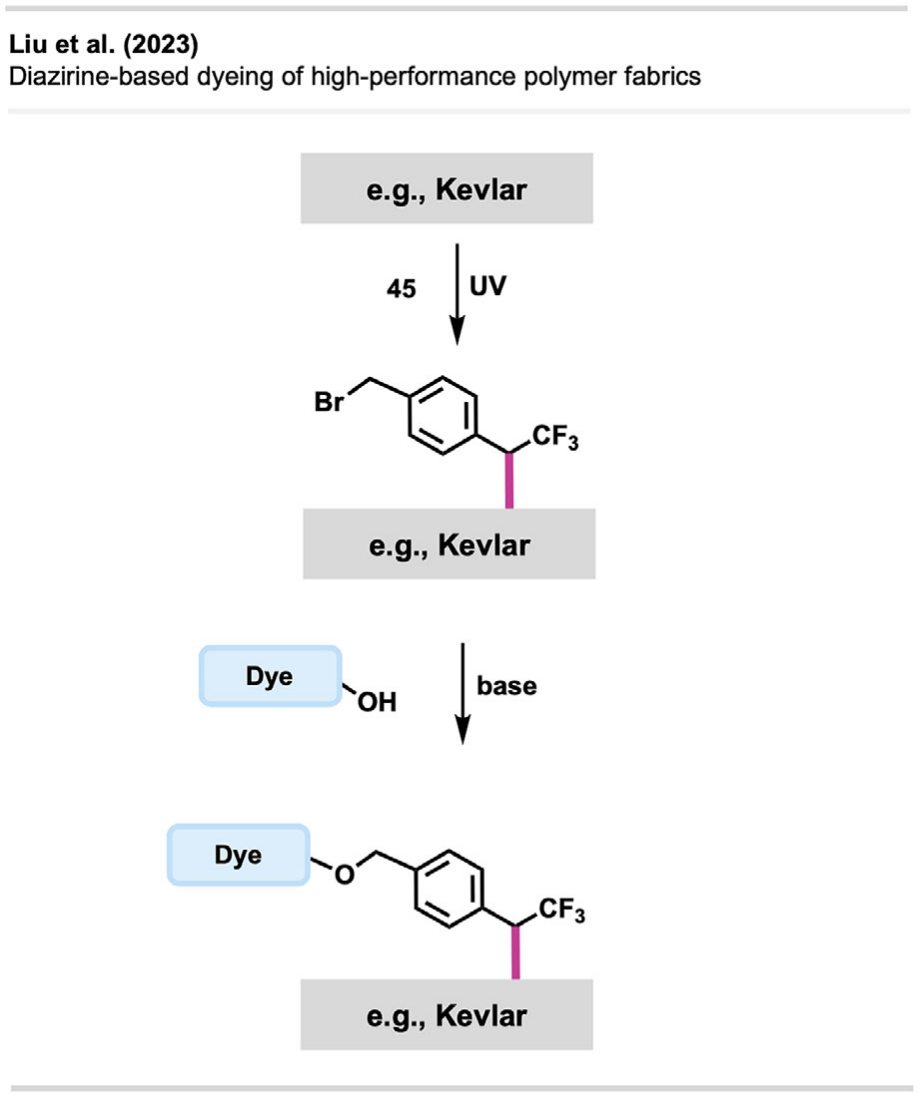
Carbene‐mediated dyeing of Kevlar using crosslinker **45**. Redrawn and adapted from Liu et al.^[^ ^125^ ^]^ Licensed under CC BY. © 2023 Royal Society of Chemistry.

Liu et al.^[^ ^125^ ^]^ introduced a method for dyeing fabric made of high‐performance polymers, including para‐aramid (Kevlar), UHMWPE (Spectra or Dyneema), and nylon (Cordura), which are traditionally challenging to dye. This process utilized photoactivation to attach bifunctional diazirine **45,** modifying the surface with benzyl bromide moieties that served as a platform for connecting various nucleophilic dye molecules through nucleophilic substitution (Figure 36). The different fabrics were dyed either in a solution or by applying a dye solution containing a base onto the pre‐functionalized fabric surface, followed by washing. In performance tests, the dyed fabrics exhibited colorfastness under simulated laundering, retained thermal stability, and preserved their mechanical properties. Compared to conventional approaches limited to monochromatic dyeing, this approach holds the potential for dyeing ballistic fabric in multiple colors, such as camouflage patterns.

**Figure 37:**
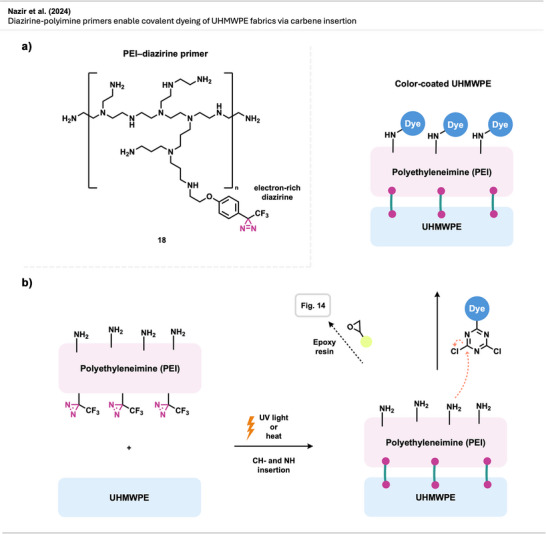
Diazirine‐polyamine primers enable covalent dyeing of UHMWPE fabrics. a) Structure of the electron‐rich PEI‐diazirine primer **18**. b) Schematic representation of dye attachment to the PEI‐modified UHMWPE surface. Upon UV or thermal activation, carbene insertion into C─H and N─H bonds anchors PEI onto UHMWPE, leaving surface amines available for covalent dye coupling.

In addition to the epoxy surface modification discussed in Figure 14, Nazir et al. demonstrated that PEI‐diazirine conjugates also enable covalent dyeing of UHMWPE fabrics without the need for oxidative pretreatment (Figure 37). Upon thermal or photochemical activation, the diazirine groups generate carbenes that insert into C─H bonds of the polyethylene surface and induce N─H insertion or self‐crosslinking within the PEI network, forming a stable, reactive coating capable of anchoring dye molecules. The surface‐exposed NH_2_ groups allow direct covalent attachment of chromophores to the PEI layer. The electron‐rich PEI‐diazirine conjugate **18** showed the highest surface retention and dye uptake, resulting in durable, wash‐resistant coloration. Spectroscopic and microscopic analyses confirmed successful covalent grafting and surface modification, highlighting this strategy as an effective and mild approach for introducing functional dyes onto otherwise inert polyolefin fabrics.

#### 4.4.5 Immobilization of Proteins and Analytes

The final section of this chapter discusses surface modifications, where diazirine crosslinking has been employed to anchor small molecules as well as proteins and antibodies to surfaces and affinity beads for screening applications,^[^ ^126^, ^127^, ^128^, ^129^ ^]^ thereby bridging over to traditional photoaffinity applications. Several methods for target identification of this kind have already been reviewed by Kanoh, who focused on photo‐crosslinked affinity matrices for small‐molecule target identification and covered literature up to 2015.^[^ ^130^ ^]^ More recent developments have expanded the scope of diazirine‐based crosslinking to include diverse surface functionalization and spatially resolved protein immobilization strategies, which are discussed here.

**Figure 38:**
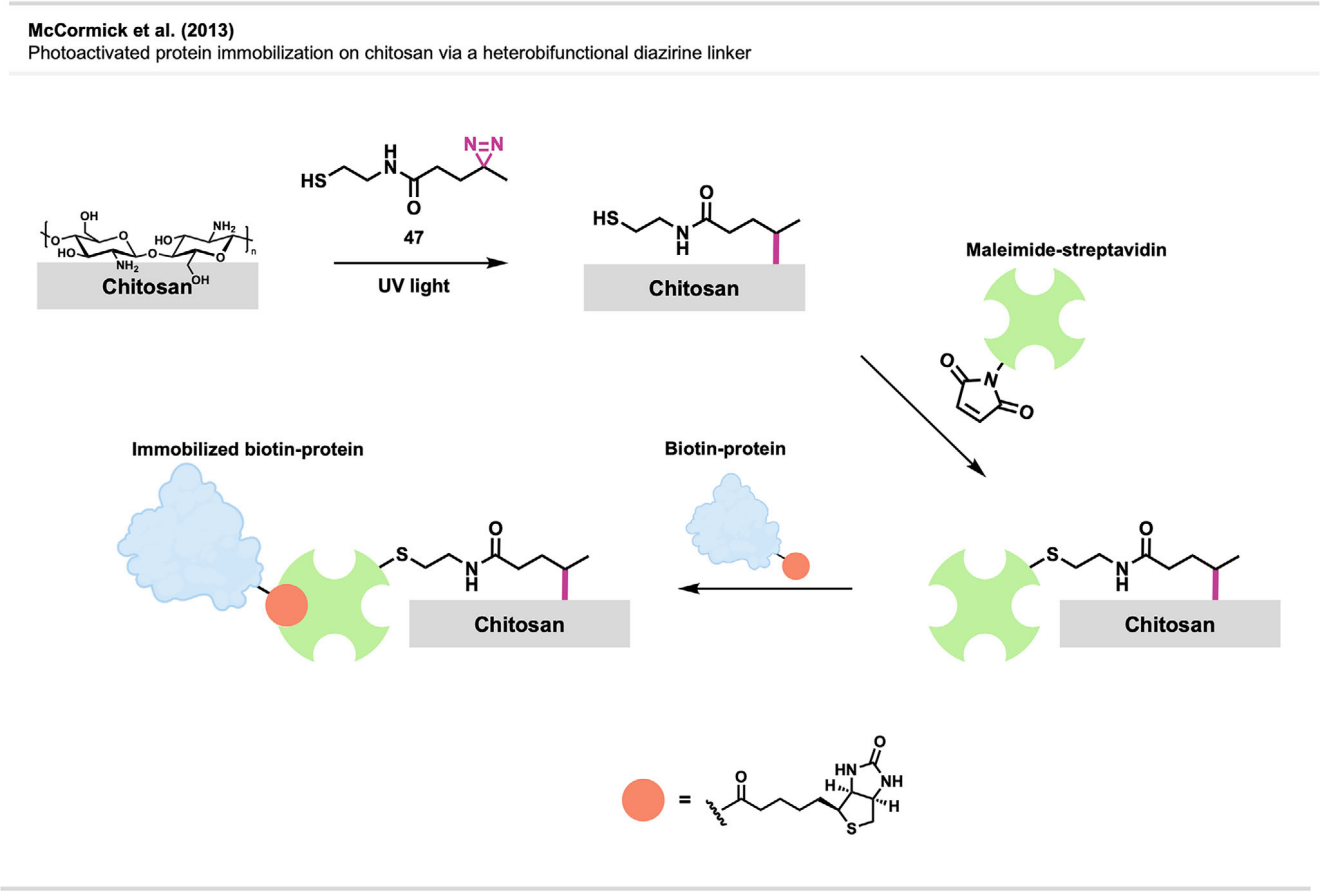
Modification of a chitosan film with diazirine **47**, followed by coupling to streptavidin and immobilization of a biotin‐conjugated protein. Created with BioRender.com.

McCormick et al.^[^ ^131^ ^]^ were the first to report photoactivated immobilization using diazirines, revealing the method's potential and transformative capabilities. The researchers synthesized the heterobifunctional crosslinker **47**, which was then photolytically attached to the primary amines of a chitosan film (Figure 38). Subsequently, this modified film was conjugated to a maleimide‐containing protein or biomolecule, providing spatial control over the location and presentation of the conjugated entities for further attachments. In their study, streptavidin was initially attached to chitosan using the N‐MCEP‐diazirine linker, followed by the attachment of biotinylated fusion proteins nerve growth factor (NGF) and Semaphorin3A (Sema3A) to investigate neurite growth. This work effectively showed the potential of the immobilization strategy, which later demonstrated its translatability and applicability to a diverse range of materials.

**Figure 39:**
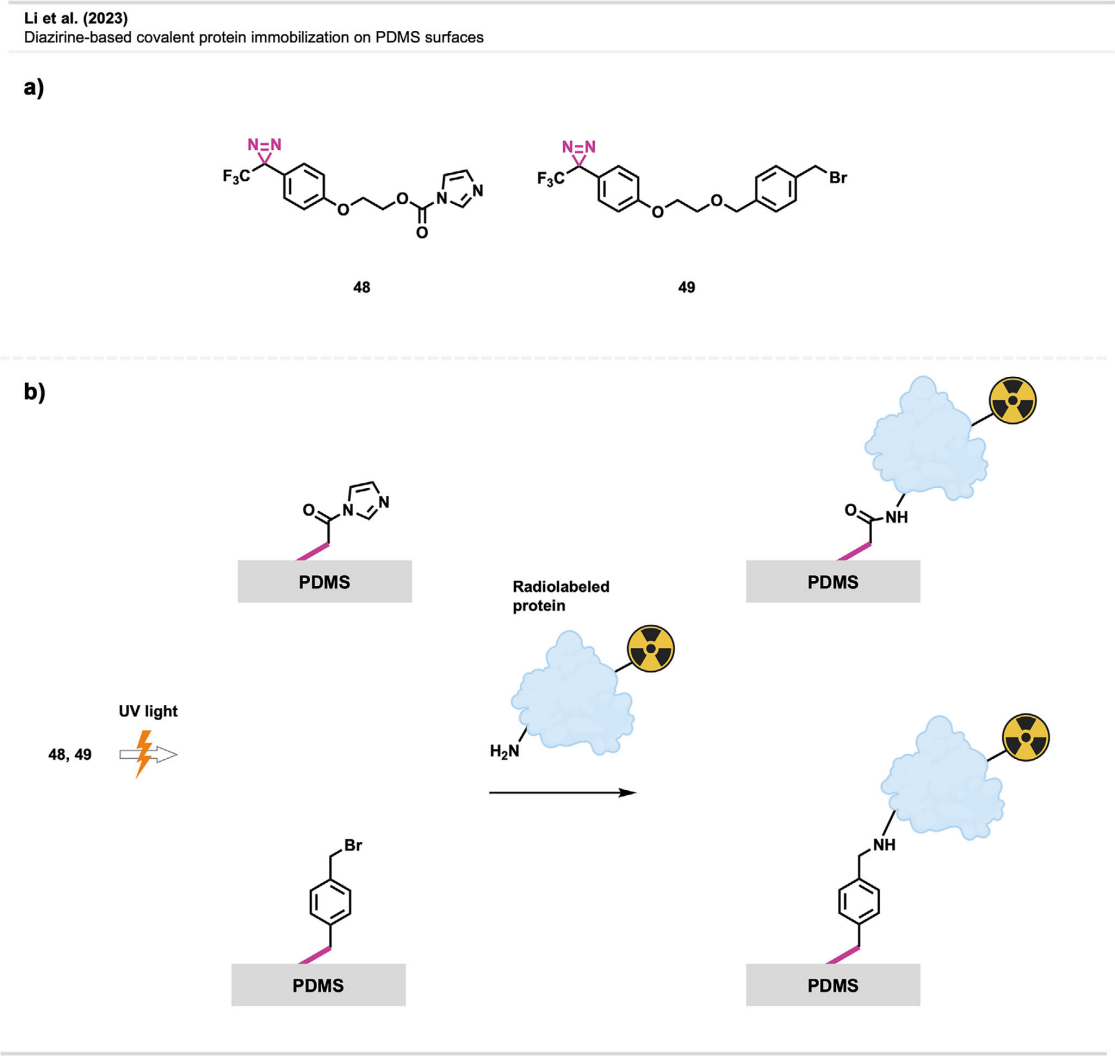
a) Diazirine linkers **48** and **49** with reactive functional group handles. b) Modification of the PDMS surface via carbene insertion (not shown). Proteins can react with the reactive groups and immobilize on the PDMS surface. Proteins can be equipped with detection markers (e.g., radioisotopes). Redrawn and adapted from Li et al.,^[^ ^132^ ^]^ permission acquired via RightsLink. © 2021 Elsevier Created with BioRender.com.

Based on the earlier discussed study, where PDMS brushes were modified by crosslinking,^[^ ^118^ ^]^ the same group developed an efficient and scalable method for covalently immobilizing proteins onto PDMS using diazirine‐based small‐molecule linkers (Figure 39). Two types of mono‐diazirine compounds were designed bearing the electrophilic groups imidazole carbamate (imidazole carbamate diazirine **48**) and benzyl bromide (benzyl bromide diazirine **49**). First, the compounds were covalently linked by carbene‐mediated C─H insertion with the PDMS substrate by protein conjugation via nucleophilic substitution. The diazirine‐based protein immobilization was investigated using BSA and immunoglobulin G (IgG) as model biomolecules. Quantification of BSA and IgG immobilization on diazirine‐modified PDMS surfaces was conducted through iodine‐125 radiolabeling, and the stability of immobilized proteins was evaluated using sodium dodecyl sulfate (SDS) elution. Moreover, confocal microscopy was employed to show the distribution of immobilized IgG on the modified PDMS surface. This surface modification strategy demonstrates an innovative avenue for polymeric surface functionalization, aiming to enhance protein immobilization for diverse biomedical applications such as biosensors, biomaterials, and microfluidics.^[^ ^132^ ^]^

**Figure 40:**
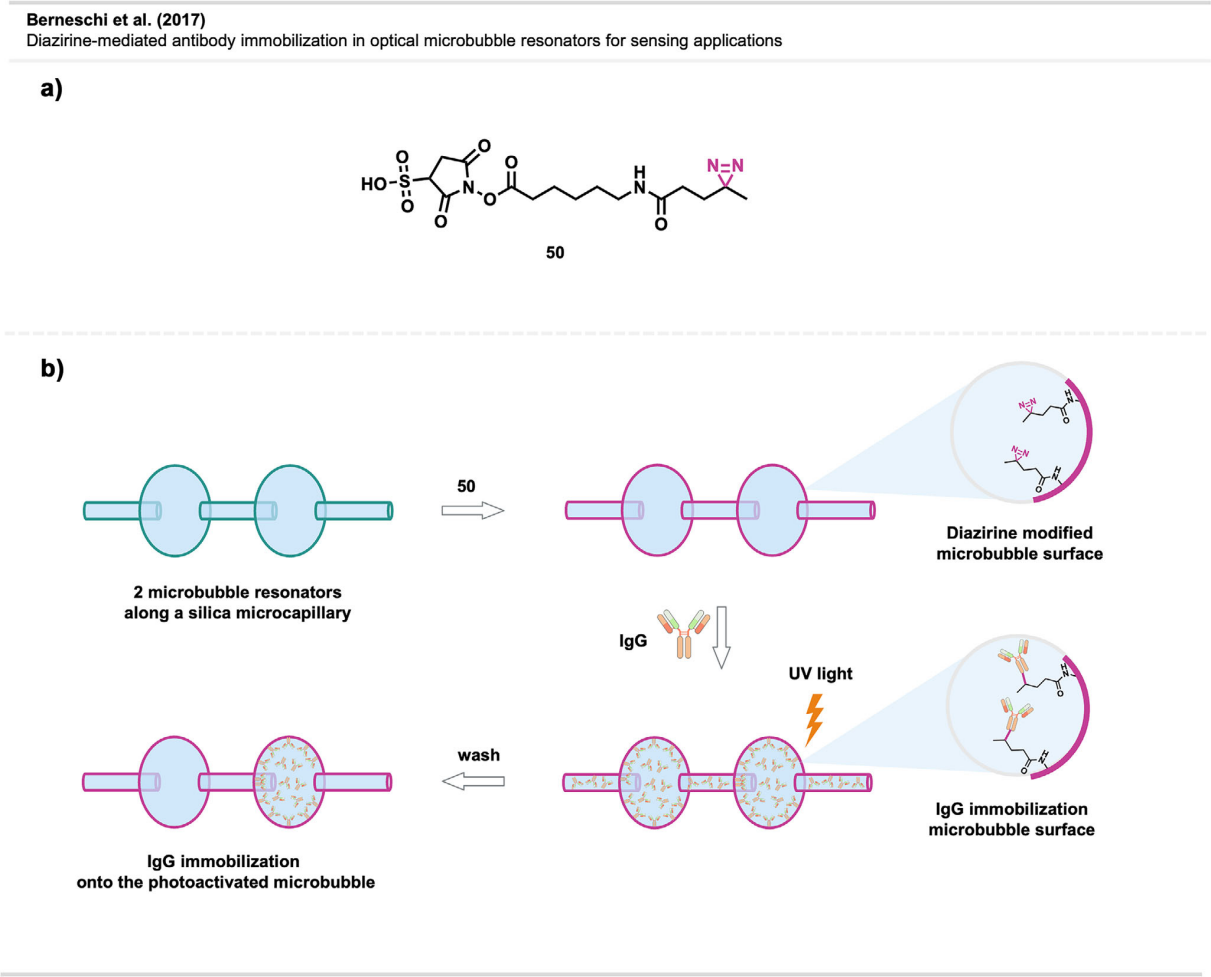
a) Structure of **50**. b) Schematic of IgG immobilization onto optofluidic microbubble resonators (OMBRs) using diazirine chemistry. Complete functionalization workflow: Modified silica microcapillaries are functionalized with **50**. Upon 365 nm UV activation, the diazirine forms a reactive carbene that covalently attaches IgG antibodies to the microbubble. Principle of OMBR‐based biosensing: as analytes (e.g., antigens) flow through the microbubble and bind to the immobilized IgG, the local refractive index changes, inducing a measurable shift in the whispering gallery mode (WGM) resonance wavelength. The magnitude of this shift directly correlates with the binding event, enabling real‐time, label‐free detection. Redrawn and adapted from Berneschi et al.,^[^ ^133^ ^]^ permission acquired via RightsLink. © 2017 Elsevier. Created with BioRender.com.

Berneschi et al. described a technique for attaching antibodies to the inner surface of optical microbubble resonators (OMBRs) using diazirines and UV light for immunoassays. These optical microresonators are highly sensitive physical, chemical, and biological sensors constructed from silica with dimensions in the range of a few hundred micrometers in diameter and a micrometer‐scale wall thickness. NHS‐diazirine **50** was linked into the microbubble of the silica microcapillary, which could then immobilize proteins or antibodies such as IgG upon UV activation. The novel diazirine‐based method is spatially selective and enables the creation of various sensing sites on the same glass capillary, and hence, shows the potential for the development of biochemical detection systems (Figure 40).^[^ ^133^ ^]^

**Figure 41:**
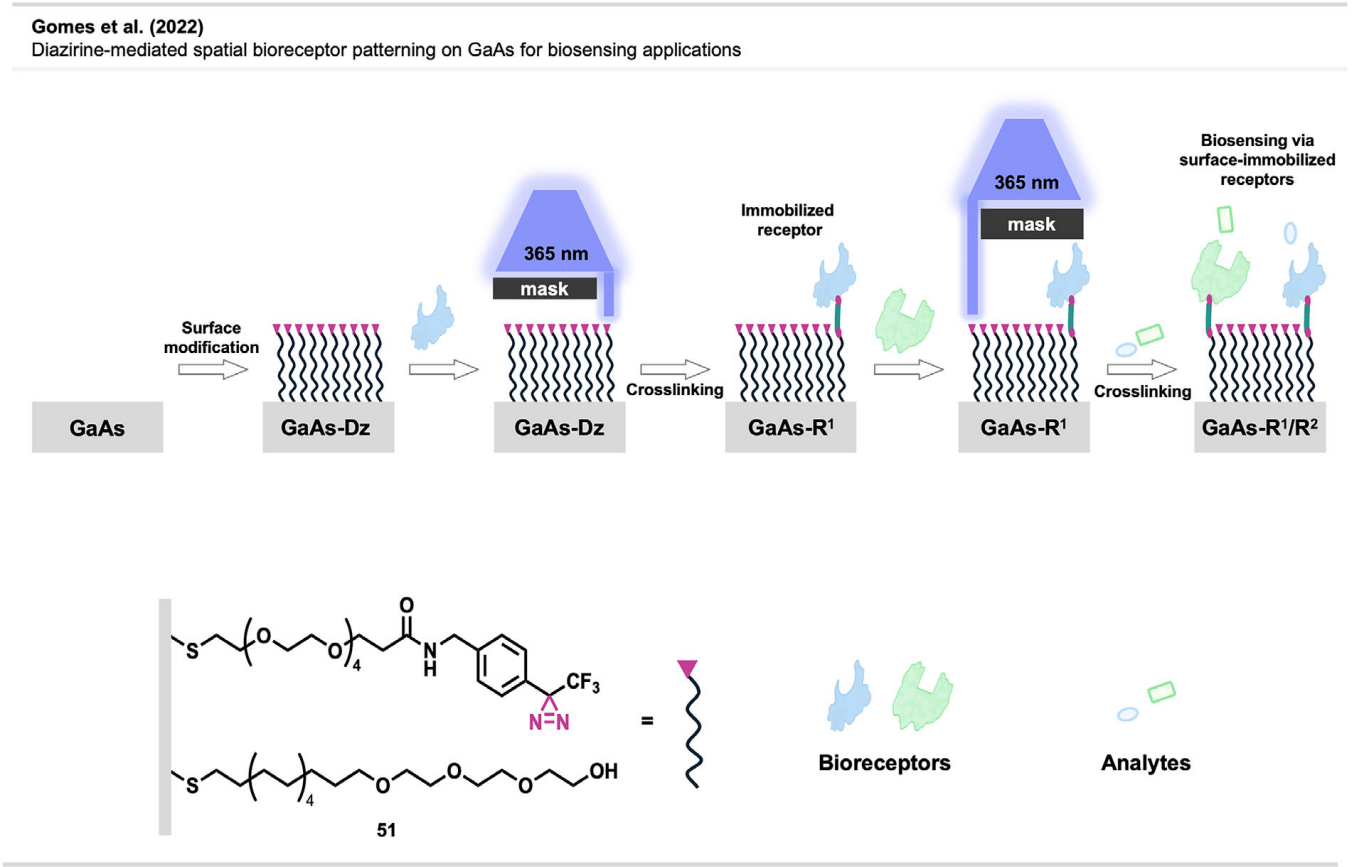
Schematic depiction of a modified GaAs surface. First, the diazirine‐modified monolayer **51** is formed. The diazirine groups are activated during photopatterning to immobilize distinct bioreceptors with high spatial resolution for biosensing applications. Redrawn and adapted from Gomes and Masia,^[^ ^134^ ^]^ licensed under CC BY. © 2022 Royal Society of Chemistry Created with BioRender.com.

In a similar approach, Gomes and Masia^[^ ^134^ ^]^ used diazirine crosslinking to selectively attach multiple bioreceptors to a gallium arsenide (GaAs) surface for potential biosensing applications. As a proof of concept, they successfully attached two proteins, neutravidin and endo‐sulfine alpha protein, to the GaAs surface at different locations. To achieve this, GaAs was initially functionalized using PEG thiol acid self‐assembled monolayers (SAMs) that take advantage of the highly covalent As–S bond. The crosslinker was then attached using carbodiimide chemistry to give functionalized monolayer **51**, while the unfunctionalized hydroxy chains serve as spacers. The diazirines could then be activated under UV light and spatially crosslinked to biomolecules (Figure 41). This study showed that using standard lithography techniques, the method can be easily adapted to immobilize multiple bioreceptors with high spatial resolution.

**Figure 42:**
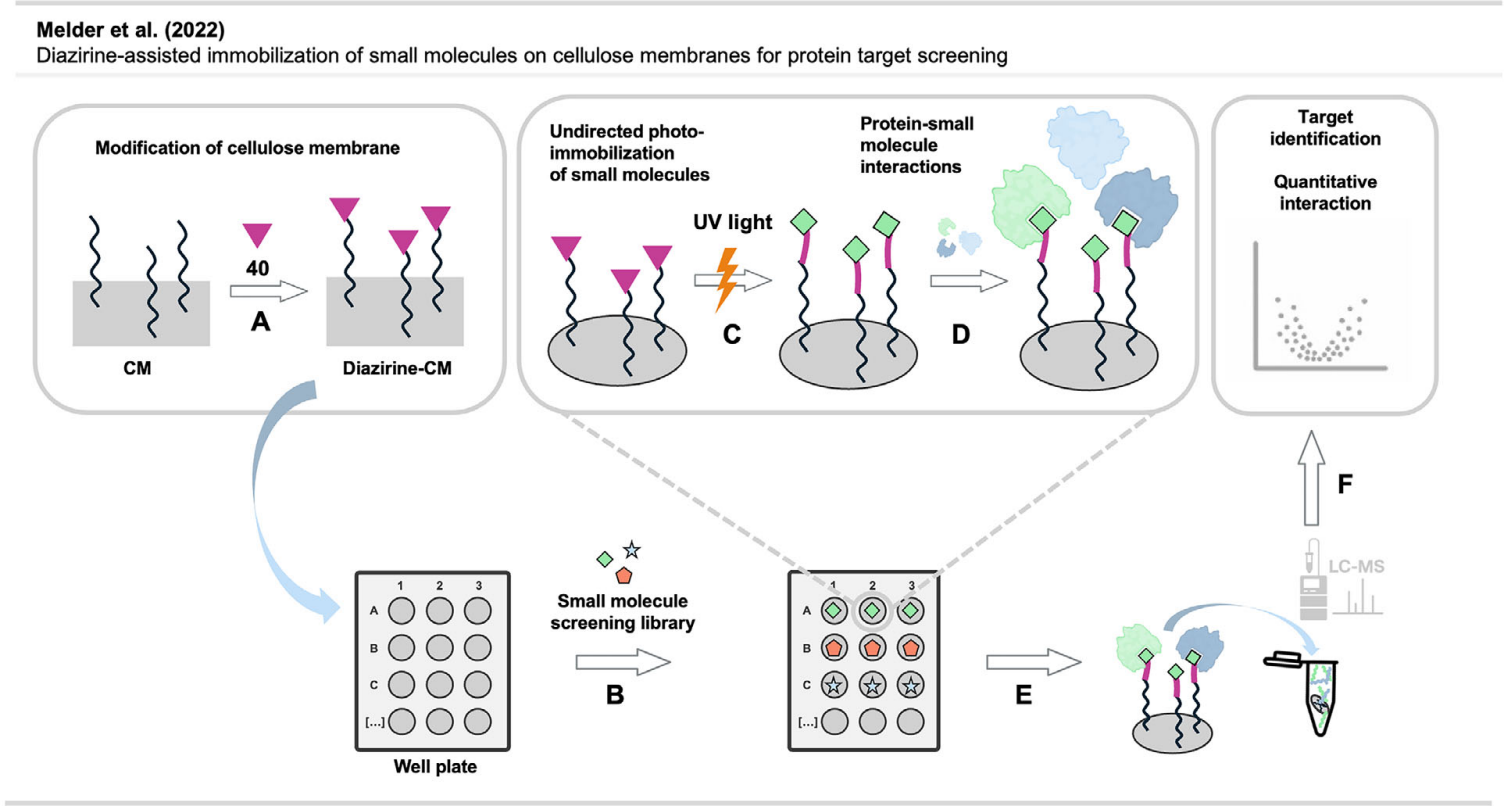
a) A photoactive cellulose membrane is prepared, b) followed by selection of small molecules from a screening library. c) Upon UV activation, diazirine groups generate reactive carbenes that covalently attach small molecules to the membrane via undirected crosslinking. d) The membrane is incubated with whole cell extracts to capture protein interactors. e) Individual compound spots are excised and analyzed via LC‐MS after digestion, f) enabling label‐free quantification and identification of specific protein‐small molecule interactions. Redrawn and adapted from Melder et al.,^[^ ^135^ ^]^ licensed under CC BY. © 2022 Wiley. Created with BioRender.com.

Melder et al.^[^ ^135^ ^]^ used a method in which small molecules were immobilized on cellulose membranes to study the interactions of multiple proteins with multiple small molecules simultaneously. This process involved functionalizing the cellulose membrane with diazirine **40** via EDC coupling to immobilize the small molecules with undirected photo crosslinking. After incubation with protein extracts and washing, the individual spots were isolated and analyzed with mass spectrometry (Figure 42). The specific interaction partners of each compound were identified through label‐free quantification.

High‐throughput screening has become an indispensable tool for exploring chemical reactivity and optimizing catalytic systems, yet many analytical formats still depend on labeling strategies or chromatographic separation, which can slow analysis and bias results. The self‐assembled monolayers for matrix‐assisted laser desorption/ionization (SAMDI) approach combines surface chemistry with mass spectrometry to achieve rapid, quantitative assessment of reactions without conventional labeling. In SAMDI, analytes are immobilized on alkanethiol monolayers supported on gold surfaces and directly analyzed by MALDI‐MS, providing a powerful means to follow enzymatic and chemical transformations.

**Figure 43:**
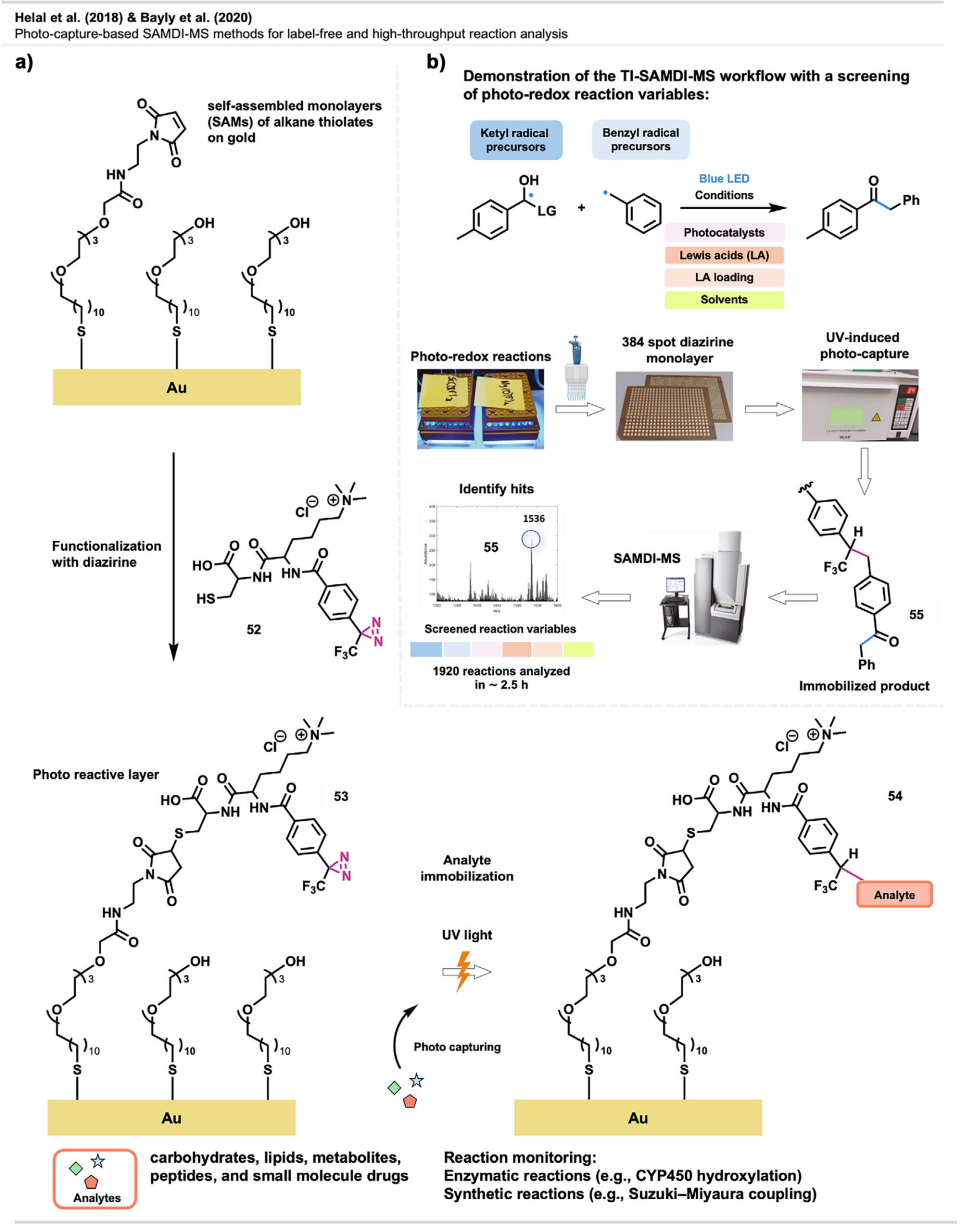
Traceless Immobilization SAMDI‐MS a) Diazirine‐modified SAMs on gold enable UV‐triggered carbene insertion for tag‐free photocapture and SAMDI‐MS quantification of reaction products. b) Integration into a 384‐spot diazirine‐coated platform enabled automated high‐throughput reaction screening with rapid SAMDI‐MS readout for efficient reaction discovery. Section b is in parts a direct reproduction from Bayly et al.^[^ ^137^ ^]^ © 2022 PNAS under the PNAS license.

The Traceless Immobilization SAMDI‐MS (TI‐SAMDI‐MS) platform^[^ ^136^ ^]^ extends the capabilities of surface‐assisted mass spectrometry to a fully label‐free, high‐throughput analytical method (Figure 43a). In this approach, diazirine **52** was used to functionalize SAMs on gold. Upon UV irradiation, the carbene derived from **53** inserts into C─H, N─H, O─H, and other bonds, enabling covalent immobilization of a broad range of small molecules without chemical modification (**54**). Using this photocapture strategy, the authors immobilized and analyzed a diverse set of compounds, including glucose, caprylic acid, lactic acid, the tripeptide Glu‐Val‐Phe, and the drug warfarin, demonstrating compatibility with molecules of different polarity and functionality. The method was validated with both biochemical and synthetic reactions. In enzymatic assays, CYP2C9‐catalyzed hydroxylation was followed over time, allowing extraction of kinetic parameters in agreement with HPLC benchmarks. In a complementary demonstration, TI‐SAMDI‐MS was applied to a Suzuki–Miyaura cross‐coupling reaction.

Bayly et al.^[^ ^137^ ^]^ expanded the TI‐SAMDI‐MS concept into a high‐throughput photocapture platform for rapid reaction discovery (Figure 43b). Using a 384‐spot diazirine‐terminated SAM, they analyzed about 2000 photoredox reactions within 2.5 h, which is orders of magnitude faster than LC/MS or GC/MS. This method enabled the investigation of single‐electron reductive coupling between ketyl radicals, generated from acyl azolium precursors, and benzyl radicals formed from benzyl radical precursors, yielding a benzyl‐substituted alpha‐hydroxy ketone **55**. The approach delivers quantitative and reproducible data suitable for kinetic analysis, catalyst screening, and reaction discovery, with strong potential for use in chemical biology, drug metabolism studies, and automated high‐throughput experimentation where rapid and unbiased analysis is essential. Key advantages include high speed, reproducibility that correlates with bulk yields, and broad applicability across diverse reaction classes, ranging from enzymatic to transition‐metal‐catalyzed transformations. Current limitations include a detection threshold of roughly 30 to 40 percent product formation and possible signal interference in highly complex mixtures. Overall, TI‐SAMDI‐MS emerges as a practical, scalable, and general tag‐free analytical platform that enables fast and chemically versatile reaction profiling, opening new opportunities for reaction discovery, mechanistic investigation, and catalyst evaluation.

### 4.5 Diazirines as Crosslinkers in the Assembly of Bioadhesives

Bioadhesives are polymers that can adhere to biological surfaces such as skin, mucous membranes, and internal tissues. These types of adhesives are typically made from natural or biocompatible materials and are designed to be non‐toxic and biodegradable.^[^ ^138^, ^139^ ^]^

Bioadhesives are used in various medical and industrial applications, including wound healing and tissue repair,^[^ ^140^, ^141^ ^]^ drug delivery,^[^ ^142^, ^143^, ^144^ ^]^ and dental applications.^[^ ^145^, ^146^ ^]^ They offer a less invasive alternative to traditional wound closure methods such as sutures, wires, and staples, and have the potential to enhance wound healing while minimizing tissue damage. These adhesives can also possess antimicrobial,^[^ ^147^ ^]^ anti‐inflammatory,^[^ ^148^ ^]^ and antioxidant^[^ ^149^ ^]^ properties that aid in the healing process. They can also be used to create more environmentally friendly products, such as biodegradable packaging materials.^[^ ^150^ ^]^

There are several different types of bioadhesives, including protein‐based bioadhesives,^[^ ^151^ ^]^ which are derived from structural proteins such as collagen or fibrin, polysaccharide‐based bioadhesives^[^ ^152^, ^153^ ^]^ made from cellulose or dextran, as well as polymer‐based ones. These adhesives are made from synthetic polymers such as polyethylene glycol and cyanoacrylates.^[^ ^154^ ^]^ The latter, commonly known as superglues, have been widely used in households and industries. Their properties, such as high reactivity, fast polymerization at room temperature, and strong adhesion to tissue through covalent bonding with protein functional groups, also make them attractive for medical applications. However, the breakdown of some polymers can result in the accumulation of degradation products in tissue. For instance, the breakdown of cyanoacrylate glues occurs through hydrolysis, releasing cytotoxic components such as cyanoacetate and formaldehyde, which can lead to inflammatory responses.^[^ ^155^ ^]^ Additionally, cyanoacrylates have poor mechanical properties, becoming brittle after polymerization and prone to fracture when used in long incisions. Some cases of foreign body reactions have also been reported after the use of cyanoacrylates.^[^ ^156^ ^]^ In addition, many bioadhesives require photoinitiators for polymerization that might produce cytotoxic free radicals.

Diazirine crosslinking is a promising strategy for overcoming the aforementioned limitations in material properties, as it allows for tuning of mechanical properties and omits the use of potentially toxic photoinitiators and their products. They are easily activated, produce only innocent nitrogen as a by‐product (in contrast to, e.g., cyanocatechol or formaldehyde), and can readily adhere to tissues, improving the mechanical properties of biocompatible polymers that have previously shown poor adhesion.

The following chapter discusses various curing methods—including UV and electrical activation—and explores diverse materials and applications of diazirines in bioadhesives.

**Figure 44:**
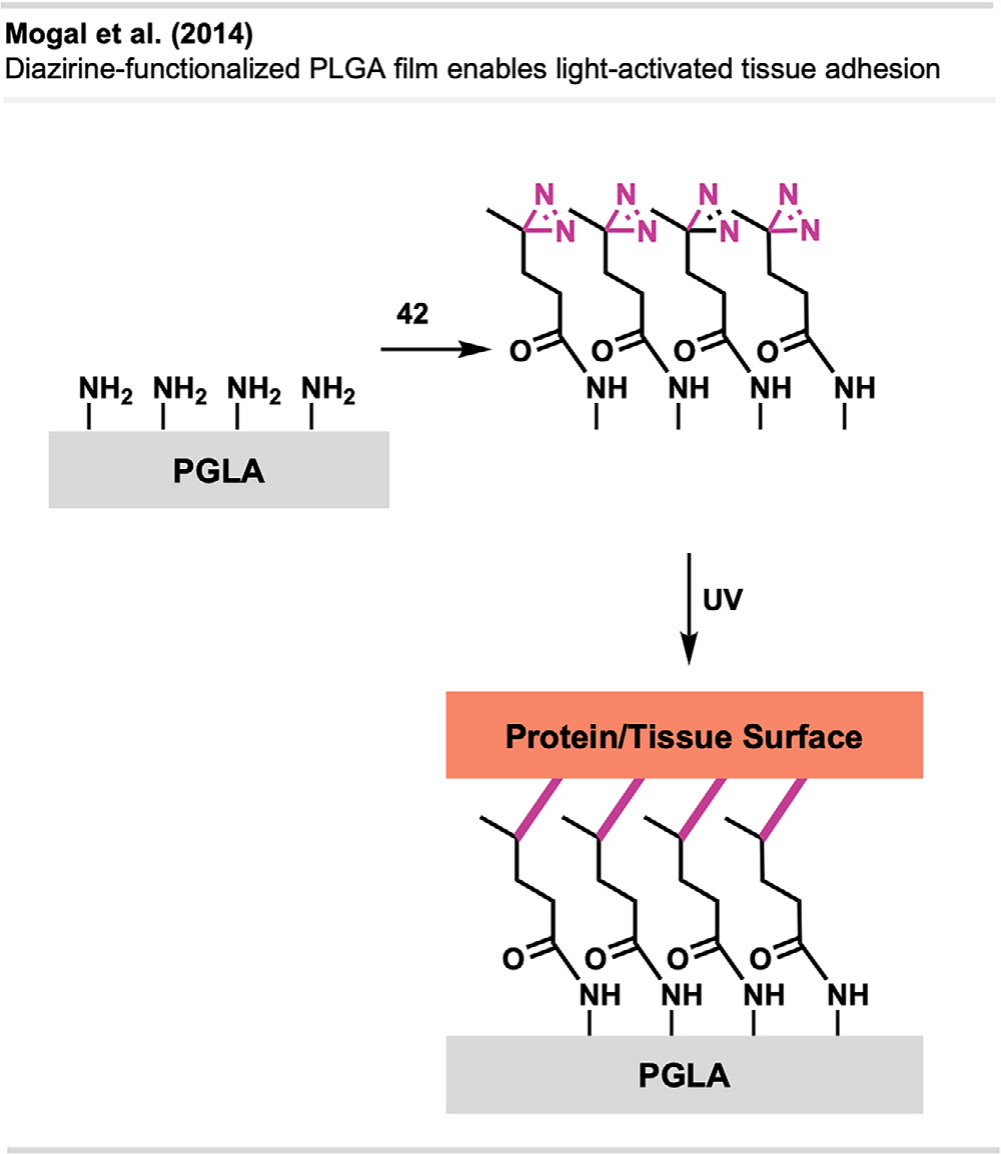
Crosslinking of a PGLA film to proteins or a tissue surface.

In this field, the Steele research group has made notable contributions. In 2014, for instance, Mogal et al.^[^ ^157^ ^]^ introduced a novel diazirine‐based bioadhesive designed for precise and on‐demand tissue adhesion in wet environments. A poly(lactic‐co‐glycolic acid) (PLGA) film modified with amine groups was immobilized with NHS‐diazirine **42** (Figure 44). Upon UV exposure, the carbenes enabled strong covalent bonding to tissue substrates. The resulting light‐activated system demonstrated high shear strength and cohesive failure in wet environments, outperforming conventional adhesives. With this work, the authors first demonstrated that diazirine‐functionalized surfaces can serve as effective light‐triggered adhesives for biomedical use.

In recent years, numerous publications have emerged showcasing bioadhesives based on PAMAM dendrimers. These dendrimers are hyperbranched polymers that are built up in an iterative fashion, consisting of an ethylenediamine core, to which an acrylic ester is added via a Michael‐type addition, followed by an amidation reaction with ethylenediamine. Starting from the ethylenediamine core, a branched network of amidoamine subunits is created, terminally bearing free amines. These can either serve as the starting point of additional amidoamine‐subunit introduction (colloquially termed a new “generation”) or be used as a handle for introducing diverse functionalities. This iterative manufacturing process allows the formation of nanosized particles with discrete size, molecular weight, and shape.^[^ ^158^, ^159^ ^]^ In the context of the presented application, the unfunctionalized NH_2_‐groups on the dendrimer's surface also served as the points of carbene‐insertion, effectively facilitating the curing process.

**Figure 45:**
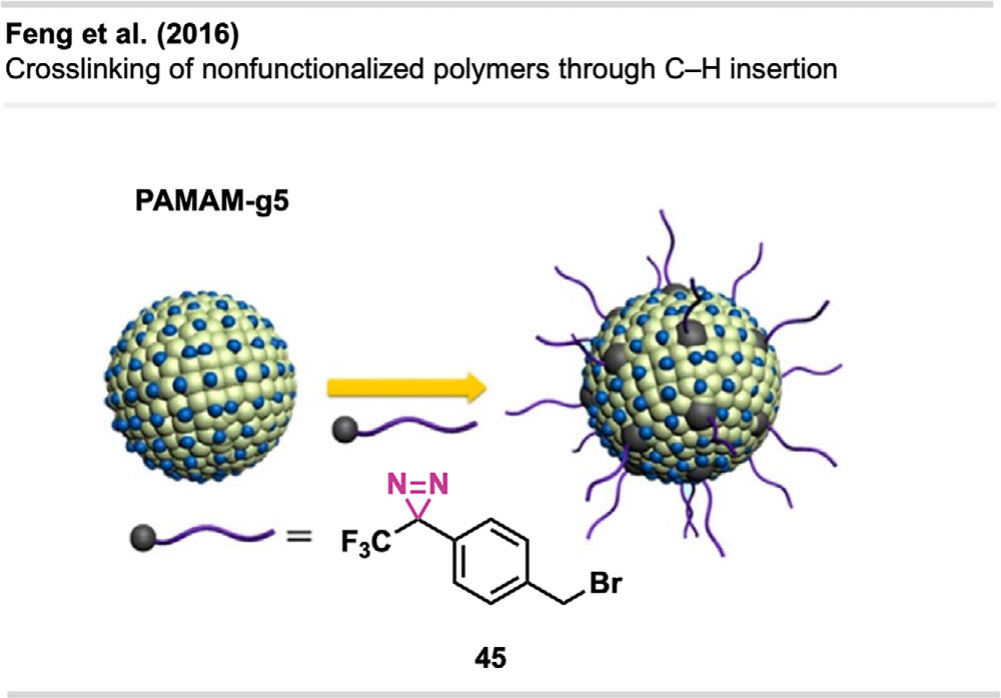
Formation of PAMAM‐Dz. Diazirine **45** is linked to the dendrimer via nucleophilic substitution, leaving terminal diazirine functionalities. Redrawn and adapted from Ping et al.,^[^ ^160^ ^]^ licensed under CC BY. © 2015 Springer Nature. Created with BioRender.com.

Feng et al.^[^ ^158^ ^]^ developed a UV‐activated bioadhesive based on PAMAM dendrimers grafted with diazirine **45** (Figure 45), addressing limitations of conventional adhesives such as weak bonding, toxicity, and poor adaptability. The diazirine‐modified PAMAM forms covalent bonds with tissue upon low‐energy UV exposure, enabling rapid, on‐demand crosslinking without toxic initiators. The mechanical and adhesive properties of the resulting hydrogels were tunable by controlling diazirine conjugation, dendrimer concentration, and UV dose. Ex vivo studies on swine arteries showed strong adhesion and excellent tissue conformity. Cytotoxicity tests revealed that diazirine modification reduced PAMAM's inherent toxicity, making it more biocompatible.

Shah et al.^[^ ^161^ ^]^ developed a non‐aqueous photoactivatable bioadhesive system based on diazirine‐grafted PAMAM dendrimers (PAMAM‐Dz) and PEG to address key limitations in soft tissue fixation. Their work focused on reducing viscosity and improving wet adhesion by blending PAMAM‐Dz with PEG 400.^[^ ^162^ ^]^ This enabled syringe delivery and rapid UV‐curing (≤45 s) into viscoelastic networks with tunable mechanical properties, high adhesion strength, and significantly reduced platelet activation, attributed to PEG's shielding effect.

**Figure 46:**
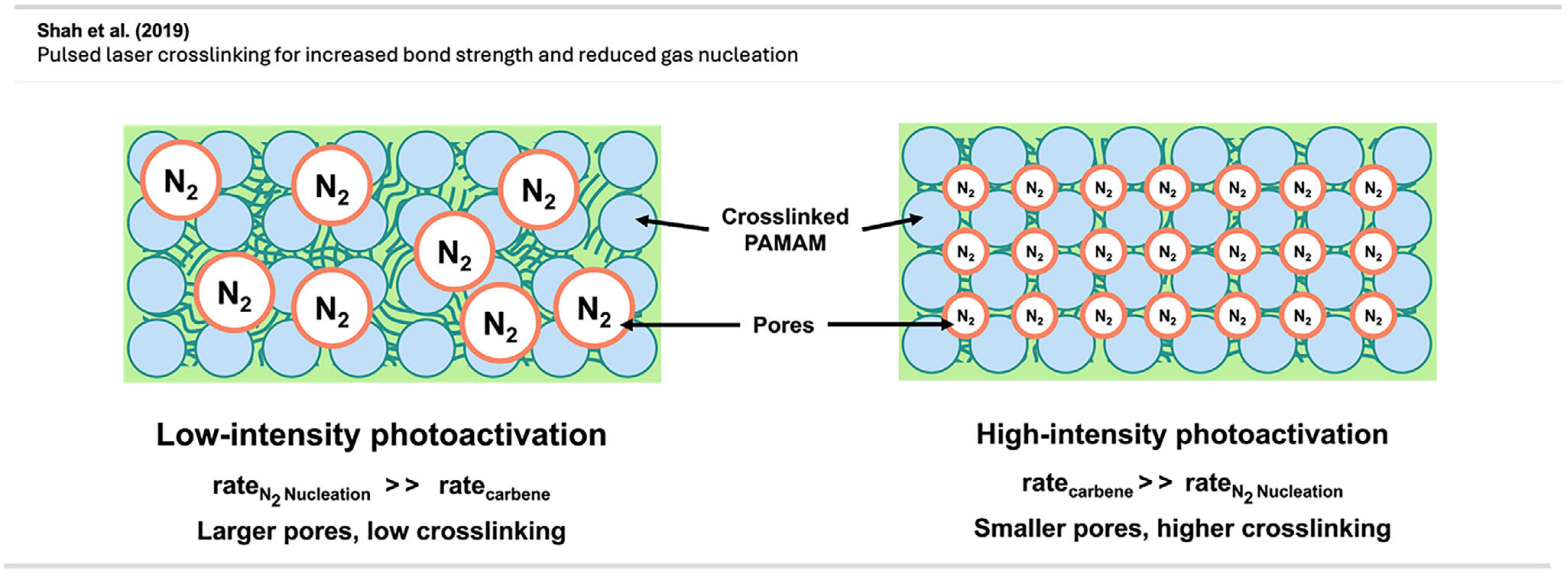
Schematic comparison of diazirine‐based adhesive crosslinking under continuous versus pulsed UVA irradiation. Under continuous irradiation (left), slow carbene formation allows nitrogen (N_2_) gas nucleation to dominate, resulting in large gas bubbles and pore formation that reduce crosslinking density and compromise mechanical integrity. In contrast, pulsed UVA activation (right) accelerates carbene generation, outpacing N_2_ nucleation and minimizing pore formation, leading to a denser, more uniform adhesive network. Redrawn and adapted from Shah et al.,^[^ ^162^ ^]^ permission acquired via RightsLink. © 2019 Wiley. Created with BioRender.com.

Another study introduced pulsed UVA laser irradiation to overcome crosslinking inefficiencies caused by gas nucleation and poor light penetration.^[^ ^162^ ^]^ The nitrogen (N_2_) gas released as a byproduct of carbene formation can form gas bubbles within the adhesive matrix. These bubbles create microscopic pores that act as scattering centers, disrupting uniform light transmission and thereby hindering efficient crosslinking. As shown in Figure 46, the application of pulsed UVA laser irradiation enhances the rate of carbene generation while limiting the accumulation of gas bubbles, resulting in a more uniform and tightly crosslinked network with minimal structural imperfections. The optimized PAMAM‐Dz 5050 formulation outperformed fibrin glue and commercial cyanoacrylate‐based Dermabond, highlighting the platform's potential for minimally invasive and vascular applications.

In a subsequent study,^[^ ^163^ ^]^ a tertiary blend of PAMAM‐Dz, PEG400, and high molecular weight PEGs (2–10 kDa) was formulated to fine‐tune mechanical properties. This solvent‐free adhesive crosslinks rapidly under mild UVA exposure to yield elastic films with enhanced wet adhesion and injectability, offering a modular and scalable strategy for soft tissue repair and drug delivery applications.

Further examples where diazirines and PAMAM dendrimers were employed include solvent‐free, UVA‐curable adhesives by Wicaksono et al.,^[^ ^164^ ^]^ using PAMAM‐Dz and PCL (polycaprolactone) triols/tetrols to form elastic networks with strong wet adhesion and minimal swelling. Singh et al. developed two PAMAM‐Dz‐bacterial cellulose systems: a UV‐curable dry‐film patch and an injectable hydrogel, both enabling rapid crosslinking in moist environments such as the oral cavity.^[^ ^165^, ^166^ ^]^

**Figure 47:**
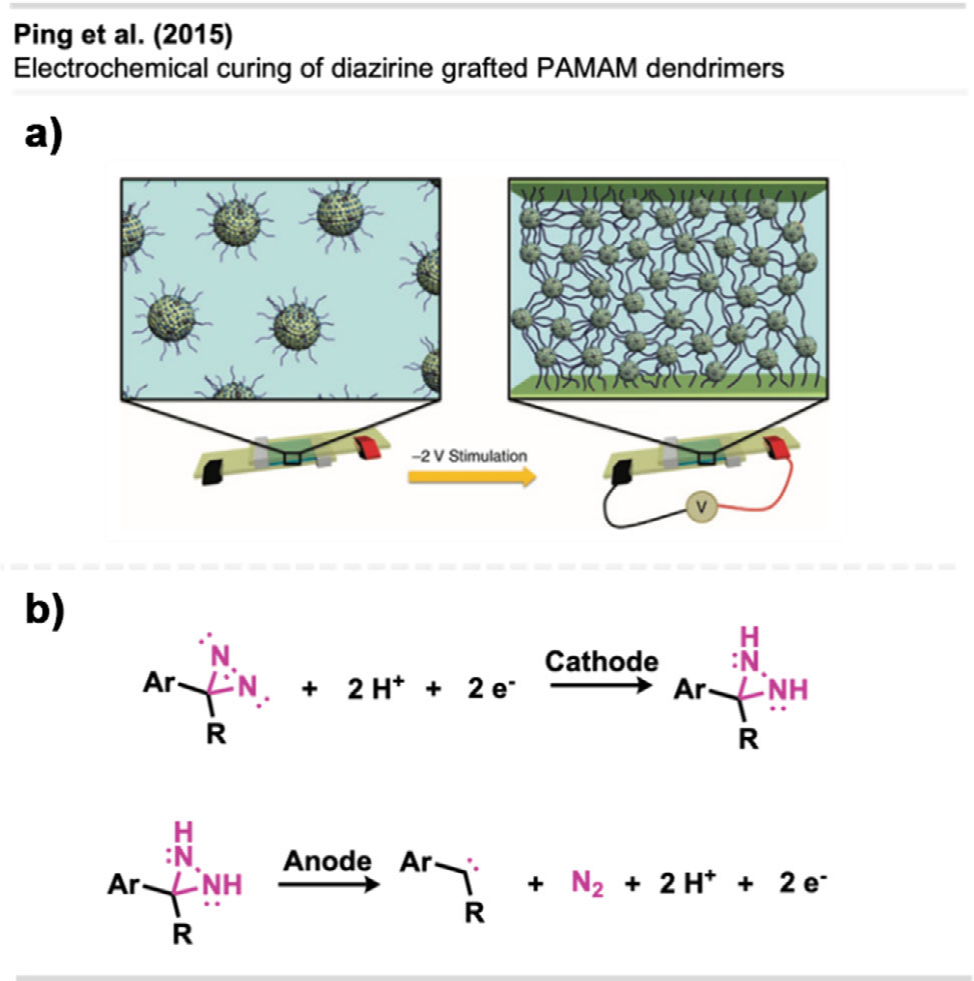
a) Schematic depiction of electrical curing of diazirine‐grafted PAMAM dendrimers. b) Proposed redox processes at the electrodes. At the cathode, aryl‐diazirine undergoes reduction to form aryl‐diaziridine, consuming two protons and two electrons. This intermediate is subsequently oxidized at the anode to generate the aryl‐carbene, releasing two electrons, two protons, and nitrogen gas. Section a is a direct adaptation from Ping et al.,^[^ ^160^ ^]^ licensed under CC BY. © 2015 Springer Nature.

Ping et al.^[^ ^160^ ^]^ reported the first example of an electrochemically curable, diazirine‐based bioadhesive, employing aryl diazirine grafted PAMAM dendrimers (Figure 47a). As noted by the authors of this seminal publication, electrochemical curing offers several advantages over classical activation methods, such as anionic polymerization and thermo‐ and photochemical approaches. These advantages include its applicability to heat‐sensitive and non‐transparent surfaces, as well as the absence of potentially harmful or sensitizing initiators. Unlike electropolymerization,^[^ ^167^, ^168^ ^]^ which typically confines polymer growth to the electrode surface, electrocuring enables bulk crosslinking throughout the entire material. Additionally, it was anticipated that curing would start only when a specific voltage threshold was reached and cease immediately once the voltage dropped below this threshold. This characteristic allows for precise temporal control of the crosslinking process, enabling fine‐tuning of material properties in relation to crosslink density.

The anticipated “on–off” nature is further reflected in the proposed activation mechanism (Figure 47b). In simplified terms, the aryl diazirine is reduced to the diaziridine at the cathode, followed by the anodic oxidation to nitrogen and the aryl carbene, which is responsible for crosslinking via insertion into free amino‐groups on the surface of other PAMAM dendrimers.

**Figure 48:**
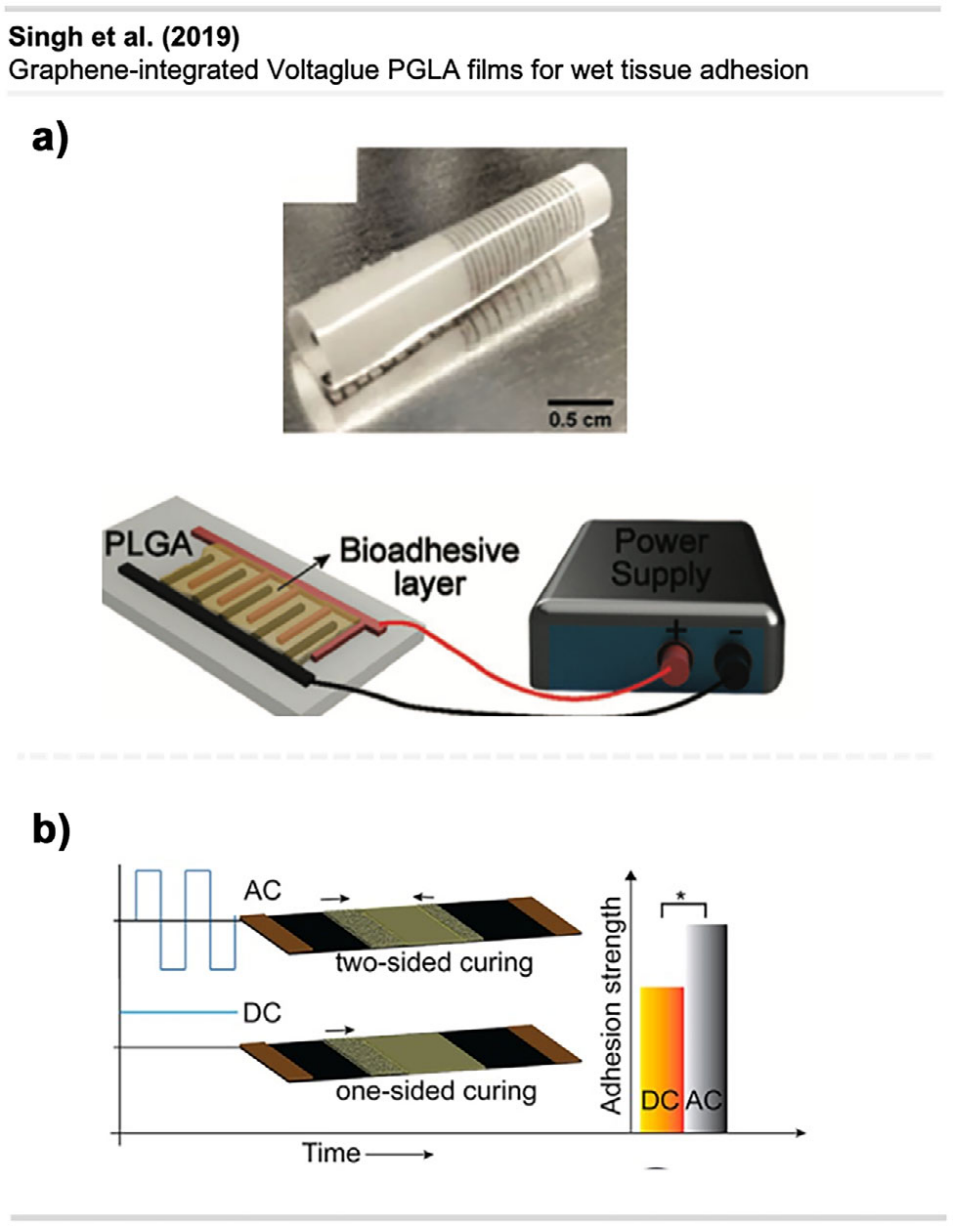
a) Voltaglue platform with 3D‐printed graphene electrodes on biodegradable PLGA films. b) Two‐sided curing using AC stimulation improves adhesion strength. Section a is a direct reproduction from Singh et al.,^[^ ^170^ ^]^ permission acquired via RightsLink. © 2019 Wiley. Section b is a direct reproduction from Singh et al.,^[^ ^25^ ^]^ permission acquired via RightsLink. © 2020 American Chemical Society.

Singh et al.^[^ ^169^ ^]^ developed a voltage‐activated bioadhesive platform—Voltaglue—by combining diazirine‐grafted PAMAM dendrimers with interdigitated 3D‐printed graphene electrodes on biodegradable PLGA films (Figure 48a). Upon electrical stimulation (5–10 V), the adhesive rapidly crosslinked within 60 s, forming viscoelastic hydrogels with tunable mechanical properties and strong adhesion to moist tissues. Adhesion strength correlated with voltage and electrolyte concentration. Compared to traditional fixation methods and UV‐based adhesives, this system offers on‐demand, localized curing without thermal injury or optical limitations. Cytotoxicity and platelet adhesion studies confirmed improved biocompatibility at higher voltages due to reduced leachates and surface activation. In follow‐up studies,^[^ ^25^ ^]^ a flexible electroceutical patch using direct current (DC) to trigger spatially controlled, carbene‐mediated tissue adhesion with tunable strength (Figure 48b) was introduced.

The concept was further advanced by applying alternating current (AC) stimulation, enabling two‐sided curing, faster gelation, and enhanced mechanical performance.^[^ ^25^ ^]^ These studies collectively establish Voltaglue as a modular, low‐voltage electrocuring adhesive with promising applications in wound healing, bioelectronics, and minimally invasive medical devices.

**Figure 49:**
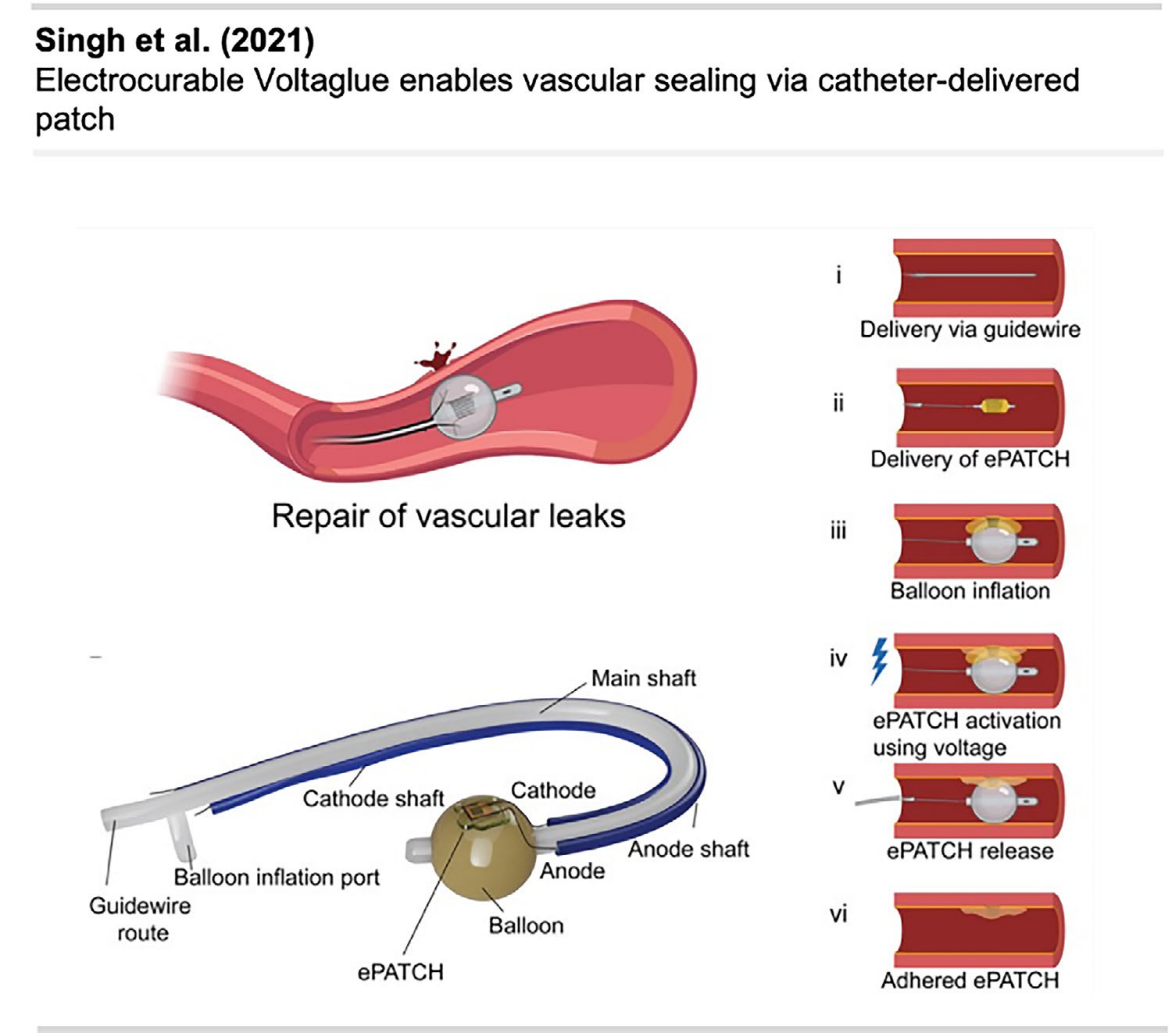
Schematic overview of the electroceutical catheter system for vascular leak repair, illustrating the procedural steps and the device design. The setup enables localized activation and sealing of vascular defects through electrically triggered adhesive crosslinking. The figure is a direct reproduction from Singh et al.,^[^ ^171^ ^]^ licensed under CC BY. © 2021 The Authors of the original publication.

An example highlighting the versatility of diazirine‐mediated electrocuring was demonstrated using a minimally invasive electroceutical catheter system for sealing vascular leaks.^[^ ^171^ ^]^ The system consisted of a balloon catheter (CATRE) and an electrode patch (ePATCH) coated with Voltaglue, which crosslinked upon electrical stimulation at the desired location (Figure 49). The catheter delivered effective sealing of 2–3 mm vascular defects in ex vivo and in vivo models. Adhesion tests showed superior shear strength and burst pressure compared to commercial sealants. Crucially, Voltaglue maintained biocompatibility and did not interfere with physiological electrical signals.

**Figure 50:**
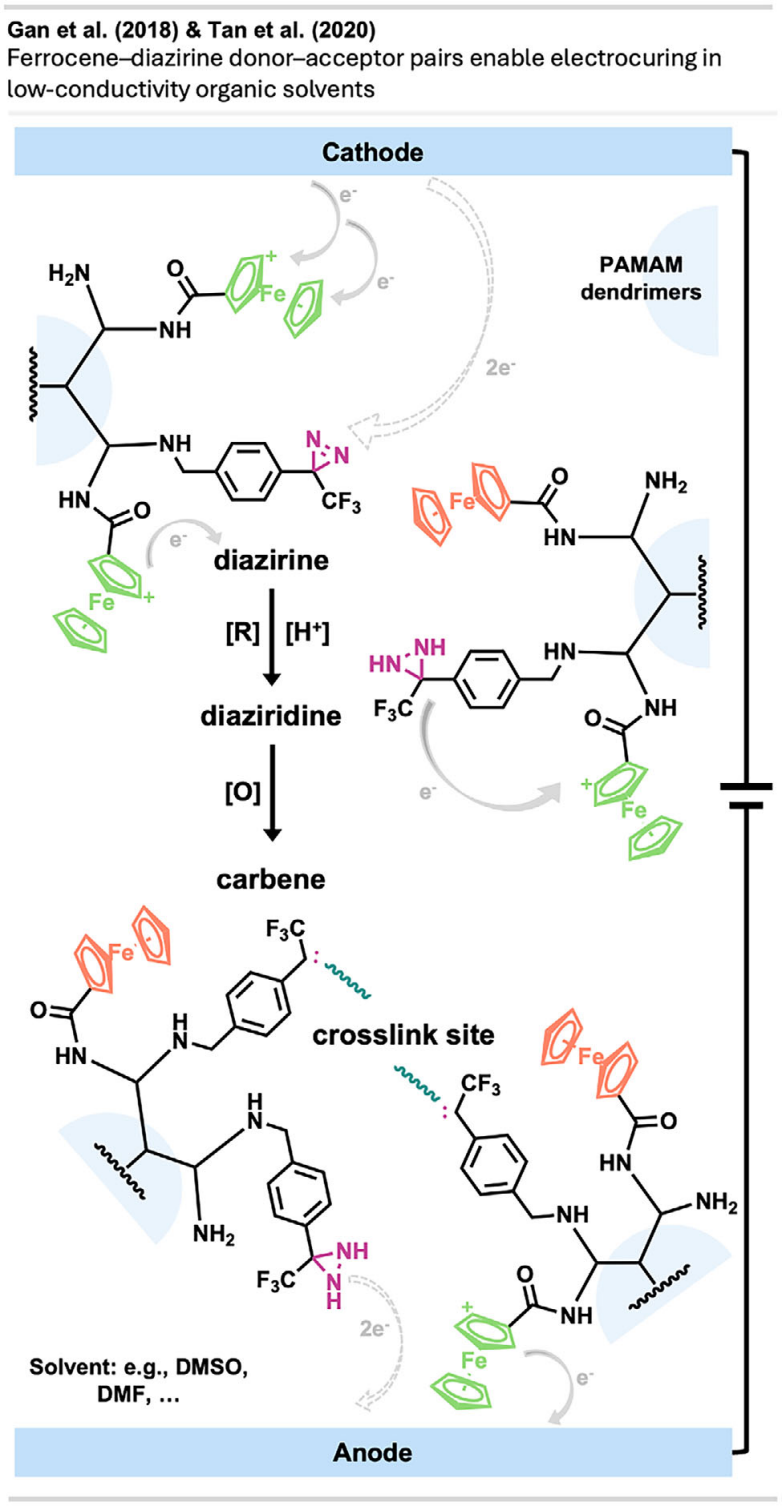
Simplified mechanism of redox‐triggered carbene formation and crosslinking in a PAMAM‐diazirine‐ferrocene donor–acceptor system in low‐conductivity organic solvents in the presence of 1.0% acetic acid, serving as a proton source. Redrawn and adapted from Gan et al.,^[^ ^172^ ^]^ permission acquired via RightsLink. © 2018 American Chemical Society.

Gan et al.^[^ ^172^ ^]^ and Tan et al.^[^ ^173^ ^]^ explored electrocurable adhesives based on PAMAM dendrimers, functionalized diazirines, and ferrocene to obtain donor–acceptor pairs. The donor–acceptor pair consisted of ferrocene as the electron donor and diazirine as the electron acceptor, enabling redox‐triggered carbene formation. This internal electron transfer facilitated crosslinking even in low‐conductivity organic solvents. With this strategy, the cografts could also be cured in organic solvents of low conductivity, such as DMSO, DMF, or PEG400. The supposed hole conduction and carbene activation mechanism is depicted in Figure 50.^[^ ^172^ ^]^

**Figure 51:**
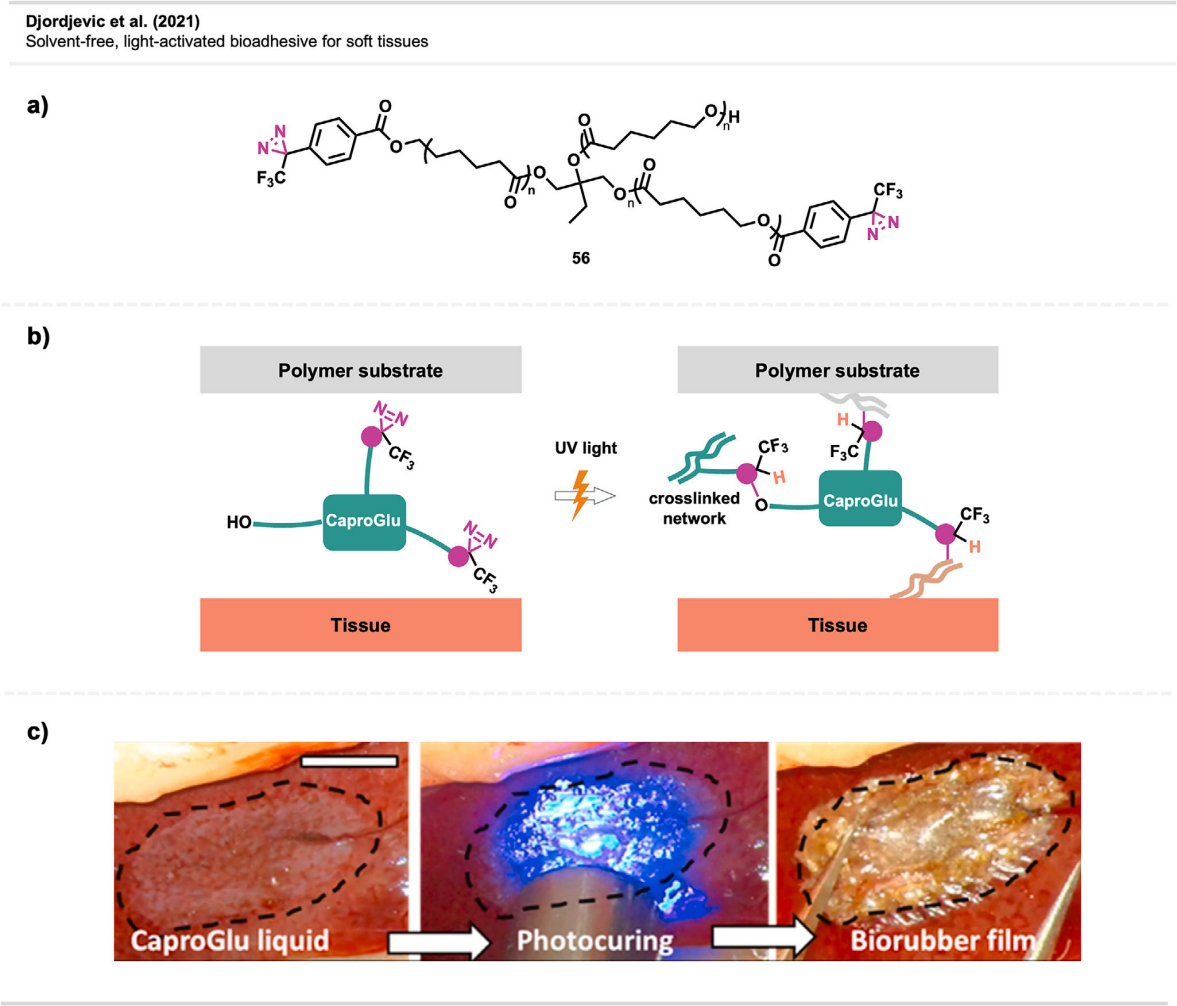
a) Chemical structure of CaproGlu. b) Schematic overview of CaproGlu adhesion to tissue. c) UVA‐triggered liquid‐to‐elastomeric transition of CaproGlu (a PCLT‐D70 and 10% w/w citric acid formulation) on a hydrated rabbit liver surface, followed by demonstration of tissue adhesion using a pull test with tweezers (scale bar = 5 mm). Section c is a direct reproduction from Djordjevic et al.,^[^ ^174^ ^]^ permission acquired via RightsLink. © 2021 Elsevier. Created with BioRender.com.

Inspired by the results obtained using polycaprolactone triols (PCLT) to tune the viscosity of PAMAM‐g‐diazirine, Djordjevic et al.^[^ ^174^ ^]^ developed a novel bioadhesive platform termed CaproGlu **56** (Figure 51a). This solvent‐free system was based on food‐grade PCLT modified with TPDs and exhibited excellent performance as a bioadhesive via carbene‐mediated crosslinking to tissue and self‐crosslinking to its terminal OH‐groups (Figure 51b). It can rapidly transition from a liquid with low viscosity to a bio rubber material upon photocuring, with excellent ability to adhere to wet surfaces and providing compliant tissue approximation (Figure 51c). The material properties could be easily adjusted by altering the ratio of grafted diazirine, the UVA dose, and the choice of additive (such as citric acid, hydroxyapatite, or sebacic acid).

The CaproGlu platform has great potential for tissue repair, wound healing, and delivering therapeutic agents. It has been assessed on preclinical models for specific medical needs, including strain‐activated analgesia, vascular anastomosis, and modulation of human platelet interactions. CaproGlu is easy to use, can be formed into implants without the need for harmful organic solvents, and can provide long‐lasting pain relief. The bio‐adhesive was also evaluated for its biocompatibility. Experiments showed no risk for genotoxicity or skin sensitization.^[^ ^175^ ^]^

**Figure 52:**
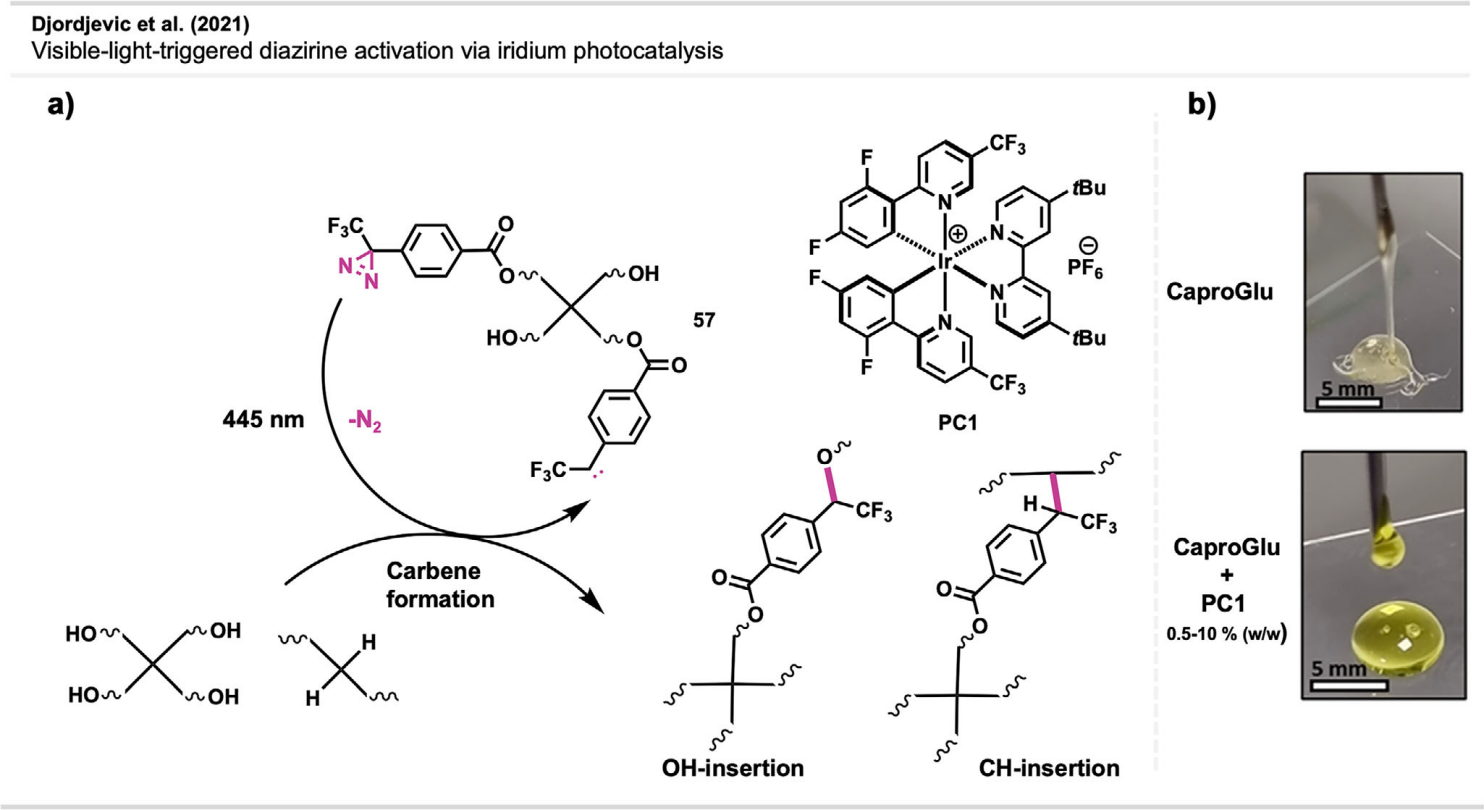
a) Schematic illustration of CaproGlu activation via visible light (445 nm) enabled by photocatalysis. a) Digital image of the solvent‐free, syringeable CaproGlu formulation (18‐gauge needle) alongside the chemical structure depicting diazirine‐to‐carbene conversion; b) Structure of the photocatalyst PC1 and image of CaproGlu undergoing a visible color change upon mixing with PC1 at concentrations between 0.5% and 10% (w/w). Section a was redrawn and adapted from Djordjevic et al.,^[^ ^176^ ^]^ while Section b is a direct reproduction from the same publication. Permission acquired via RightsLink. © 2021 American Chemical Society.

The UVA activation of CaproGlu still comes with potential limitations in terms of tissue exposure and light intensity. However, a continuative study by Djordjevic et al.^[^ ^176^ ^]^ presented a novel method of activating CaproGlu **57** using visible light‐emitting diodes (LEDs) at 445 nm (blue). To achieve that, iridium photocatalysts (**PC1**) were used to initiate carbene formation, even though diazirines do not absorb at 445 nm (Figure 52).

The activation mechanism is based on either triplet excitation energy transfer or photoredox catalysis with electron transfer to the diazirine. Both mechanisms are in line with the fact that no diazoalkane intermediate is formed. The direct conversion from diazirine to carbene resulted in enhanced crosslinking efficiency and accelerated gelation time compared to UVA activation. On top of that, the hydrogels formed showed superior adhesion strength to wet tissues compared to cyanoacrylates. The liquid organic matrix of CaproGlu dissolves the photocatalyst without the need for solvents and shows no cytotoxicity. The study is the first example of visible light activation of a diazirine‐grafted bioadhesive and enables the transition to longer wavelengths in medical applications.

The CaproGlu platform was further advanced by developing hybrid polymer networks that combine thiol/ene or thiol/yne crosslinking with diazirine‐grafted PCL to create injectable, light‐activated double‐sided adhesives.^[^ ^177^ ^]^ The system enabled dual‐step photoactivation: visible light (405 nm) triggers thiol/ene gelation, followed by UV light (365 nm) activating diazirine crosslinking. These hybrids offer tunable mechanics, strong wet adhesion, and robust bonding to tissues or synthetic surfaces, making them promising for biomedical applications.

**Figure 53:**
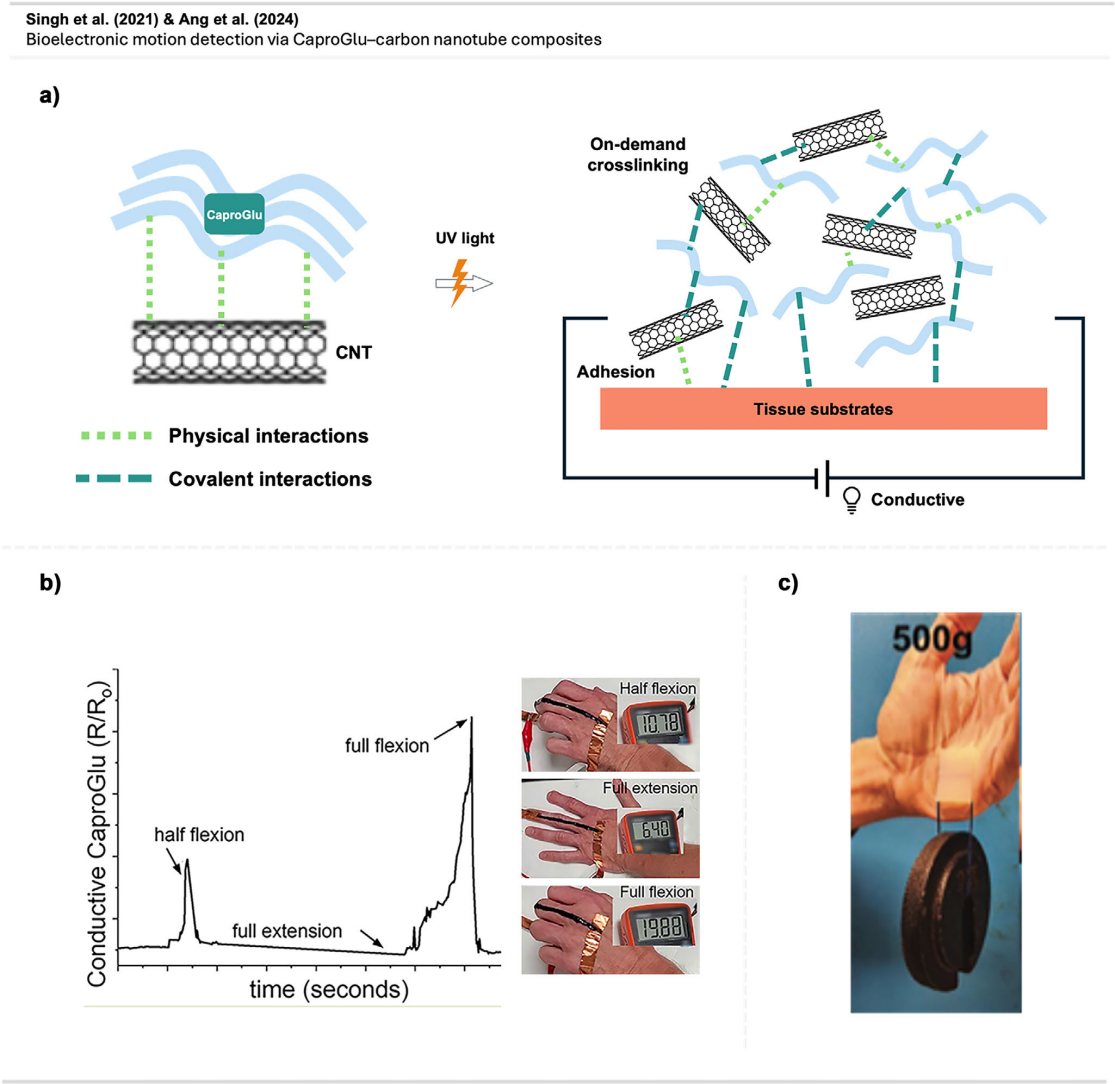
a) Schematic illustration of CaproGlu mixed with CNTs forming a conductive, tissue‐adhesive composite that undergoes covalent crosslinking upon UV (365 nm) exposure. b) Resistance measurements of the conductive CaproGlu‐CNT composite during finger motion (half flexion, full flexion, and full extension), demonstrating its sensitivity as a wearable strain sensor. c) Demonstration of strong adhesive strength of the cured CapruGlu through a dual stimulation strategy. Section a was redrawn and adapted from Singh et al.,^[^ ^178^ ^]^ while Section b is a direct reproduction from the same publication. Permission acquired via RightsLink. © 2021 American Chemical Society. Section c is a direct reproduction from Ang et al.,^[^ ^179^ ^]^ permission acquired via RightsLink. © 2024, Wiley.

Furthermore, a bioelectronic application for motion detection was developed by creating a composite material using CaproGlu and CNTs. The CNTs were crosslinked with CaproGlu to form a conducting network throughout the non‐conductive polymer matrix (Figure 53a). Additionally, CNTs strengthened the CaproGlu composite. The material was tested for its ability to sense strain and was successful in detecting human motion. The sensors were able to quickly to respond to bending motions and showed changes in resistance during movement (Figure 53b).^[^ ^178^ ^]^

In a recent study, Ang et al.^[^ ^179^ ^]^ focused on enhancing the mechanical performance of CaproGlu through a dual stimulation strategy combining UVA light and heat. By co‐applying thermal energy during photocuring, they significantly improved the cohesive strength and toughness of CaproGlu adhesives. At 90  °C, the adhesive showed a threefold increase in toughness. Adhesion tests on human skin (max. 35 °C) revealed strong (Figure 53c), uniform bonding with reduced UV dosage requirements, lowering phototoxic risk.

Eventually, a study was conducted to investigate the activation of PAMAM‐g‐Dz and CaproGlu using natural sunlight. The research aimed to determine the relationship between the adhesion strength of the materials and the amount of sunlight, the structure of the bioadhesive polymer, and the use of optical concentrators. The results showed that the bioadhesives are stable under indoor lighting but quickly activated under direct sunlight, making them potentially valuable for topical film‐forming polymers or sunscreen formulations.^[^ ^180^ ^]^

**Figure 54:**
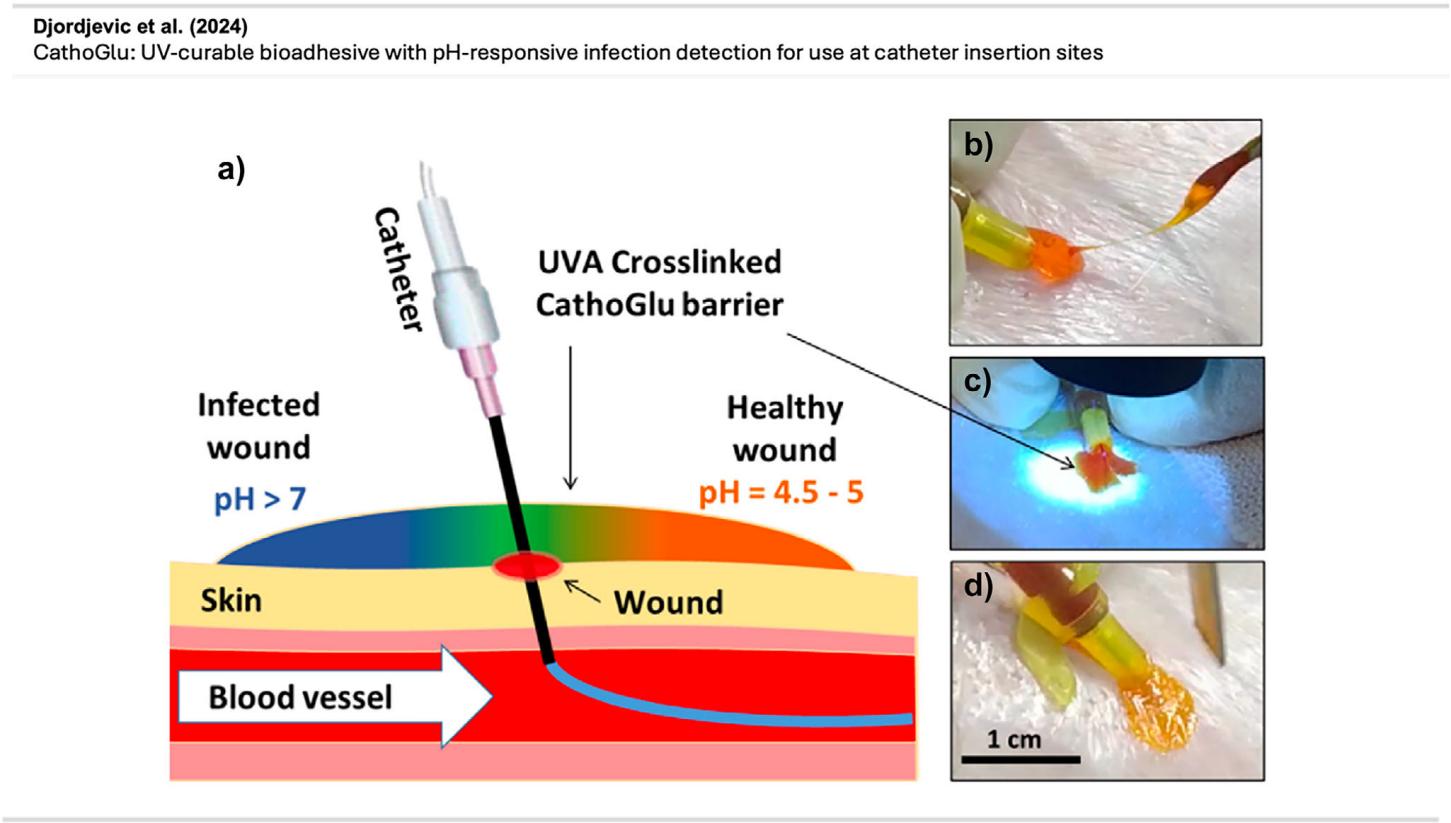
pH‐responsive sealing using CathoGlu. a) Application of CathoGlu via catheter, selectively forming a crosslinked barrier with bromothymol. Orange: acidic microenvironment of healthy tissue (pH 4.5–5) and blue in infected regions (pH > 7). b)–d) Sequential photographs showing the application of Cathoglue at the insertion site, followed by UVA activation, and formation of a stable adhesive barrier on the wound site. The figure is a direct reproduction from Djordjevic et al.,^[^ ^181^ ^]^ permission acquired via CCC Marketplace. ©2024, Royal Society of Chemistry.

A recent contribution by Djordjevic et al.^[^ ^181^ ^]^ represents a further advancement of caprolactone‐based bioadhesives, introducing CathoGlu, a diazirine‐functionalized PCL system engineered as a dual‐function barrier for catheter insertion sites (Figure 54). The adhesive leverages carbene crosslinking via photoactivation of diazirines (365 nm UVA) to form elastic, biocompatible films with strong tissue adhesion and low cytotoxicity. Notably, the hydrophobic diazirine‐containing matrix supports the integration of bromothymol blue, enabling pH‐sensitive color change (from orange to blue) as a visual clue for early bacterial infection. Crosslinked films significantly reduced *Escherichia coli* migration while maintaining film integrity, and an in vivo porcine model demonstrated performance on par with commercially available fixation methods.

Chandel et al. presented the first synthesis of injectable tissue‐adhesive hydrogels composed of diazirine‐modified hyaluronan and dendritic polyethyleneimine (DPI).^[^ ^182^ ^]^ Hyaluronic acid (HA) is a biocompatible, biodegradable polysaccharide with high water‐binding capacity, widely used in biomedical applications such as tissue adhesion, engineering, and cell culture due to its favorable physicochemical characteristics, biocompatibility, biodegradability, and non‐toxicity.^[^ ^183^, ^184^ ^]^

**Figure 55:**
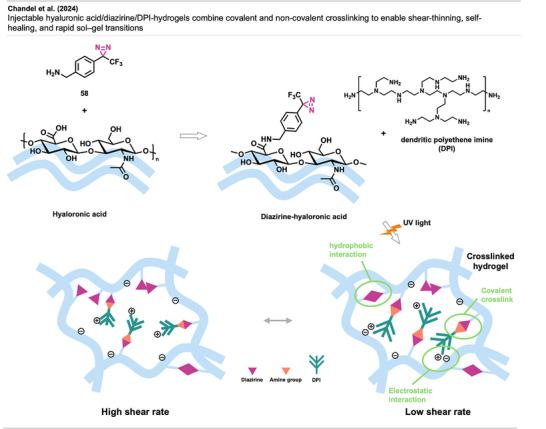
Shear‐thinning and photo‐crosslinkable hydrogel based on hyaluronic acid‐diazirine (HA‐Dz) and diamine polymer DPI. Chemical structures of HA‐Dz and diamine polymer DPI, with diazirine compound **58** as the crosslinking motif + illustration of shear‐thinning behavior: under low shear, strong electrostatic, hydrophobic, and hydrogen‐bond interactions maintain gel integrity, while high shear disrupts the network for injectability. The figure was redrawn and adapted from Chandel et al.,^[^ ^182^ ^]^ licensed under CC BY. ©2024 Royal Society of Chemistry. Created with BioRender.com.

The HA‐Dz solution, prepared through carbodiimide coupling using **58**, resulted in a hydrogel that showed excellent shear‐thinning and self‐healing properties, as well as excellent cytocompatibility (Figure 55). The HA‐Dz/DPI hydrogel formed through both covalent crosslinking and physical interactions such as hydrogen bonding, electrostatics, and hydrophobic forces. Before crosslinking, the precursor solution exhibited strong shear‐thinning behavior, where the viscosity decreases with increasing shear rate, due to electrostatic interactions between the amine‐rich DPI and the carboxyl groups of HA, as well as hydrophobic interactions from the diazirine moieties. Even after UV curing, the material retained shear‐thinning and self‐healing properties, suggesting that intermolecular (non‐covalent) interactions dominate over covalent bonds. Optimal DPI concentration was crucial, as excessive DPI reduced effective crosslinking between HA chains. The resulting compound exhibited outstanding adhesion performance and higher burst pressures than fibrin glues, making it particularly well‐suited for applications in tissue adhesion. The system remained stable under physiological conditions and degraded gradually over 2 weeks. The hydrogel's ability to flow under stress and recover when stress is removed enables controlled application through syringes or catheters while maintaining adhesion and structural integrity—ideal for tissue sealing and potentially for 3D bioprinting applications.

The researchers further developed a precursor polymer solution incorporating HA‐Dz and DPI, which underwent crosslinking via UV irradiation at 365 nm.

Yakufu et al.^[^ ^185^ ^]^ developed an ultra‐adhesive hydrogel paint designed for arthroscopic cartilage repair, leveraging carbene insertion chemistry using diazirine‐grafted gelatin together with HA to form a double‐network structure.

**Figure 56:**
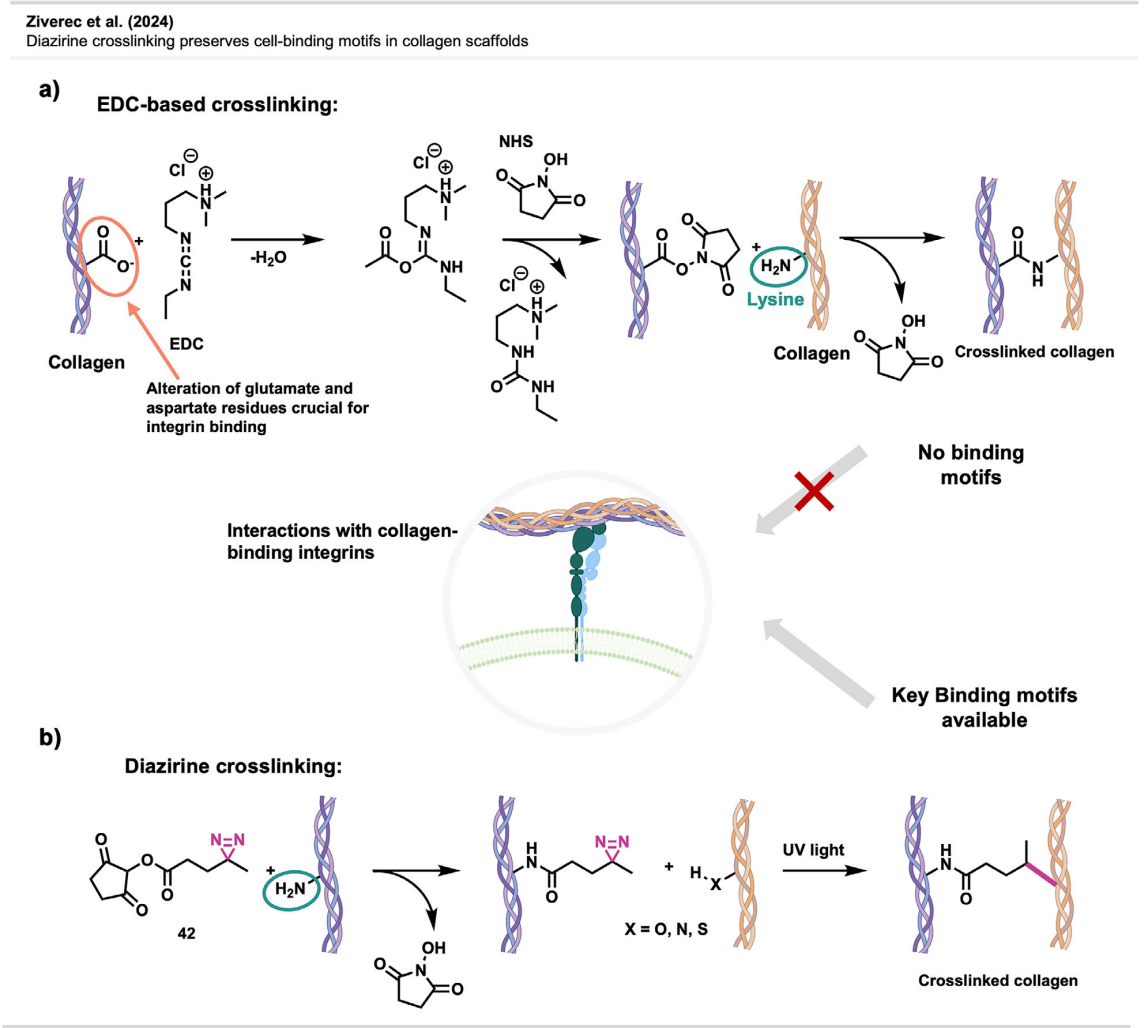
Comparison of collagen crosslinking strategies. a) Conventional EDC/NHS‐mediated crosslinking modifies carboxylic acid residues (e.g., glutamate and aspartate), which disrupts GxxGER motifs critical for integrin‐mediated cell attachment. b) Diazirine‐based photo‐crosslinking using NHS‐diazirine **42** selectively targets lysine residues via UV‐activated carbene insertion, preserving GxxGER motifs and enabling interaction with collagen‐binding integrins. The figure was redrawn and adapted from Ziverec et al.,^[^ ^186^ ^]^ permission acquired via RightsLink. © 2024 Elsevier. Created with BioRender.com.

Ziverec et al.^[^ ^186^ ^]^ introduced a diazirine‐mediated photo‐crosslinking strategy to improve the mechanical performance and biological compatibility of collagen scaffolds for tissue engineering. Unlike conventional EDC crosslinking, which alters glutamate/aspartate residues critical for cell attachment (Figure 56a), this approach selectively modifies lysine residues via UV‐triggered carbene insertion, preserving key integrin‐binding motifs. The diazirine‐treated scaffolds, derived from NHS‐diazirine **42,** demonstrated comparable stiffness to EDC‐crosslinked ones but retained greater cell affinity and integrin‐mediated interactions (Figure 56b). Mesenchymal stem cells (MSCs) exhibited strong attachment, spreading, and homogeneous colonization in diazirine‐crosslinked scaffolds, while EDC‐treated samples showed limited cellular infiltration. Mechanical and enzymatic assays confirmed improved stability, and mass spectrometry indicated minimal disruption to key residues. Notably, this method avoids cytotoxic byproducts and preserves biofunctionality, making it a tunable alternative for regenerative medicine and soft tissue repair.

## 5 Diazirines in NMR

Substantially different from their traditional application as carbene precursors is the utilization of diazirines in magnetic resonance derived techniques. Among those, MRI, has emerged as an effective tool in biomedical research and clinics as a noninvasive imaging technique to produce structural images of the anatomy and the body's physiological processes.^[^ ^187^ ^]^ However, one of the most critical drawbacks MRI faces is a low sensitivity arising from nuclear spin´s intrinsically low magnetic energy.^[^ ^188^ ^]^ This is particularly true for nuclei of low natural abundance, such as ^13^C and ^15^N. To address this barrier, hyperpolarization (HP) techniques^[^ ^189^ ^]^ have been developed to boost nuclear MR sensitivity by several orders of magnitude. Hyperpolarized low‐γ nuclei (^13^C, ^15^N) allow signal detection over prolonged periods due to their long relaxation times, enabling real‐time in vivo imaging of dynamic metabolic and physiologic processes.

Among different HP techniques, signal amplification by reversible exchange (SABRE) is a remarkably cost‐efficient and fast technique that utilizes a hyperpolarizable substrate and parahydrogen as an HP source on a catalyst. In this approach, *para*‐H_2_ and substrate transiently bind to an iridium catalyst, and the HP is transferred from *para*‐H_2_ to the substrate through *J*‐couplings established on the catalytic intermediate.^[^ ^190^ ^]^ Until recently, SABRE was limited to the HP of protons. However, Warren et al. extended the method to the mentioned SABRE‐SHEATH approach, enabling the direct HP of ^15^N molecular sites.^[^ ^191^, ^192^ ^]^

In this context, ^15^N‐diazirines show unique potential for molecular imaging owing to their desirable biocompatibility, minimal steric demands, and ease of incorporation in a wide range of biologically relevant small molecules, metabolites, and biomolecules.^[^ ^5^ ^]^ Furthermore, the two ^15^N atoms within ^15^N‐diazirines usually possess close chemical shifts and strong couplings, which supports long‐lived singlet states.^[^ ^193^, ^194^ ^]^

**Figure 57:**
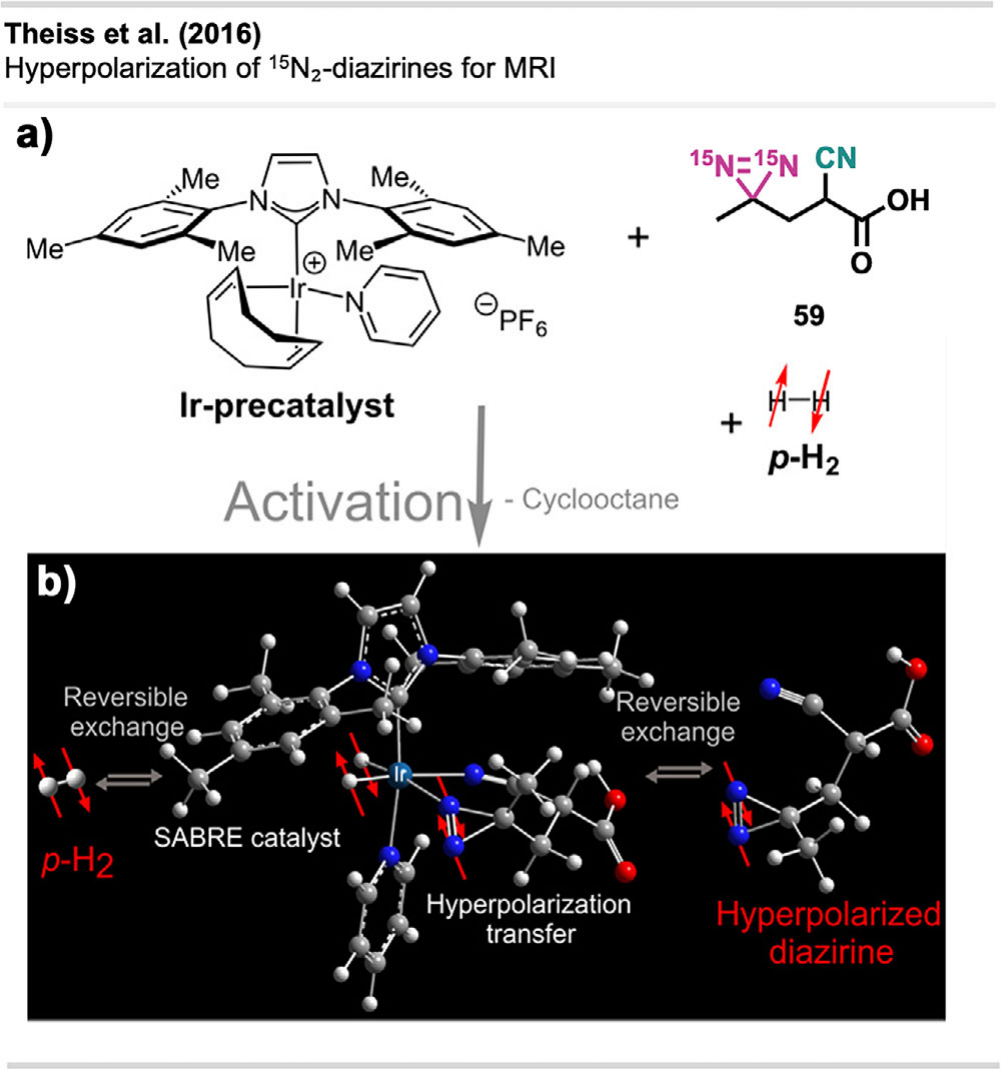
The HP mechanism of compound **59** and the Ir‐precatalyst. The figure is a direct reproduction from Theis et al.,^[^ ^195^ ^]^ licensed under CC BY.

Theis et al. were the first to recognize their potential for application in hyperpolarized MRI techniques.^[^ ^195^ ^]^ To explore the feasibility of ^15^N_2_‐diazirine as a potential imaging tag, they synthesized 2‐cyano‐3‐(D_3_‐methyl‐^15^N_2_‐diazirine)‐propanoic acid **59** along with the catalyst [Ir(COD)(IMes)(Py)][PF_6_] [COD, cyclooctadiene; IMes, 1,3‐bis(2,4,6‐trimethylphenyl)‐imidazolium; Py, pyridine] (Figure 57 **)**. Diazirine and the catalyst were dissolved in methanol and activated by bubbling hydrogen through the solution, resulting in the formation of the active [Ir(IMes)(H_2_)(Py)(Diaz)]^+^ species. Using this system, they demonstrated that ^15^N‐diazirine **59** could be hyperpolarized, achieving a 15 000‐fold signal enhancement (**ε**) over the thermal signal at the nitrogen atoms. Furthermore, the relaxation time constants of ^15^N_2_ magnetization and singlet spin order were found to be orders of magnitude larger than typical polarization decay time constants.

**Figure 58:**
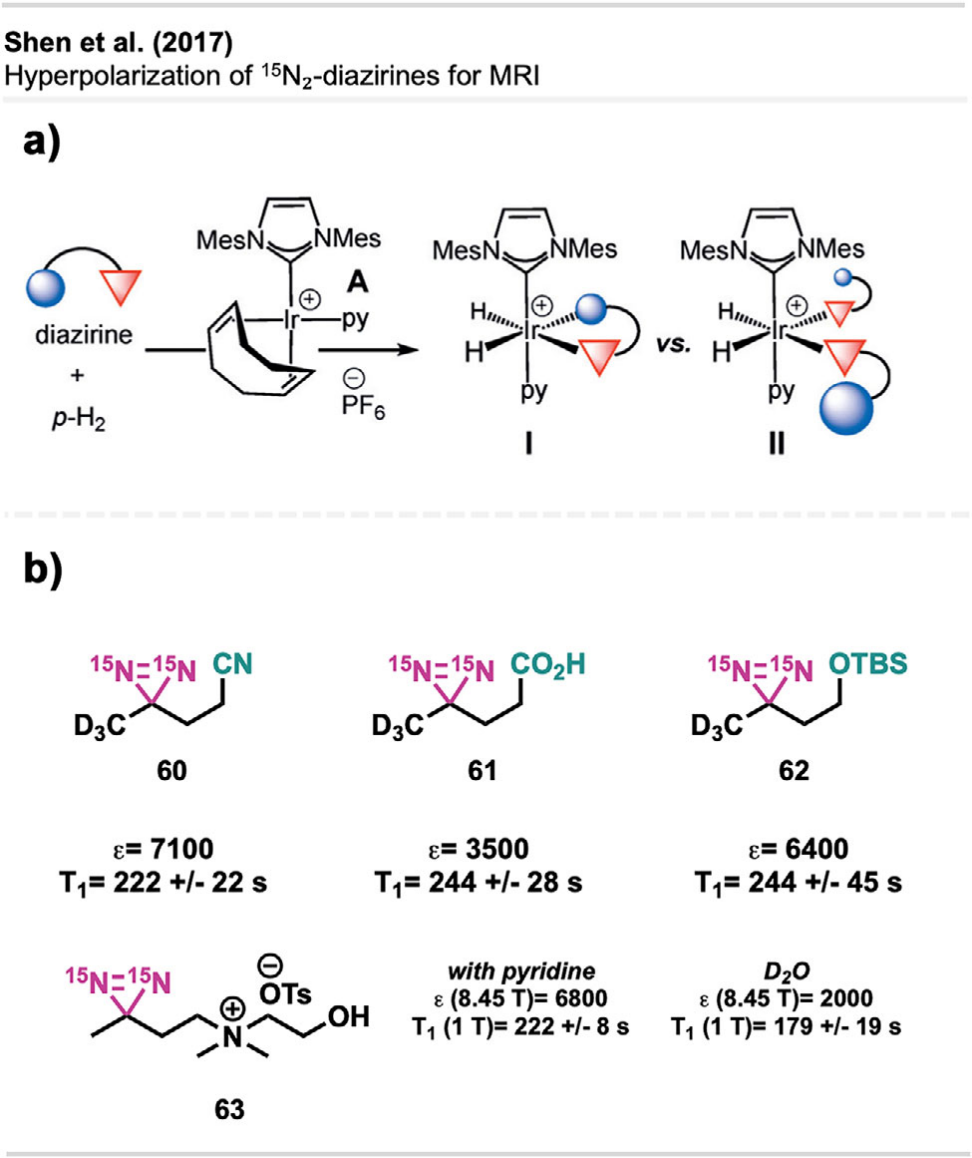
a) HP of ^15^N_2_‐diazirines **60**–**62** under Ir‐catalysis using para hydrogen. b) functionalized ^15^N diazirines and their respective signal enhancement (ε) and T1 relaxation times. The figure in section (a) is a direct reproduction from Shen et al.^[^ ^196^ ^]^ © 2017 Wiley.

In a follow‐up study, since the substrate's coordination to the iridium catalyst is essential for HP by SABRE‐SHEATH, Shen et al. sought to examine if the diazirine moiety alone is capable of binding to iridium or if other structural features are crucial.^[^ ^196^ ^]^ Hence, they synthesized a set of structural ^15^N‐diazirine analogs (Figure 58) and systematically analyzed the contribution of each functional group in successful diazirine HP. By testing the different substrates, they could examine whether chelation to the metal is necessary for HP (mode I) or if the binding of the diazirine to iridium is sufficient (mode II) (Figure 58a). When they subjected diazirine substrates **60**–**62** to standard SABRE‐SHEATH conditions with pyridyl Ir catalyst A, they observed efficient HP of all compounds and a *T*_1_ of 3–4 min at 1 T. Silyl ether **62** was chosen to sterically and electronically prevent its coordination to the metal center (Figure 58b).

Since the differences in signal enhancement and T_1_ were relatively small, they reasoned that chelation is not necessary to attain HP, and the coordination of diazirine to the metal center (mode II) is responsible for HP (Figure 58).

Moreover, they sought to demonstrate the feasibility of ^15^N_2_‐diazirines as molecular tags for biologically relevant molecules. Therefore, they synthesized ^15^N_2_‐diazirine‐tagged choline derivative **63** and subjected it to SABRE‐SHEATH HP with Ir precatalyst B in the presence of pyridine or D_2_O as the Lewis base. The HP resulted in more than 2000‐fold signal enhancement with a *T*_1_ of about 3 min, comparable to when choline **63** was subjected to HP by dynamic nuclear polarization. However, SABRE‐SHEATH is operationally a more straightforward and more economical protocol.

Although the efficient and long‐lasting polarization in the presence of D_2_O represented a considerable achievement for potential biomedical in vivo applications, the ∼3.5‐fold decrease in signal enhancement compared to the D_4_‐MeOH/pyridine system prompted the group around Warren to investigate the HP of ^15^N_2_‐diazirines by dissolution dynamic nuclear polarization. While HP by SABRE is limited mainly to organic solvents due to poor solubility of the polarizing partners in water, in d‐DNP, the hyperpolarized sample is dissolved in superheated solvents (e.g., water), resulting in enhanced nuclear polarization in an aqueous solution.

**Figure 59:**
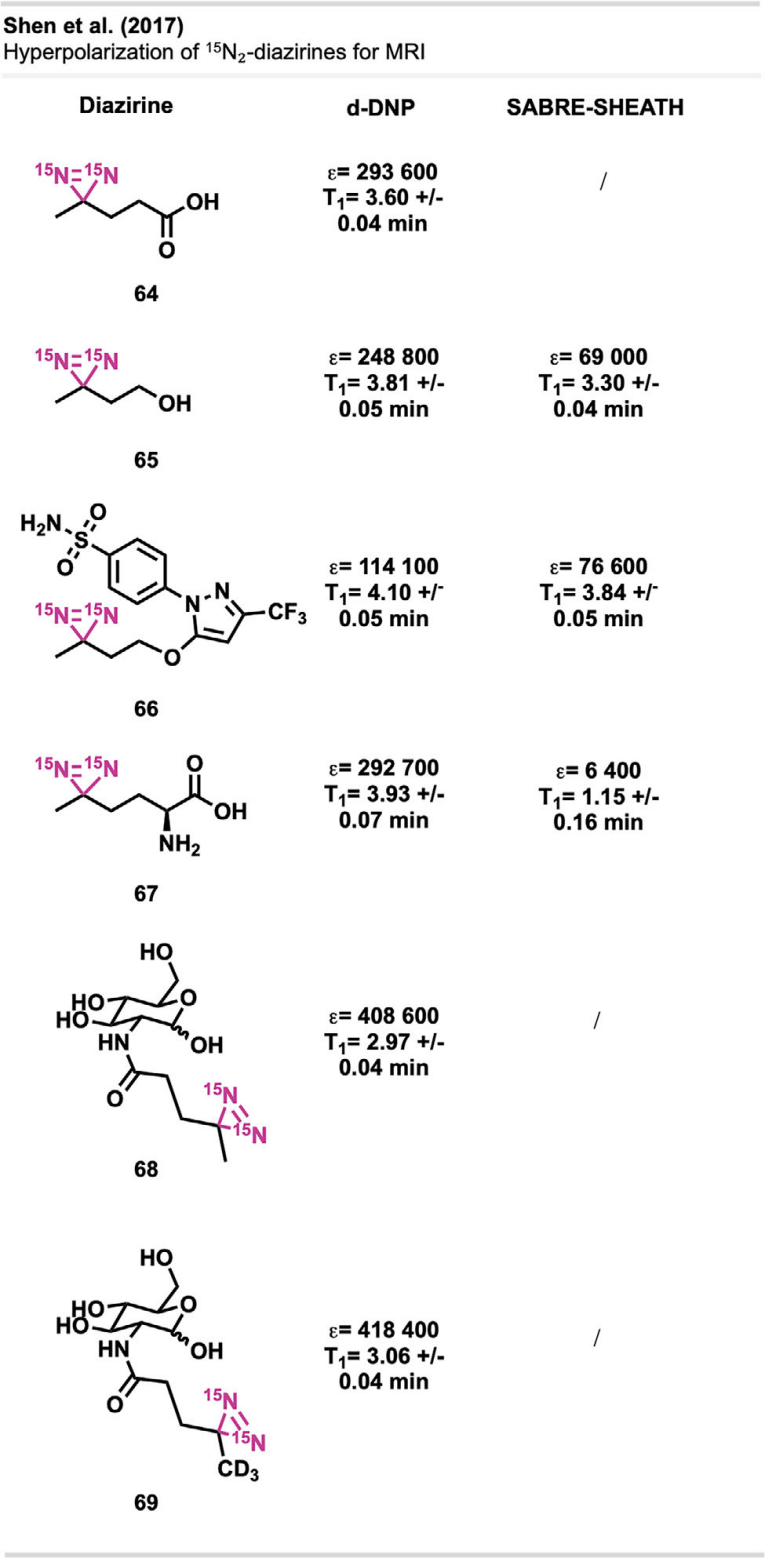
Synthesized ^15^N_2_‐diazirine analogues **64**–**69** and their respective signal enhancement and T_1_ times upon d‐DNP and SABRE‐SHEATH (where applicable) polarization.

In this work,^[^ ^197^ ^]^ Warren et al. synthesized a set of ^15^N_2_‐diazirines **64**–**69**, including biologically important molecules (Figure 59). To test the feasibility of ^15^N_2_‐diazirines‐tagged molecules for in vivo imaging applications, the compounds were subjected to d‐DNP HP in D_2_O. As a result, the authors observed an efficient signal enhancement up to over 400 000‐fold and long relaxation lifetimes up to 4 min of the ^15^N_2_‐diazirine moiety in an aqueous system, implying ^15^N_2_‐diazirine tags as possible candidates for in vivo application in biomedical and clinical research.

In conclusion, the group around Warren demonstrated the feasibility of ^15^N_2_‐diazirines as molecular tags for in vivo imaging applications. ^15^N_2_‐diazirines offer desirable biocompatibility, ease of synthetic excess, and the ability to deliver long‐lasting polarization; thus, it is likely to see their expanded utilization in biomedical and clinical applications in the near future.

## 6 Remarks and Conclusion

Since their discovery in the 1950s, diazirines have primarily been used in chemical biology and to probe the reactivity of specific carbenes within the domain of physical organic chemistry. Despite this, their potential as versatile tools across diverse areas of chemical sciences has been largely underappreciated for much of the past 70 years. Over the last 15 years, diazirines have emerged as more than mere investigative tools, gaining significant traction and finding widespread application in materials science and synthetic chemistry. Numerous, high‐impact publications have highlighted their utility as highly effective crosslinkers in materials functionalization, key components in the development of bioadhesives, and powerful agents in surface modification and microelectronics assembly. Moreover, they have proven to be versatile and valuable carbene precursors, nitrogen sources in synthetic organic chemistry, and potent HP agents in NMR.

Nevertheless, several challenges remain with this class of compounds, limiting their widespread adoption. The synthesis of diazirines is often cumbersome, typically requiring multiple steps and the use of liquid ammonia. While convenient one‐pot methods have been developed for the preparation of aliphatic diazirines, aryl diazirines—particularly the widely used trifluoromethylphenyl diazirine—still rely on multi‐step procedures involving liquid ammonia. Therefore, the development of one‐pot methodologies for the synthesis of aryl diazirines could significantly enhance their accessibility and promote broader adoption.

Although diazirines are generally regarded as safer alternatives to their diazo counterparts with respect to explosive behavior and shock sensitivity, certain structural variants can still exhibit explosive tendencies. While several widely used diazirine crosslinkers, particularly those developed in the Wulff laboratory,^[^ ^37^, ^51^ ^]^ have been evaluated for their energetic properties, a comprehensive and systematic investigation of their stability and potential hazards, especially in bulk quantities, remains absent from the current literature. Arguably, the perception of diazirines as hazardous to handle, combined with the synthetic challenges and associated costs of their preparation, continues to limit their broader adoption in materials science, industry, and chemistry in general.

While limitations in synthetic accessibility and safety are undeniable drawbacks, the steadily increasing number of reports employing diazirines beyond their traditional role in PAL highlights their growing significance and versatility across various areas of the chemical sciences. It can be anticipated that continued research efforts, particularly toward the discovery of new activation strategies and the rational design of diazirines with reactivity optimized for specific applications, will further expand their applicability and unlock new opportunities for their use. Moreover, further advancements in the commercialization of diazirine‐derived technologies are anticipated, particularly in applications as polymer crosslinkers and in medically used bioadhesives.

Although diazirines are increasingly observed in applications that exploit carbene chemistry or address challenges amenable to carbene‐based solutions, their full potential remains to be realized, and their utility is expected to expand further.

With this review, we aim to provide a comprehensive overview of the versatility of diazirines beyond their well‐established applications, hoping to inspire new and creative ideas and future research on this fascinating class of compounds.

## Conflict of Interests

The authors declare no conflict of interest.

## Data Availability

Data sharing is not applicable to this article as no new data were created or analyzed in this study.
